# Giant bulk photovoltaic effect in an iron-based magnetic semiconductor

**DOI:** 10.1038/s41467-026-74473-3

**Published:** 2026-06-16

**Authors:** Mingliang Cheng, Zheng Wang, Jingyu Ji, Jianzhao Wang, Yiting Mo, Yijun Huang, Zhenhua Zhang, Chenxi Lu, Senjiang Yu, Xinglong Dong, Liang Hu, Xuefeng Zhang

**Affiliations:** 1 Zhejiang Key Laboratory of Energy Conversion Materials for Advanced Motor, Institute of Advanced Magnetic Materials, College of Materials and Environmental Engineering, Hangzhou Dianzi University, Hangzhou, PR China; 2 School of Materials Science and Engineering, Dalian University of Technology, Dalian, PR China; 3 Institute for Advanced Study, Shenzhen University, Shenzhen, PR China; 4 Kunming Institute of Physics, Kunming, PR China; 5 State Key Laboratory of Silicon and Advanced Semiconductor Materials, Zhejiang University, Hangzhou, PR China

**Keywords:** Electronic devices, Magnetic devices

## Abstract

Efficient bulk photovoltaic (BPV) conversion and room-temperature ferromagnetism are difficult to combine, because the itinerant electrons that support magnetic order favor metallic transport, whereas BPV generation requires a semiconducting state with broken inversion symmetry. Here, we show that oxygen-plasma implantation transforms metallic Fe_3_GaTe_2_ into a ferromagnetic semiconductor with a giant BPV response, enabling zero-bias photocurrent generation in a non-centrosymmetric lattice. Oxygen incorporation localizes itinerant Fe *d*-electrons, induces p-type semiconducting transport and polar electronic structure, while oxygen-associated exchange pathways allow persistent ferromagnetic state above room temperature. The resulting devices exhibit spontaneous broadband photoresponse, with short-circuit current densities approaching 30 A cm^−2^ and a BPV coefficient up to 0.25 V^−1^. The photovoltaic current can be linearly programmed by low magnetic fields based on field-dependent magnetoresistive modulation. Using the experimentally calibrated device response, we demonstrate magnetically programmable feature separation and image restoration with 92.3% recognition accuracy, establishing oxygen-engineered Fe_3_GaTe_2_ as a platform for self-powered, reconfigurable magnetic optoelectronics.

## Abstract (web summary)

The integration of magnetic ordering with photoelectric conversion holds great promise for optoelectronic systems. Cheng et al. report oxygen-engineered Fe_3_GaTe_2_ as a room-temperature magnetic semiconductor, showing a bulk photovoltaic effect coefficient of 0.25 V⁻¹ and magnetic-field-tunable optoelectronic functionalities.

## Introduction

The integration of magnetic ordering with semiconducting functionalities provides a promising route toward multifunctional optoelectronic systems^1–8^. Magnetic semiconductors, encompassing both diluted and concentrated magnetic variants^9,10^, offer opportunities to couple photoelectric responses with magnetic degrees of freedom. Nevertheless, efficient photoelectric conversion and robust room-temperature magnetic order remain difficult to achieve simultaneously. In many magnetic materials, strong magnetic order is associated with itinerant carriers or metallic electronic structures, whereas efficient optoelectronic operation generally requires controlled semiconducting transport. Existing magnetic semiconductors still face challenges such as suboptimal photoelectric conversion, low Curie temperatures (*T*_C_), and limited compatibility with existing device technologies^11^. Additionally, the presence of magnetic dopants and lattice imperfections diminishes carrier mobility, leading to weakened optoelectronic performance^12–14^. Intrinsic van der Waals magnetic semiconductors and their heterostructures exhibit magnetic-order-related photocurrent responses, but often suffer from environmental instability and typically require large magnetic fields to manipulate their magnetic states^15–20^. Furthermore, their constrained bandgap restricts the spectrum of photon energies they can exploit^21,22^. Therefore, achieving efficient, self-powered photoelectric conversion while retaining room-temperature magnetic order remains a central challenge for magnetic optoelectronic materials.

The bulk photovoltaic (BPV) effect is currently a topic of significant interest due to its wide spectral response beyond the bandgap and the spontaneous separation of photo-generated carriers without the need for conventional p-n junctions^23–26^. This effect fundamentally originates from inversion symmetry breaking, which enables the generation of shift current in natural non-centrosymmetric materials^27–31^. In addition, substantial efforts have been devoted to inducing inversion symmetry breaking in layered materials using approaches such as strain gradients^32–36^, topological effect^37,38^, ionic migration^39–41^, interface engineering^42–46^, and magnetic order^18,20,47^. Despite these advances, achieving large BPV responses under practical conditions remains challenging. Conventional bulk ferroelectrics typically exhibit relatively small BPV coefficients^25,27,30^, while low-dimensional nanostructures often require complex fabrication processes or lack additional functional properties^23,29,31,43,46,47^. To date, only chromium-based layered magnetic semiconductors have experimentally demonstrated the coexistence of the BPV effect and magnetic order^18,47^, albeit at cryogenic temperatures. Obstacles such as thermally induced demagnetization and complex magnetic phase transitions hinder their operation under ambient conditions^15,18,47^. Layered iron-based magnets typically exhibit higher *T*_C_ compared to chromium-based counterparts^48–50^, yet inducing BPV responses in iron-based systems while maintaining magnetic order poses significant hurdles.

In this study, we select layered metallic Fe_3_GaTe_2_ (FGaT) as a model to illustrate the potential of artificially reducing symmetry to induce the BPV response. FGaT is distinguished among all iron-based layered magnets for its strong itinerant ferromagnetism, which remains stable up to 350-380 K in bulk form^48^. Thinning or oxidation can induce semiconductor-like behaviors in these systems^50–54^, but at the expense of weakening magnetic exchange interactions, resulting in *T*_C_ below 300 K. The diminished magnetization can be restored through strain or charge transfer, which are intricately associated with the modulation of itinerant Fe *d*-electrons^55–58^. To generate spontaneous electric dipoles in FGaT, these itinerant electrons can be localized by incorporating electron acceptors^57^. The localization-correlated atomic displacements redistribute charge densities, resulting in a shift current, a fundamental mechanism underlying the BPV effect^35^. Oxygen plasma, containing electron-withdrawing species^59^, serves as an effective source of acceptors to interact with intralayer *d*-electron centers. Engineering the band structures of van der Waals materials is also a key aspect of this strategy^59–61^. The present work introduces an oxygen plasma implantation technique to enable a giant BPV response in FGaT, showing three key outcomes: (1) Symmetry-breaking-induced BPV effect. Intralayer oxygen incorporation disrupts inversion symmetry, generating non-centrosymmetric polar structures and spontaneous broadband photocurrents. (2) Bandgap opening in FGaT. Oxygen-related acceptors deplete itinerant *d*-electrons near the Fermi level (*E*_F_), promoting a metal-to-semiconductor transition, as validated through optical, electrical measurements, and theoretical modeling. (3) Preservation of magnetic ordering. Oxygen implantation contributes to an alternative indirect superexchange pathway (Fe_I_-O-Fe_II_) while not fully suppressing the original magnetic interactions. These combined effects enable the coexistence of BPV response, semiconducting behavior, and room-temperature ferromagnetism within a single-phase material, providing a platform for integrated photo-magnetoelectric functionalities.

## Results

### Oxygen plasma implantation

**Fig. 1:**
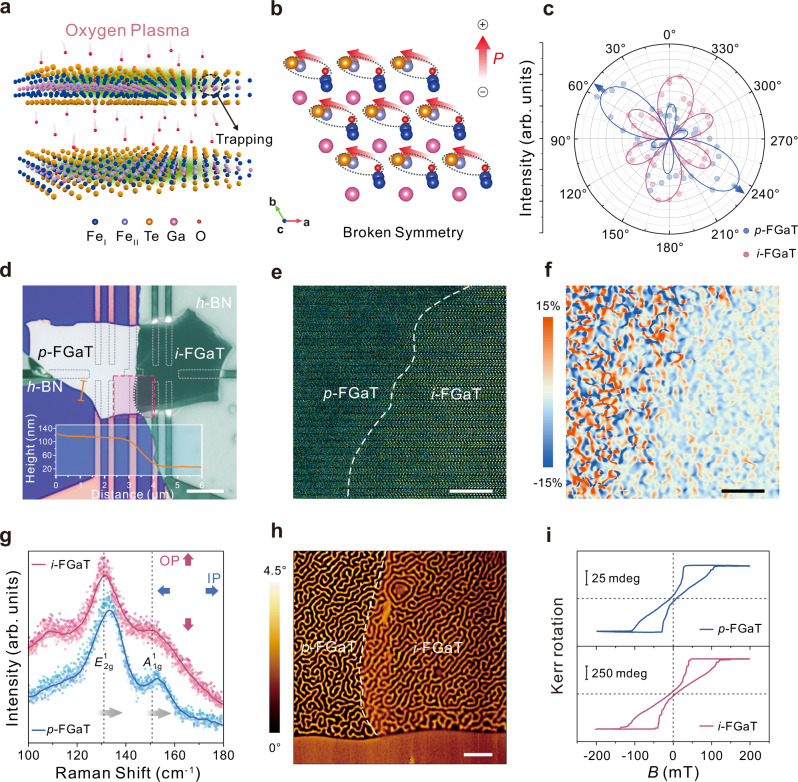
Structural and magnetic characterizations of oxygen-plasma-treated flakes. **a** Schematic of plasma-mediated oxygen implantation in Fe_3_GaTe_2_ (FGaT). Iron atoms occupying two distinct crystallographic sites are depicted as blue and purple spheres; tellurium, gallium, and oxygen atoms are represented by orange, magenta, and red spheres, respectively. **b** Illustration of the oxygen-induced electric polarization mechanism. **c** Normalized polarized second-harmonic generation (SHG) signal patterns to compare the difference of inversion symmetry between initial and plasma-treated FGaT (denoted as *i*- and *p*-FGaT, respectively), with angle 0° corresponding to the horizontal direction to the right in (**d**). **d** Optical micrograph of dual-region Hall device. An entire FGaT flake is composed by *i*- and *p*-FGaT segments. The hexagonal boron nitride (*h*-BN) layer serves as a plasma-resistant mask to protect the *i*-FGaT region, while the exposed region is subjected to oxygen plasma treatment to form *p*-FGaT. Their electrical contacts are achieved on two sets of bottom Hall electrodes highlighted in gray dashed lines, respectively. Inset corresponds to the atomic force microscopy (AFM) height profile of the FGaT flake. **e** Cross-sectional scanning transmission electron microscopy (STEM) image revealing oxygen-induced lattice strains, which are evidenced by geometric phase analysis (GPA) in (**f**). **g** Raman spectral evolution of FGaT flakes before and after oxygen plasma treatment. Ten spectral data are collected in each of two FGaT regions. Inset shows schematic demonstration of in-plane (IP, ${E}_{2{\mbox{g}}}^{1}$) and out-of-plane (OP, ${A}_{1{\mbox{g}}}^{1}$) Raman vibrational modes. **h** Room-temperature magnetic force microscopy (MFM) image displaying labyrinthine magnetic domain structure, corresponding to magenta dashed area in (**d**). **i** Room-temperature magneto-optical Kerr effect (MOKE) hysteresis loo*p*s of *i*-FGaT and *p*-FGaT. Scale bars, 10 μm in (**d**), 5 nm in (**e**) and (**f**), and 2 μm in (**h**), respectively.

Figure 1a illustrates the mechanism of plasma-mediated oxygen implantation into the intact FGaT lattice. The electron-withdrawing oxygen species generated in the plasma preferentially interact with intralayer Fe and Ga sites, leading to oxygen incorporation within the Fe_3_Ga slabs. This occurs via the longitudinal oscillation of oxygen plasma in an inductively coupled plasma (ICP) system operating in capacitive discharge mode (details see Methods and Supplementary Note 1). This process differs from conventional oxidation^53,62^, in which molecular oxygen tends to react first with Te-terminated surfaces and can lead to oxide phases or antiferromagnetic oxidized products (commonly referred to as *o*-FGaT). Structural relaxation disrupts the in-plane rotational symmetry (*D*_3h_) of initial FGaT (denoted as *i*-FGaT), forming a non-centrosymmetric polar structure in plasma-treated FGaT (denoted as *p*-FGaT), as depicted in Fig. 1b, which is expected to support a BPV response. The uniaxially elongated polarization-resolved second-harmonic generation (SHG) pattern shown in Fig. 1c indicates a reduction in crystal symmetry after oxygen plasma treatment, consistent with the formation of non-centrosymmetric polar structures. To enable in situ structure-property correlation studies on FGaT, we employ a spatially selective plasma implantation approach. As shown in Fig. 1d, a mechanically exfoliated FGaT nanoflake was initially placed onto a prepatterned SiO_2_/Si substrate with two sets of independent Hall electrodes, ensuring consistent measuring conditions. For controllable implantation, we use thick hexagonal boron nitride (*h*-BN) as a resist layer to create protected *i*-FGaT and exposed *p*-FGaT regions on the same nanoflake. This method ensures both regions maintain identical crystal quality and thickness, thereby eliminating the batch-to-batch variations common in traditional comparative studies. The inset presents an atomic force microscopy (AFM) line profile along the indicated orange line, showing a FGaT nanoflake thickness of ~90 nm.

The characteristics of initial FGaT bulk and delaminated flakes are extensively analyzed in Supplementary Figs. 1–5, elucidating the layered metallic ferromagnetic nature with a *T*_C_ well above 350 K. Each Fe_3_Ga layer is surrounded by Te terminations, while the FGaT layers are divided by a van der Waals gap of ~0.8 nm^48,55^. A cross-sectional STEM image in Fig. 1e depicts the interface between *i*- and *p*-FGaT regions, revealing that plasma implantation induces a mildly disordered layered structure compared to the original (Supplementary Fig. 6). The difference of contrast possibly indicates the variation of atomic density and/or species. GPA analysis in Fig. 1f shows local strain variations of up to ~15% near the treated region. Meanwhile, the van der Waals gap and overall flake thickness remain largely unchanged (Supplementary Fig. 7), suggesting that oxygen plasma treatment does not lead to substantial interlayer O_2_ molecular intercalation^59,61^. Distorted Fe_3_Ga atoms and intralayer oxygen accumulation (Supplementary Fig. 8) further support the favorable interaction of oxygen plasma with Fe_3_Ga layers. Oxygen atomic inclusions lead to noticeable compressive strains in *p*-FGaT, resulting in a blueshift of ~3 cm^−1^ in wavenumber for the in-plane (${E}_{2{\mbox{g}}}^{1}$) and out-of-plane (${A}_{1{\mbox{g}}}^{1}$) Raman vibrational modes (Fig. 1g)^63^. The MFM image indicates that the labyrinthine domain pattern persists post-plasma implantation (Fig. 1h), with minimal changes in domain widths (Supplementary Fig. 9). Similar observations in other *p*-FGaT samples suggest a thickness-dependent domain structural evolution under zero-field (Supplementary Fig. 10), implying a robust remanence in *p*-FGaT. Magneto-optical Kerr effect (MOKE) measurements at 300 K in Fig. 1i reveal a ten-fold reduction in saturation magnetization (*M*_s_) with coercivity remaining approximately 8 mT. This change is likely due to enhanced perpendicular magnetic anisotropy (PMA) in *p*-FGaT, as evidenced by a stronger coercivity at cryogenic temperatures (see Supplementary Fig. 11). The PMA constant (*K*_u_) ratio between *i*- and *p*-FGaT decreases from about 12.5 to 7.3 with temperature reduction, calculated using Eq. (1) ^48,63^:

1 $${K}_{{{\rm{u}}}}=({B}_{{{\rm{s}}}}{M}_{{{\rm{s}}}})/{2}{\mu }_{0}$$

where *B*_s_ is the saturation field and *μ*_0_ is the vacuum magnetic permeability. The increased magnetic anisotropy suggests a distinct origin for the preserved ferromagnetism, highlighting oxygen plasma implantation as an effective approach for modulating magnetic order in FGaT. Crucially, this magnetic evolution is highly reproducible across five independent devices (Supplementary Fig. 12), where the preserved coercivity is further supported by defect-induced domain wall pinning.

### Theoretical insights into the magnetic semiconductor

**Fig. 2:**
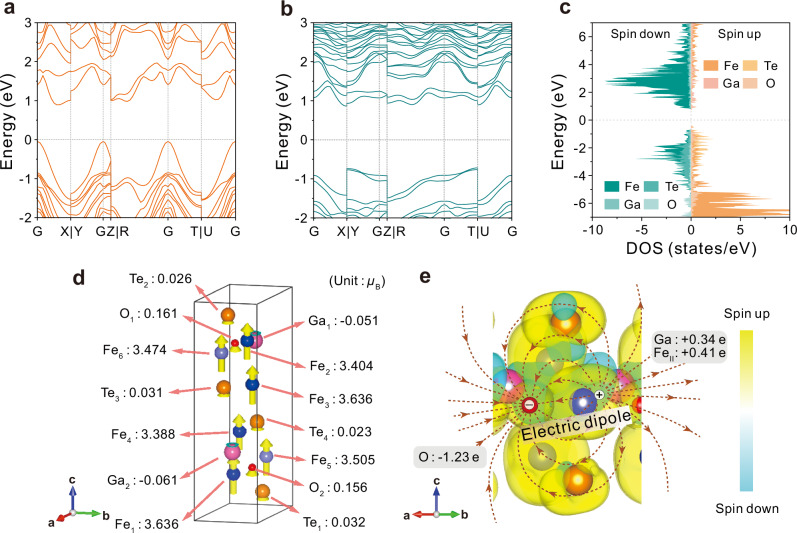
First-principles calculations and polar analysis of intralayer-oxygen-implanted *p*-FGaT. Spin-majority (up) (**a**) and spin-minority (down) (**b**) energy band structures, both indicating an indirect semiconducting nature. **c** Spin-polarized density of states (DOS) diagram. Elemental contributions are represented by gradient colors. **d** Calculated Bohr magnetic moments and spin vector arrows of different atoms in the supercell. **e** Schematic local electric dipole superimposed on the calculated spin-density distribution. The Bader charges of Fe_II_, Ga, and O atoms are indicated.

To investigate the band structures and magnetic properties, the first-principles calculations are conducted on three distinct configurations: *i*-FGaT, and two variations of *p*-FGaT with oxygen atoms positioned at interlayer and intralayer sites. Figure 2a, b presents the opened bandgaps when oxygen atoms are placed in the vicinity of the Fe_3_Ga layers. For each spin channel, the valence band maximum (VBM) and conduction band minimum (CBM) are situated at distinct positions in *k*-space, manifesting this material as an indirect semiconductor. Notably, the *E*_F_ resides closer to the VBM, suggesting p-type semiconducting behavior. The spin-polarized DOS diagram in Fig. 2c displays an asymmetry between spin-majority and spin-minority channels, consistent with the experimentally observed ferromagnetic ordering in the intralayer-oxygen-implanted *p*-FGaT. Furthermore, the system exhibits bipolar semiconducting characteristics, with majority spins dominating the VBM and minority spins dominating the CBM^14^. In contrast, both *i*-FGaT and the interlayer-oxygen-implanted *p*-FGaT exhibit metallic properties (Supplementary Figs. 13, 14), indicating that interlayer oxygen interstitials minimally affect the metallic states but disturb spins in an antiferromagnetic configuration^53^. Figure 2d illustrates that oxygen atoms participate in magnetic exchange and exhibit a consistent alignment with Fe spins in the ferromagnetic supercell. This establishes a new indirect Fe_I_(↑)-O(↑)-Fe_II_(↑) superexchange pathway distinct from the original Fe(↑)-Ga/Te(↓)-Fe(↑) coupling scenario^64,65^. The increased interatomic distance between Fe_I_ and Fe_II_ from 2.63 Å to 3.15 Å and the Fe_I_-O-Fe_II_ bond angle near 90°, support favorable ferromagnetic exchanges based on the Goodenough-Kanamori-Anderson (GKA) principle^66–68^. The enhanced spin exchange between Fe_I_ and Fe_II_ layers leads to an enhanced out-of-plane spin density distribution, as opposed to the in-plane distribution (Supplementary Fig. 15). A comparison of elemental Bohr magnetic moments (Supplementary Tables 1, 2) shows strengthened Fe spins due to increased Fe^3+^ components after oxygen implantation, as confirmed by the X-ray photoelectron spectroscopy (XPS) data (Supplementary Fig. 16). Charge transfer from Fe, Ga and Te toward O atoms leads to higher oxidation states of the host atoms. The naturally oxidized TeO_x_ layer is significantly inhibited, indicating a potential oxidation resistance ability of *p*-FGaT. Bader charge analysis (Supplementary Table 3) reveals an electron redistribution primarily among O, Fe_II_, and Ga. The establishment of (Fe_II_, Ga)-O electric dipoles in Fig. 2e breaks inversion symmetry in FGaT, providing the microscopic origin for inversion symmetry breaking associated with the BPV response.

### Semiconducting and photovoltaic properties

**Fig. 3:**
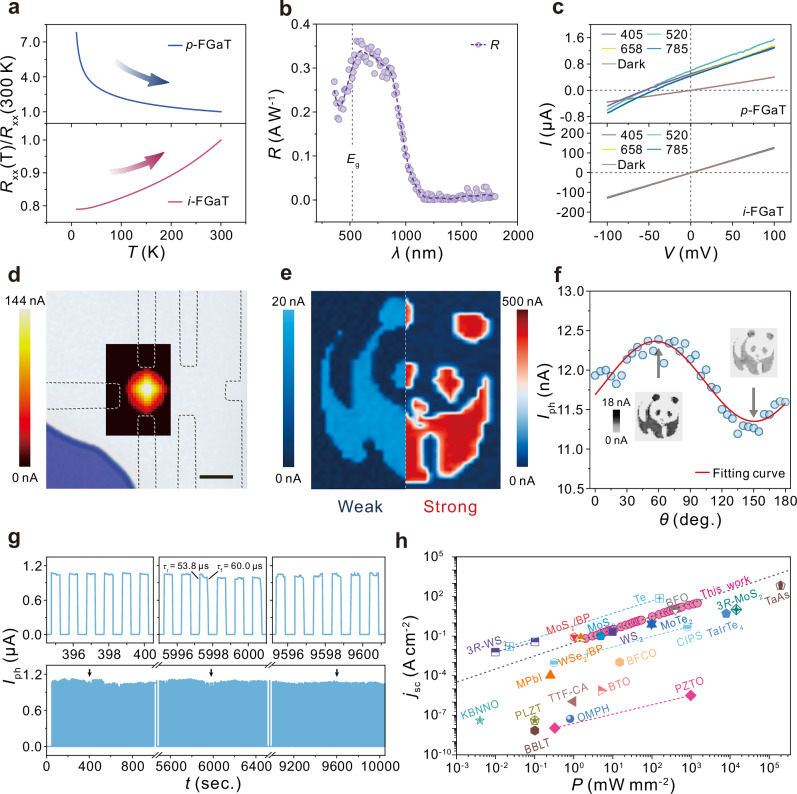
Semiconducting and photovoltaic properties of device. **a** Longitudinal resistance versus temperature (*R*_xx_-*T*) curves of *i*- and *p*-FGaT devices. **b** Wavelength-dependent responsivity (*R*-*λ*) curve of *p*-FGaT device. The optical bandgap (*E*_g_) value is determined as 2.39 eV. **c** Current-voltage (*I*-*V*) curves of *i*- and *p*-FGaT devices under dark and illumination conditions. **d** Spatial photocurrent mapping of the *p*-FGaT device. **e** Incident-power-dependent photoelectric imaging of *p*-FGaT device. **f** Linearly-polarized photocurrents (*I*_ph_-*θ*) and photoelectric imaging of *p*-FGaT device. Insets are the panda-shaped imaging pictures under the polarized angles indicated by arrows, respectively. **g** Endurance test under 10^4^ on/off cycles of *p*-FGaT device. Rise (*τ*_r_) and fall (*τ*_f_) time constants are labeled nearby. **h** Power dependence contrast of short-circuit current density (*j*_sc_-*P*) in reported BPV materials illuminated with different excitation wavelengths. The corresponding data are summarized in Supplementary Table 4. Illumination conditions are as follows: **c** incident light power densities of 372, 276, 278, and 308 mW mm^−2^ at 405, 520, 658, and 785 nm, respectively; **d** *λ* = 520 nm, beam spot diameter = 1~2 μm, illumination power = 235 μW; **e** *λ* = 520 nm, *P* = 5 and 200 mW mm^−2^ for weak and strong illuminations, respectively; **f** *λ* = 520 nm, *P* = 5 mW mm^−2^; **g** *λ* = 520 nm, *P* = 500 mW mm^−2^. Scale bar, 3 μm in (**d**).

We analyze the temperature-dependent resistance of *i*- and *p*-FGaT (Fig. 3a). *i*-FGaT shows metallic conduction, whereas *p*-FGaT displays semiconducting behavior, indicating an oxygen-induced metal-to-semiconductor transition associated with Fe *d*-electron localization and structural disorder^50^. Optimal fitting reveals that the transport behavior follows a three-dimensional Mott variable-range hopping (VRH) mechanism (ln*R*-*T*^−1/4^ scaling; see Supplementary Fig. 17), supporting a bulk effect. Photoluminescence (PL) and ultraviolet photoelectron spectroscopy (UPS) measurements (Supplementary Fig. 18) reveal a well-defined electronic structure with an optical bandgap (*E*_g_) of 2.39 eV, and the CBM and VBM at −3.1 eV and −5.5 eV, respectively. The absence of PL in *i*-FGaT is attributed to its metallic nature. Field-effect transistor characterization (Supplementary Fig. 19) identifies *p*-FGaT as a p-type semiconductor with a hole mobility of 290.7 cm^2 ^V^−1^s^−1^. This performance surpasses that of recent p-type semiconductor candidates such as In_2_Ge_2_Te_6_^69^, *β*-Bi_2_O_3_^70^, and Se-alloyed Te-TeO_x_^71^. As a magnetic semiconductor, *p*-FGaT also demonstrates wavelength (*λ*)-dependent photoelectric conversion from 360 to 1100 nm without bias (Fig. 3b; Supplementary Fig. 20). The peak responsivity (*R*, detailed discussion see Supplementary Fig. 21 and Supplementary Note 2) reaches 0.36 A W^–1^ at 600 nm and gradually decreases toward the near-infrared region, with a cutoff at 1160 nm (~1.07 eV). Notably, the photoresponse extends to photon energies below the optical bandgap (*E*_g_ ≈ 2.39 eV), first-principles calculations using the HSE functional reveal a fundamental electronic gap of ~1.02 eV (spin-up channel), which aligns well with this experimental cutoff. This suggests that the broadband BPV response extending to the near-infrared region can be attributed to the intrinsic electronic structure captured by the hybrid functional theory.

Figure 3c illustrates the *I-V* characteristic curves of the *p*-FGaT device, showing a zero-crossing point in the dark state and an offset under illumination at four typical wavelengths. This anomalous photovoltaic behavior, distinct from a traditional p-n junction or Schottky barrier-induced photovoltaic effect, aligns with the fundamental feature of the BPV effect^72^. In contrast, the *i*-FGaT device exhibits negligible change in *I-V* curves pre- and post-illumination (Supplementary Fig. 22). Power-dependent photocurrent experiments (Supplementary Fig. 23) indicate a shift from a linear to a square-root power dependence, excluding the possibility of interface effects between the electrodes and *p*-FGaT^31,73^. The external quantum efficiency (EQE) achieves an exceptional value of up to 66.4% at 520 nm (at least 30.8% for 785 nm) within the linear power response range of 600 mW mm^−2^, rendering a comprehensive photoelectric conversion capability in *p*-FGaT. Spatial mapping of photocurrents under 520 nm irradiation (Fig. 3d) reveals a uniform current distribution across the electrode channel, supporting a bulk-dominated photoresponse rather than a localized electrode-contact contribution. Comparative photoelectric imaging using a panda-shaped mask (50 × 50 pixels) in Fig. 3e further verifies a wide dynamic response capability under both weak (33.75 nW) and strong (1.35 μW) illuminations. Additionally, polarization-dependent photocurrents exhibit a sinusoidal evolution with a 180° period. When the incident light is linearly polarized along an inversion-symmetry-broken direction (~60° as shown in Fig. 1c), the spontaneous photocurrent is maximized, representing a shift current characteristic of the BPV effect (Fig. 3f)^31,42,43,46,47^. Piezoresponse force microscopy (PFM) measurements (Supplementary Fig. 24) show no switchable ferroelectric response, indicating that the BPV response originates from static oxygen-induced polarity rather than ferroelectric polarization switching. While injection-type contributions are symmetry-allowed in magnetic systems^18,20,47^, their identification requires field-odd photocurrent signatures that are not observed here (as discussed later). Accordingly, the photocurrent is interpreted as being dominated by shift-current generation, with additional modulation arising from magnetic-field-dependent transport.

The durability assessment of the BPV sensor (Fig. 3g) demonstrates robust operational stability at zero bias even after 10^4^ on/off cycles, showing no discernible degradation. The rise (*τ*_r_) and fall (*τ*_f_) time constants of the sensor are 53.8 μs and 60.0 μs, respectively (Supplementary Fig. 25), indicating potential for high-speed sensing applications. Application of a 100 mV bias voltage results in a two-fold increase in photocurrent with less than 1% fluctuation (Supplementary Fig. 26). Reproducibility is further validated on multiple independent samples with varying thicknesses, all of which consistently exhibit robust broadband BPV performance (Supplementary Figs. 27–29). Furthermore, the device demonstrates exceptional long-term environmental robustness. As evidenced by an extended aging test over 18 months in ambient conditions, the sensor retains ~63% of its initial photoresponse (Supplementary Figs. 30–33), underscoring its reliability for practical applications. In comparison to conventional perovskite-type and layered BPV systems (indices see Supplementary Table 4), *p*-FGaT exhibits comprehensive performance advantages, offering a wide spectral coverage from near-infrared to near-ultraviolet bands (Fig. 3h). Under 520 nm excitation, the *j*_sc_ approaches 30 A cm^−2^ calculated by a spot area of ~10 μm^2^. The corresponding BPV coefficient was calculated using Eq. (2):

2 $$\beta={j}_{{{\rm{sc}}}}/P$$

The obtained *β* reaches 0.25 V^−1^, superior to most BPV materials (Supplementary Table 5). Conversely, the preparation of *o*-FGaT through air thermal oxidation yields limited photoelectric gain and slow response dynamics (Supplementary Note 3; Supplementary Figs. 34–36), indicating that the conventional oxidation mechanism fails to explain the observed BPV behavior in *p*-FGaT. The oxygen implantation model illustrated in Fig. 2e effectively elucidates the generation of electric dipoles under mild disorder, aligning well with the observed phenomena.

### Magnetically programmable photosensing

**Fig. 4:**
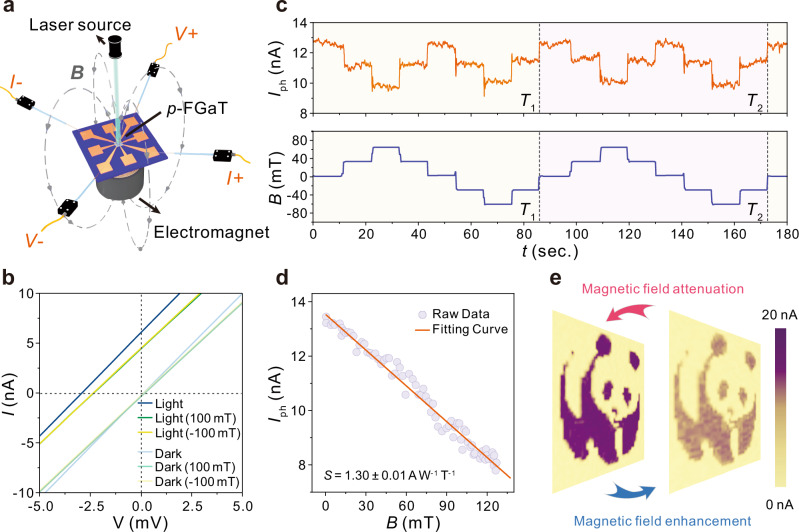
Photo-magnetoelectric response of *p*-FGaT device. **a** Schematic illustration of magnetic field (*B*) modulated photocurrent (*I*_ph_) measurements. **b** Current-voltage (*I*-*V*) curves measured under ±100 mT magnetic field under dark and illuminated conditions. **c** Stepwise programmable magnetic field modulation and simultaneous photocurrent (*I*_ph_) response. *T*_1_ and *T*_2_ refer to the first and second programmable time sequences, respectively. **d** Linear sensing characteristic of *I*_ph_ versus *B* with a sensitivity (*S*) of 1.30 ± 0.01 A W^−1^ T^−1^. **e** Comparison of photoelectric imaging before and after applying a fixed magnetic field (*B* = 120 mT). Incident powers under 520 nm illumination in (**b**) and (**c**–**e**) are 2.5 and 5.0 mW mm^−2^, respectively.

The magnetic field-modulated photocurrent experiment setup (Fig. 4a) involves a 520 nm laser illuminating vertically onto the BPV sensor. An electromagnet positioned below the sensor generates an out-of-plane magnetic field perpendicular to the sensor, with adjustable field strength controlled by the coil current. Current and magnetic field signals are recorded simultaneously by an integrated software system. Figure 4b illustrates a symmetric reduction (~8%) in the dark current of the BPV sensor under ±100 mT magnetic fields, indicating an intrinsic positive magnetoresistance (PMR) effect that remains consistent for both polarities. Upon illumination, the short-circuit photocurrent exhibits a similar symmetric suppression, but with a significantly enhanced modulation depth of ~20% under the same magnetic field (Supplementary Fig. 37). This suggests that non-equilibrium photo-generated carriers are more sensitive to the magnetic-field-induced scattering than thermally relaxed equilibrium carriers. As further elucidated by field-effect transistor (FET) characterizations (Supplementary Fig. 38), the magnetic field leads to a notable degradation in field-effect mobility (*μ*_FE_) while slightly increasing carrier density, supporting mobility suppression as the dominant contribution to the current reduction. Figure 4c depicts variations in photocurrent under periodic magnetic field steps, demonstrating a rapid and reversible response. Beyond the saturation magnetic field (*B*_s_ = 120 mT), the decrease in photocurrent tends to plateau (Supplementary Figs. 39, 40), a behavior that diverges from the photoelectromagnetic (PEM) effect^74^, but closely correlates with the scattering saturation of photo-generated carriers. This behavior does not exhibit the sign reversal expected for injection-type photocurrents, and is instead consistent with a magnetoresistive transport modulation mechanism (Supplementary Fig. 41).

The inversion-symmetry-breaking polar structure provides the basis for the observed BPV response. In this system, symmetry-breaking-driven shift current serves as the primary photocurrent generation mechanism (Supplementary Fig. 42), whereas the magnetic field modulates the photocurrent output through magnetoresistive transport. This picture accounts for the field-symmetric photocurrent suppression and its saturation-like behavior near *B*_s_, as discussed in Supplementary Note 4. A plot of photocurrent against magnetic field demonstrates a linear decrease in current within the magnetic field range *B*_s_ (Fig. 4d). The modulation sensitivity (*S*) was calculated using Eq. (3):

3 $$S=j_{{\rm{sc}}}/{P}_{{\rm{\lambda}}}{B}_{{\rm{s}}}$$

The obtained sensitivity is 1.30 ± 0.01 A W^−1^T^−1^. Despite a noise current at the nA level, the suppression ratio of photocurrent (Δ*I*_ph_/*I*_ph_) can exceed 40% at 120 mT, correlating with incident light power and being influenced by the density of photo-generated carriers. This BPV sensor exhibits a wide linear range and μT-level magnetic-field response, rendering it suitable for self-powered magnetometer applications to monitor magnetic field strength. The modulation magnitude of photocurrent (Δ*I*_ph_/Δ*B* ≈ 45 nA T^−1^ or Δ*j*_sc_/Δ*B* ≈ 450 mA cm^−2^ T^−1^) at room temperature surpasses that of antiferromagnetic semiconductor (~1.2 nA T^−1^)^15^, magnetic van der Waals heterostructure (0.2–4.5 nA T^−1^)^4,16^, manganite-based heterojunction (~6 μA cm^−2^ T^−1^)^9^, and organic spin valve (~1 nA T^−1^)^75^. Furthermore, Fig. 4e illustrates a notable decrease in resolution of the panda imaging pattern in the presence of a commercial mini permanent magnet (Supplementary Fig. 43), suggesting potential applications in in-sensor computing.

**Fig. 5:**
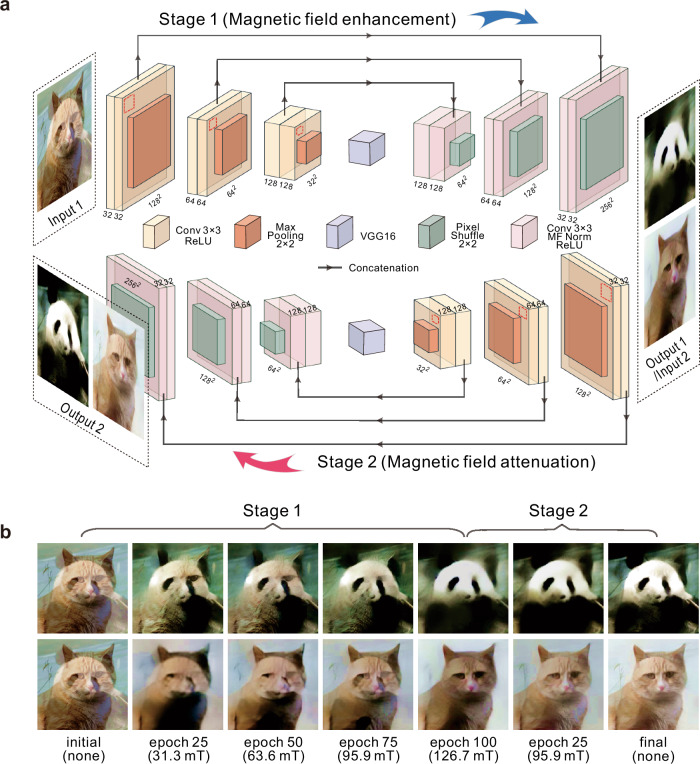
Implementation of magnetically-programmable in-sensor computing for feature separation and restoration. **a** Schematic diagram of the physics-driven convolutional processing workflow. The network leverages the *p*-FGaT device as a physical tunable weight controller to execute a two-stage process: interference suppression (Stage 1) and detail recovery (Stage 2) within a unified framework. **b** Visualization of the magnetically-driven progressive feature disentanglement and restoration. The panels illustrate the evolution of image features under varying magnetic field strength across training epochs (0 → 100 epochs).

Leveraging the magnetic field linear modulation of photocurrents and device stability, we devise a magnetically-programmable neural network (MNN) hardware simulation architecture. Figure 5a outlines the core workflow, where the *p*-FGaT device functions as a physical tunable weight controller, utilizing gradient magnetic constraints to facilitate feature-decoupled separation and image inpainting of hybrid images. In this demonstration, cat-panda hybrid images undergo feature downsampling, followed by a decoding phase using magnetic field enhancement to suppress cat features while preserving panda characteristics, achieving precise feature separation. Additionally, degraded image reconstruction via inverse magnetic field modulation results in high-precision restored images (for details see Supplementary Fig. 44).

To assess system performance, we conduct a proof-of-concept experiment. In Stage 1, the magnetic field is linearly coupled to the image blending ratio *α* (where *B* ∝ *α*) to serve as the governing control variable. Crucially, we translate the device’s signal enhancement into a computational suppression mechanism. Specifically, increasing magnetic field induces negative modulation that reduces feature channel transmission, mimicking photocurrent attenuation. This process effectively fades out interference features (e.g., the cat) in proportion to the applied field (for details see Supplementary Note 5). This progressive disentanglement is visualized in Fig. 5b (left panels), where increasing magnetic field intensities (0 → 100 epochs) drive hierarchical pattern formation as initial blended inputs progress from rough separation (epoch 25) to complete separation (epoch 100), quantified by magnetic field strength during convolutional computation.

This physics-driven logic extends to the restoration phase (Stage 2), where the process is reversed to recover image fidelity. Initially, the output images are compromised by noise artifacts. To address this, the magnetic field is calibrated to the noise level *ω* (where *B* ∝ *ω*), utilizing the device’s signal attenuation to effectively recover high-quality pixels. Subsequently, by progressively diminishing the magnetic field, the suppression features are relaxed, which mimics the recovery of photocurrent as the magnetic field is removed (for details see Supplementary Note 5). This gradual magnetic field reduction allows the previously suppressed fine details to re-emerge while keeping noise minimal, enabling precise image reconstruction. Figure 5b (right panels) tracks this magnetic-attenuation-driven restoration, where diminishing magnetic field over 100 epochs progressively refine the image. Initial restoration of details and colors appears at the 25th epoch, ultimately achieving final realistic restoration (epoch 100) achieved by magnetically controlled multiscale fusion. Collectively, these demonstrations highlight the potential of the material’s robust photo-magnetoelectric response to pave the way for next-generation paradigms in magnetically controlled in-sensor computing.

## Discussion

In summary, this work introduces an oxygen plasma implantation as an effective strategy for creating giant BPV responses in an iron-based van der Waals ferromagnetic semiconductor. Oxygen incorporation induces p-type semiconducting transport, generates non-centrosymmetric polar distortions, and preserves ferromagnetic ordering above room temperature. The resulting *p*-FGaT devices exhibit broadband zero-bias photoresponse, a BPV coefficient of up to 0.25 V^−1^, fast response dynamics, and long-term environmental stability. Magnetic-field-dependent measurements further reveal a linear magnetoresistive modulation of the BPV photocurrent, enabling low-field magnetic-field strength sensing. The magnetic-field-programmable photocurrent response is further used for proof-of-concept neuromorphic image processing, including feature separation and image restoration. Together, these findings show how oxygen-driven symmetry and electronic-structure engineering can transform a room-temperature magnetic metal into a ferromagnetic semiconductor with giant BPV generation and practical magnetic-field-responsive optoelectronic functions.

## Methods

### Single crystal growth

High-quality FGaT single crystals were synthesised via a self-flux method. The stoichiometric mixture of high-purity elemental precursors (Fe:Ga:Te = 1:1:2 molar ratio) was sealed under vacuum in an Ar-purged quartz ampoule. The ampoule was heated to 1273 K within 1 h and maintained at this temperature for another 24 h to achieve complete homogenization. The temperature was then rapidly reduced to 1153 K within 1 h and slowly cooled to 1053 K over 100 h. The grown crystals were stored in an Ar-filled glove box (H_2_O, O_2_ < 0.1 ppm) to minimize surface oxidation.

### Device fabrication

Two sets of axisymmetric Cr/Au (5/20 nm) Hall bar electrodes were pre-fabricated on 300 nm oxide-layered SiO_2_/Si substrates by laser direct writing, electron-beam evaporation, and lift-off processes. Mechanically exfoliated FGaT nanoflakes were transferred onto pre-patterned electrodes via polydimethylsiloxane (PDMS)-assisted dry method. For selective oxygen plasma treatment, a thick *h*-BN flake was used as a plasma-resistant mask to protect one region of the FGaT nanoflake, while the exposed region was subjected to oxygen plasma treatment. Finally, device integration was completed through high-vacuum annealing (423 K, 20 min) to optimize electrical contacts. All lift-off and transfer processes were carried out under inert atmosphere (H_2_O, O_2_ < 0.1 ppm).

### Oxygen plasma treatment

Controlled oxygen incorporation was achieved using an inductively coupled plasma (ICP) reactor operating at 13.56 MHz. Fabricated devices were oriented perpendicular to the direction of plasma flow under optimized conditions: radio frequency (RF) power of 100 W and chamber pressure of 25 Pa under continuous oxygen flow (see Supplementary Note 1 for details).

### Structural characterization

The crystal phase and structure were characterized by powder X-ray diffraction (XRD; Rigaku, Smartlab 9 kW) with Cu *Kα* radiation (*λ* = 1.5418 Å). Morphological features and nanosheet thickness were assessed via optical microscopy (Olympus, BX53M), atomic force microscopy (AFM; Bruker, NanoWizard 4), and transmission electron microscopy (TEM; FEI, Talos F200S). Complementary energy-dispersive X-ray spectroscopy (EDS) provided elemental composition and molar ratios for the establishment of calculated model (Supplementary Fig. 45). Atomic-resolution imaging of the microstructure was carried out using an aberration-corrected TEM (JEOL, JEM-ARM200F NEOARM).

### Spectroscopic characterization

Surface chemical states were analyzed by X-ray photoelectron spectroscopy (XPS; Thermo Scientific, Nexsa). The electronic structure was probed using ultraviolet photoelectron spectroscopy (UPS; Thermo Scientific, Nexsa) with He-I radiation (21.22 eV). Vibrational modes and lattice dynamics were investigated via Raman spectroscopy (Horiba, iHR320) employing a 532 nm excitation laser. Crystalline symmetry was probed through second-harmonic generation (SHG; Meta MStarter 100-SHG) equipment using a 1064 nm ps-pulsed laser.

### Magnetic characterization

Magnetic domain configurations were visualized through Lorentz TEM (FEI, Talos F200S) and magnetic force microscopy (MFM; Bruker, NanoWizard 4). Magnetic hysteresis loops of nanoflakes were quantified using a temperature-dependent magneto-optical Kerr microscope (Truth Instruments, KMP-L). Bulk magnetic properties, including zero-field-cooled/field-cooled (ZFC-FC) curves and magnetization hysteresis (*M*-*H* loops), were measured with a superconducting quantum interference device (SQUID; Quantum Design, MPMS-7T). Temperature-dependent resistance (*R*_*xx*_*-T*) and Hall resistance (*R*_xy_) under applied magnetic fields were characterized via a physical property measurement system (Quantum Design, PPMS-9T).

### Magnetically photoelectric characterization

The magnetic field-modulated photoresponse under multi-wavelength illumination (365–1064 nm) was investigated using an electromagnet system (Yingpu Magnetoelectric, SP40-3000). Optical power density was calibrated using a silicon photodetector (Newport, 918D-UV-0D3R) paired with an optical power meter (Newport, 1919-R). Real-time magnetic flux density was monitored by a Hall-effect gaussmeter (CH-Magnetoelectricity Technology, CH-1600), while photocurrent signals were acquired using a semiconductor parameter analyzer (Tektronix, Keithley 2602B). The photoelectric imaging was performed using the single-point scanning virtual imaging function of the integrated photoelectric measurement system (META, ScanPro Advance). The specific parameters were set as follows: a laser with a wavelength of 520 nm was used as the excitation source and was focused onto the sample surface through a 25× objective lens. The incident light intensities for weak and strong light conditions were precisely set to 5 and 200 mW mm^−2^, respectively. The scanning area was defined as a 50 × 50-pixel array with a step size (pixel-to-pixel distance) of one pixel. The laser beam was delivered via an optical fiber with a core diameter of 400 μm, creating a spot size of ~42.7 μm in diameter on the sample. During the data acquisition, the dwell time (single-point delay) at each pixel was 150 ms. Notably, the entire imaging process was conducted under the self-driven mode, with the source-drain voltage (*V*_ds_) maintained at 0 V. The polarization module of the system was synchronized with a light source measurement unit (Tektronix, Keithley 2400) for polarization-dependent photoresponse characterization under 520 nm laser irradiation. High-resolution photocurrent mapping characterization, response dynamics, durability testing, and wavelength-dependent response spectrum analysis were performed using a high-precision photocurrent scanning test microscope system (META, MStarter 200).

### Theoretical calculations

Spin-polarized density functional theory (DFT) calculations were conducted using the Vienna Ab initio Simulation Package (VASP)^76–78^. These calculations adhered to the Perdew–Burke–Ernzerhof functional level of theory^79^. To accurately model various elements, we employed projector-augmented wave potentials, specifically targeting the orbitals of Fe (3*p*^6^3 *d*^7^4*s*^1^), Te (5*s*^2^5*p*^4^), Ga (4*s*^2^4*p*^1^), and O (2*s*^2^2*p*^4^)^77,80^. To account for the localized nature of Fe 3*d* electrons, the Hubbard *U* correction was applied with an effective *U* (*U*_eff_) value of 4.0 eV^81^. A plane-wave energy cutoff of 700 eV was adopted. The Brillouin zone was sampled using a Γ-centered 15 × 15 × 3 k-point mesh. Structural optimizations were carried out until the forces on all atoms were less than 0.001 eV/Å. To rigorously correct the bandgap underestimation typical of PBE + *U* functionals, we employed the Heyd–Scuseria–Ernzerhof (HSE) hybrid functional with a Hartree–Fock mixing parameter of 0.32. The energy cutoff was set to 600 eV, and a 7 × 7 × 2 k-point mesh was used for these computationally demanding calculations^82–85^. The crystal structures, spin vector arrows, and spin-density distribution shown in Fig. 2d, e were visualized using the VESTA software^86^.

## Supplementary information

Supplementary Information

Transparent Peer Review file

## Data Availability

The data supporting the findings of this study are available within the paper and its Supplementary Information. The source data underlying the main and Supplementary Figs. have been deposited in figshare at 10.6084/m9.figshare.30938402.

## Code availability

The custom code used for the neuromorphic vision simulation has been deposited in figshare at 10.6084/m9.figshare.30938402.
