# Supplementary material for: Giant bulk photovoltaic effect in an iron-based magnetic semiconductor
Source: Nat Commun. 2026 Jun 16;17:7629. doi: 10.1038/s41467-026-74473-3 (PMC13429580; doi:10.1038/s41467-026-74473-3)
Supplement: Supplementary file 1 — Supplementary Information [file 41467_2026_74473_MOESM1_ESM.pdf]

Supplementary Information for

Giant Bulk Photovoltaic Effect in an Iron-Based Magnetic Semiconductor

Mingliang Cheng[1,2†], Zheng Wang[3,4†], Jingyu Ji[1†], Jianzhao Wang[1], Yiting Mo[1], Yijun Huang[1], Zhenhua Zhang[1], Chenxi Lu[1,5], Senjiang Yu[1], Xinglong Dong[2], Liang Hu[1,5]* and Xuefeng Zhang[1]*

[1]Zhejiang Key Laboratory of Energy Conversion Materials for Advanced Motor, Institute of Advanced Magnetic Materials, College of Materials and Environmental Engineering, Hangzhou Dianzi University, Hangzhou 310018, P. R. China
[2]School of Materials Science and Engineering, Dalian University of Technology, Dalian 116023, P. R. China
[3]Institute for Advanced Study, Shenzhen University, Shenzhen 518060, P. R. China
[4]Kunming Institute of Physics, Kunming 650223, China
[5]State Key Laboratory of Silicon and Advanced Semiconductor Materials, Zhejiang University, Hangzhou 310027, P. R. China
[†]These authors contributed equally: Mingliang Cheng, Zheng Wang, Jingyu Ji.

Corresponding authors: huliang@hdu.edu.cn; Zhang@hdu.edu.cn

Supplementary Note 1: Inductively coupled plasma (ICP) implantation details 5
Supplementary Fig. 1 | Crystal characterization of the van der Waals layered FGaT single crystals. 5
Supplementary Fig. 2 | Temperature-dependent magnetization measurements of FGaT bulk crystals using superconducting quantum interference (SQUID) device...6
Supplementary Fig. 3 | Transmission electron microscopy (TEM) characterization of a pristine FGaT nanoflake...7
Supplementary Fig. 4 | Lorentz transmission electron microscopy (LTEM) characterization of a pristine FGaT nanoflake..8
Supplementary Fig. 5 | Anomalous Hall measurements of a pristine FGaT nanoflake.....................8
Supplementary Fig. 6 | Cross-sectional TEM images of *i*-FGaT. 8
Supplementary Fig. 7 | Atomic force microscopy (AFM) characterization of FGaT nanoflakes before and after oxygen plasma implantation. 9
Supplementary Fig. 8 | High-resolution STEM characterization of *p*-FGaT....................................9
Supplementary Fig. 9 | Magnetic domain width statistics of *i*-FGaT and *p*-FGaT............................9
Supplementary Fig. 10 | Thickness-dependent magnetic domain structures of *p*-FGaT nanoflakes. 10
Supplementary Fig. 11 | Temperature-dependent magneto-optical Kerr effect (MOKE) signal comparison...10
Supplementary Fig. 12 | Magnetic evolution of *p*-FGaT under cumulative oxygen plasma treatment. 12
Supplementary Fig. 13 | Density functional theory (DFT) calculations of intrinsic *i*-FGaT...........12
Supplementary Fig. 14 | DFT calculations of interlayer-oxygen-implanted FGaT. 13
Supplementary Fig. 15 | Spin density distributions of FGaT before and after intralayer oxygen implantation. 13
Supplementary Table 1 | Calculated elemental magnetic moments (unit: μ_B) of intralayer-oxygen-implanted FGaT...14
Supplementary Table 2 | Calculated elemental magnetic moments (unit: μ_B) of *i*-FGaT...............15
Supplementary Fig. 16 | X-ray photoelectron spectroscopy (XPS) characterization of FGaT nanoflakes. 16
Supplementary Table 3 | Bader charges (*e*) of intralayer-oxygen-implanted FGaT, calculated using DFT. 17
Supplementary Fig. 17 | Mott variable range hopping (VRH) fitting of the *p*-FGaT.....................18
Supplementary Fig. 18 | Electronic structure characterization of *p*-FGaT......................................18
Supplementary Fig. 19 | Field-effect transistor (FET) performance evaluation.............................19
Supplementary Fig. 20 | Broadband photoresponse of *p*-FGaT sensor (365-1064 nm).................19
Supplementary Fig. 21 | Wavelength-dependent optoelectronic characteristics of the *p*-FGaT device. 20
Supplementary Note 2: Details on the calculation of responsivity (*R*) 21
Supplementary Fig. 22 | Wavelength-dependent photocurrent response curves of *i*-FGaT device under 0.1 V bias. 21
Supplementary Fig. 23 | Short-circuit current density (j_{sc}) versus optical power of the *p*-FGaT device illuminated by different light sources..22
Supplementary Fig. 24 | Piezoresponse force microscopy (PFM) characterization of *p*-FGaT. 22

Supplementary Fig. 25 | Rise and fall dynamics of the short-circuit photocurrent under 520 nm illumination. 22
Supplementary Fig. 26 | Photocurrent response curves of *p*-FGaT device under 0.1 V bias conditions with modulated 520 nm excitation pulses (f = 1 Hz, P = 500 mW mm^{-2}). 23
Supplementary Fig. 27 | Photovoltaic performance of *p*-FGaT Device #1 (~105 nm). 24
Supplementary Fig. 28 | Photovoltaic performance of *p*-FGaT Device #2 (~30 nm). 25
Supplementary Fig. 29 | Photovoltaic performance of *p*-FGaT Device #3 (~73 nm). 26
Supplementary Fig. 30 | Photocurrent response curves of *p*-FGaT device under self-driven and 0.1 V bias conditions with modulated 405 nm excitation pulses (f = 1 Hz, P = 570 mW mm^{-2}). 27
Supplementary Fig. 31 | Photocurrent response curves of *p*-FGaT device under self-driven and 0.1 V bias conditions with modulated 658 nm excitation pulses (f = 1 Hz, P = 415 mW mm^{-2}). 28
Supplementary Fig. 32 | Photocurrent response curves of *p*-FGaT device under self-driven and 0.1 V bias conditions with modulated 785 nm excitation pulses (f = 1 Hz, P = 460 mW mm^{-2}). 29
Supplementary Fig. 33 | Long-term environmental stability of the *p*-FGaT device. 30
Supplementary Table 4 | Performance comparison of characteristic response wavelengths and short-circuit photocurrent densities among previously reported BPV materials. 31
Supplementary Table 5 | Comparison of the BPV coefficients of *p*-FGaT with previously reported materials. 32
Supplementary Note 3: Preparation details of thermally-oxidized FGaT (*o*-FGaT) 33
Supplementary Fig. 34 | Structural characterization of thermally-oxidized FGaT (*o*-FGaT). 33
Supplementary Fig. 35 | Morphology and magnetic characterization of *o*-FGaT. 34
Supplementary Fig. 36 | Photocurrent response curves of *o*-FGaT device under 0.1 V bias condition illuminated by different light sources 34
Supplementary Fig. 37 | Comparative analysis of magnetic field-dependent transport in dark and illuminated states 35
Supplementary Fig. 38 | Transfer characteristics (I_{DS}~V_{GS}) of the *p*-FGaT field-effect transistor (FET) under zero magnetic field (B = 0 mT) and an applied perpendicular magnetic field (B = 100 mT). 35
Supplementary Fig. 39 | Magnetic-field-dependent photocurrent modulation under sinusoidal field excitation. 37
Supplementary Fig. 40 | Photocurrent response under magnetic field. 37
Supplementary Fig. 41 | Schematic illustration of magnetic-field modulation of photocurrent in *p*-FGaT devices. 37
Supplementary Note 4: Mechanism of magnetic-field-modulated photocurrent 38
Supplementary Fig. 42 | Circularly polarized photocurrent (I_{ph}-θ) of the *p*-FGaT device (λ = 638 nm, P = 10 mW mm^{-2}). 39
Supplementary Fig. 43 | Digital photograph of photoelectric imaging equipment under an applied magnetic field of 120 mT provided by a commercial NdFeB permanent magnet. 39
Supplementary Fig. 44 | Flowchart of the physics-driven two-stage training protocol. The magnetic field functions as a physical tunable weight controller to modulate feature transmission. 40
Supplementary Note 5: Computational framework of the magnetically-driven in-sensor computing system 41
Supplementary Fig. 45 | Quantitative elemental analysis of oxygen-implanted *p*-FGaT validating the theoretical model. 43

Supplementary References..44

Supplementary Note 1: Inductively coupled plasma (ICP) implantation details

The oxygen plasma implantation equipment adopts ICP generation. The complete system includes a vacuum chamber, an RF plasma generator (equipped with a 13.56 MHz solid-state RF power supply), a vacuum pump, and an air inlet and exhaust port, and the treatment chamber is a cylindrical quartz glass. In the ICP configuration, an alternating current flowing through the inductive coil generates a time-varying electromagnetic field, which induces an electric field inside the chamber. This field accelerates free electrons, leading to repeated collisions with oxygen molecules and efficient ionization, thereby sustaining a high-density plasma. Compared to capacitively coupled plasma (CCP), ICP enables the generation of high plasma density at relatively low ion energies, minimizing ion-induced damage to the sample. This characteristic is particularly advantageous for processing ultrathin layered materials while preserving their structural integrity. The process consists of three consecutive steps: chamber evacuation, filling with oxygen, ionizing the oxygen and processing the sample (processing time in this study: 10-300 s).

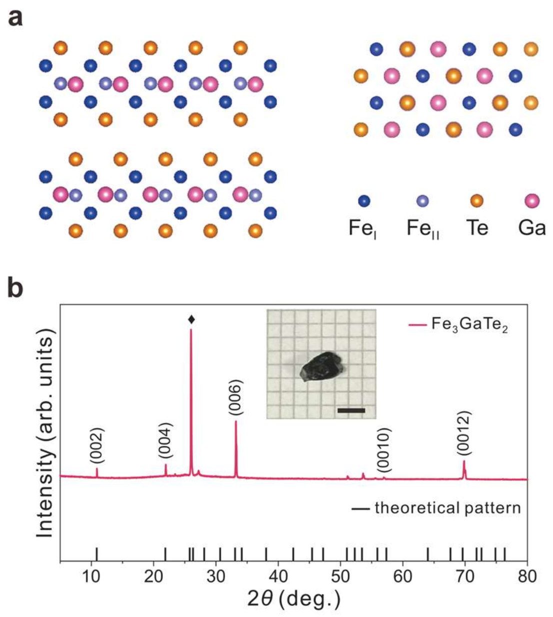

Supplementary Fig. 1 | Crystal characterization of the van der Waals layered FGaT single crystals. a Front (left) and top view (right) of the crystal structure of FGaT. **b** Experimental and theoretical X-ray diffraction (XRD) patterns of the FGaT bulk crystal. Inset shows an optical image of a typical bulk crystal. Scale bar, 2 mm in **b**.

FGaT crystallizes in a hexagonal structure with space group P6$_3$/mmc ($\alpha = \beta = 90°$, $\gamma = 120°$), consisting of layered blocks stacked along the *c*-axis, with an interlayer spacing of approximately 0.8 nm. Each block comprises two layers of Te atoms and one Fe_3Ga flake, where two distinct Fe atomic sites (Fe_I and Fe_{II}) are located between the two Te layers. The XRD pattern of the as-grown FGaT bulk crystal shows not only the characteristic diffraction peak of (00*l*), but also an additional peak at 26° (labeled with ♦), which is due to the (111) diffraction of Ga_2Te_3 (JCPDS No. 00-057-0364) reported by Hu *et al.* (*Adv. Mater.* **36**, 2403154 (2024)). The inclusion of this phase does not influence the exfoliation efficiency and purity of FGaT nanoflakes.

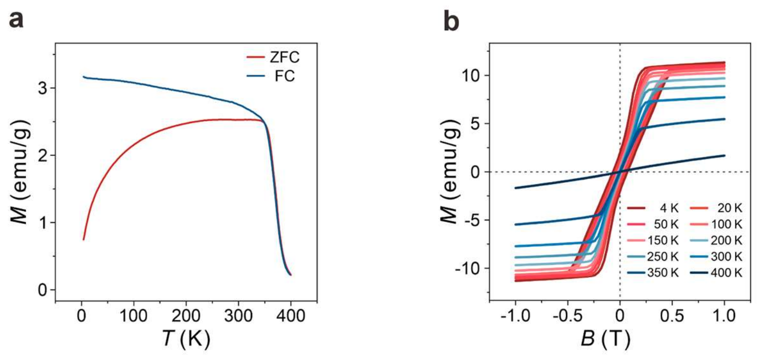

Supplementary Fig. 2 | Temperature-dependent magnetization measurements of FGaT bulk crystals using superconducting quantum interference (SQUID) device. a Zero-field-cooled and field-cooled (ZFC-FC) curves (out-of-plane field of 100 mT). **b** Temperature-dependent magnetization curves sweeping at out-of-plane fields.

ZFC-FC tests show a clear bifurcation in the low-temperature region, and the magnetism gradually weakens with the increase of the temperature, which is in accordance with the transition characteristics of ferromagnetic substances and confirms the room-temperature ferromagnetism of the prepared FGaT single crystals. The Curie temperatures (T_C) estimated by the maximum value of dM/dT is about 368 K, which is consistent with the previous reports. Supplementary Fig. 2b presents temperature-variable magnetization curves of the FGaT single crystal under the out-of-plane magnetic fields, which show a relatively large coercivity (B_s) of 70.8 mT at 3 K and a small B_s of 1.5 mT at 300 K. The magnetization fades between 350 and 400 K, in line with the T_C values determined in Supplementary Fig. 2a.

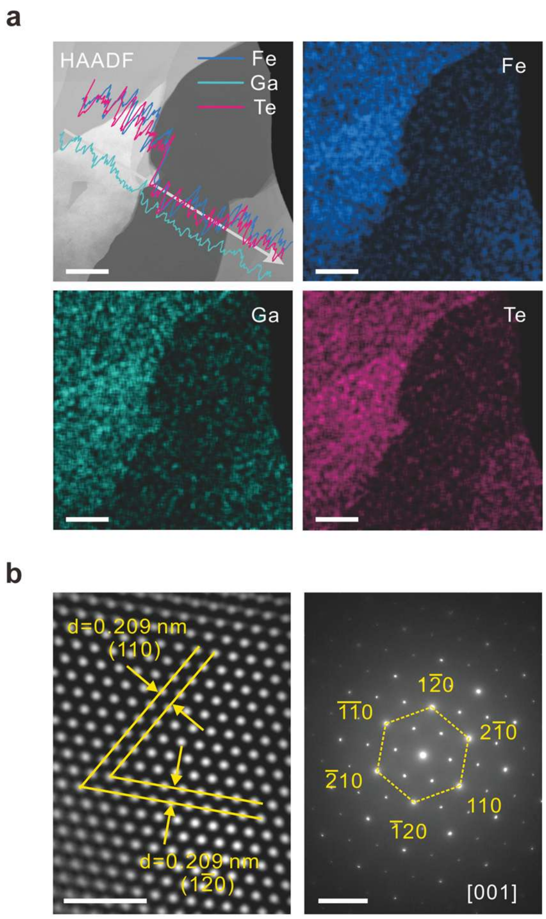

c

EDS-1

Element	Atomic Fraction (%)
Fe	49.79
Ga	17.99
Te	32.22

EDS-2

Element	Atomic Fraction (%)
Fe	51.24
Ga	16.04
Te	32.72

EDS-3

Element	Atomic Fraction (%)
Fe	50.09
Ga	16.81
Te	33.10

Supplementary Fig. 3 | Transmission electron microscopy (TEM) characterization of a pristine FGaT nanoflake. a Dark-field image and corresponding element mapping images. **b** High-resolution transmission electron microscope (HRTEM) image and corresponding selected area electron diffraction (SAED) pattern. **c** Energy dispersive X-ray spectroscopy (EDS) measurements of three random regions. Scale bars, 2 μm in all panels of **a**, 1 nm in the left panel of **b** and 5 nm^{-1} in the right panel of **b**.

EDS elemental mapping reveals a homogeneous spatial distribution of Fe, Ga, and Te throughout the sample. The measured atomic ratio of Fe:Ga:Te (approximating 3:1:2) closely aligns with the expected stoichiometric composition. High-resolution transmission electron microscopy analysis enables clear identification of the (110) and ($1\bar{2}0$) lattice planes, both exhibiting the same interplanar spacing of ~0.209 nm due to six-fold rotational symmetry, consistent with the theoretical lattice parameters of the material. Meanwhile, the SAED analysis further confirms the single-crystal quality of the material, showing characteristic two-fold hexagonal diffraction spots.

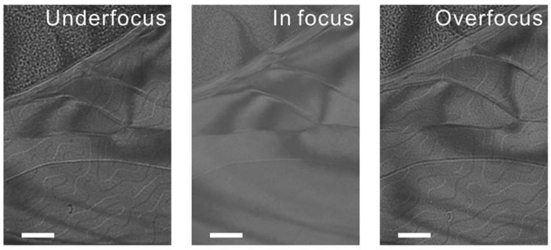

Supplementary Fig. 4 | Lorentz transmission electron microscopy (LTEM) characterization of a pristine FGaT nanoflake. LTEM images acquired under underfocus, in-focus, and overfocus conditions. Striped magnetic domain patterns are observed at room temperature, consistent with strong magnetic anisotropy. Scale bars, 500 nm in all panels.

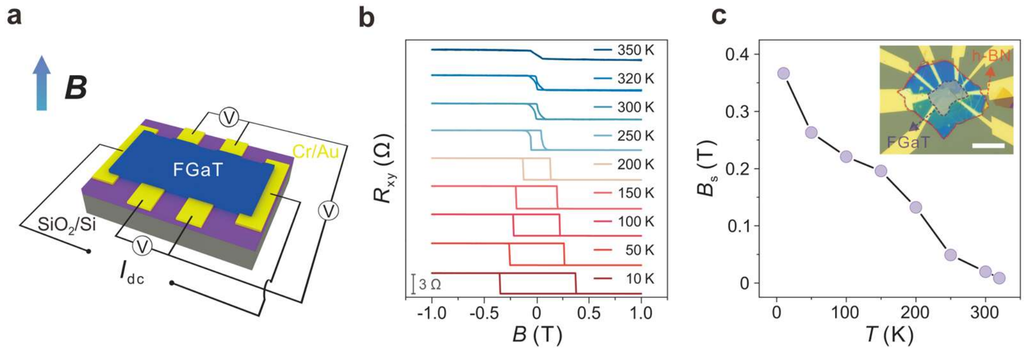

Supplementary Fig. 5 | Anomalous Hall measurements of a pristine FGaT nanoflake. a Schematic of the Hall device. **b** Temperature-dependent Hall resistance (R_{xy}). **c** Extracted saturation field (B_s) as a function of temperature. Inset: optical micrograph of the device (~70 nm thick) encapsulated by hexagonal boron nitride (*h*-BN). Scale bar, 40 μm in the inset of **c**.

Square-shaped hysteresis loops are observed over a wide temperature range, indicating robust ferromagnetic ordering (Supplementary Fig. 5b). The saturation field decreases with increasing temperature and vanishes above the T_C (Supplementary Fig. 5c).

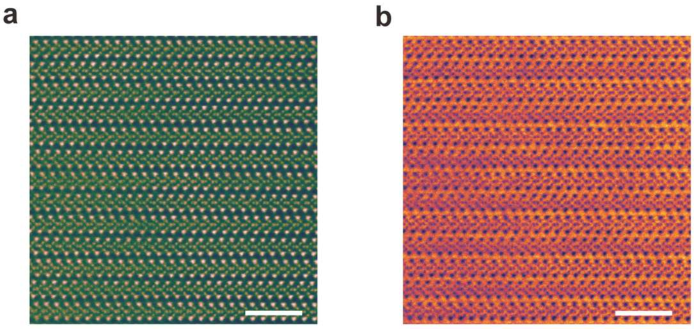

Supplementary Fig. 6 | Cross-sectional TEM images of *i*-FGaT. a Bright-field and **b** dark-field images. Fe_3Ga layers are sandwiched between Te terminations and separated by van der Waals gaps. Scale bars, 2 nm in **a** and **b**.

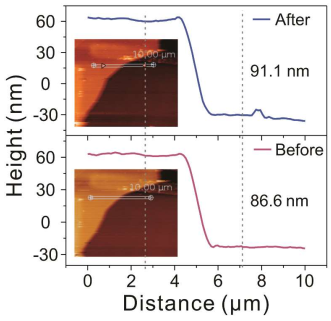

Supplementary Fig. 7 | Atomic force microscopy (AFM) characterization of FGaT nanoflakes before and after oxygen plasma implantation.

AFM images and corresponding height profiles show a thickness variation of ~5% after plasma treatment. The minimal change in thickness suggests that oxygen incorporation does not lead to significant interlayer expansion, indicating that oxygen is primarily incorporated within the lattice rather than as intercalated O_2 species (*Nat. Commun.* **11**, 5960 (2020)).

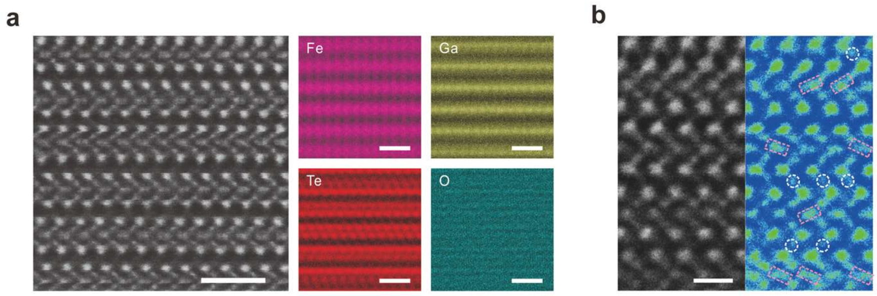

Supplementary Fig. 8 | High-resolution STEM characterization of *p*-FGaT. a Cross-sectional structural observation with corresponding EDS elemental mapping. **b** Enlarged TEM image (left) and corresponding dark-field image (right). Dashed pink boxes highlight intralayer oxygen-induced lattice distortions, while white circles denote interlayer oxygen interstitials. Scale bars, 1 nm in all panels of **a** and 0.5 nm in the left panel of **b**, respectively.

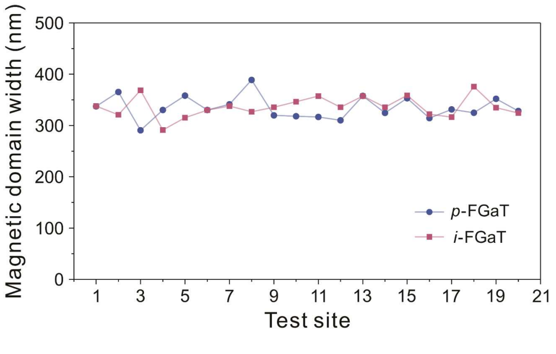

Supplementary Fig. 9 | Magnetic domain width statistics of *i*-FGaT and *p*-FGaT. Magnetic domain widths were statistically analyzed from 20 randomly selected regions in both *i*-FGaT and *p*-FGaT.

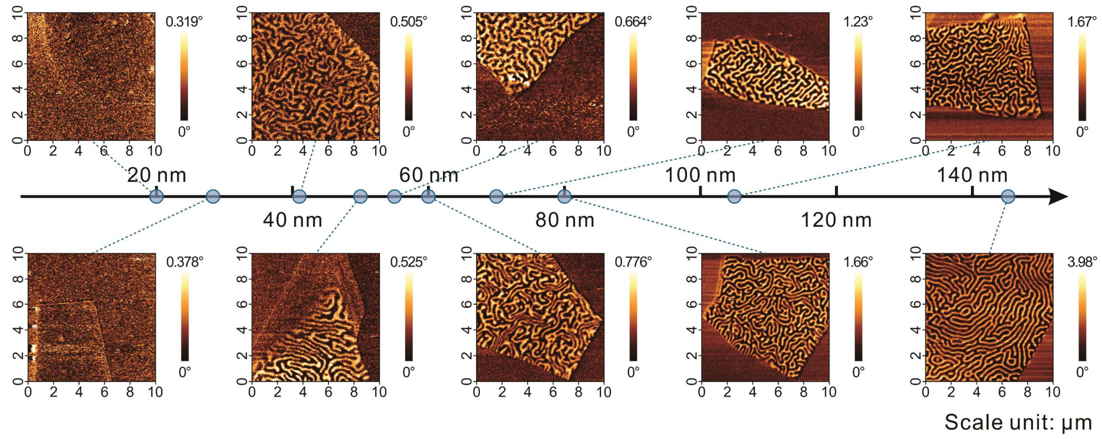

Supplementary Fig. 10 | Thickness-dependent magnetic domain structures of *p*-FGaT nanoflakes.

Magnetic domains of *p*-FGaT nanoflakes with varying thicknesses, characterized by magnetic force microscopy (MFM) at 300 K under zero external magnetic field. Thicker flakes (>30 nm) exhibit labyrinthine domain patterns, whereas thinner flakes (<30 nm) display a uniform single-domain state.

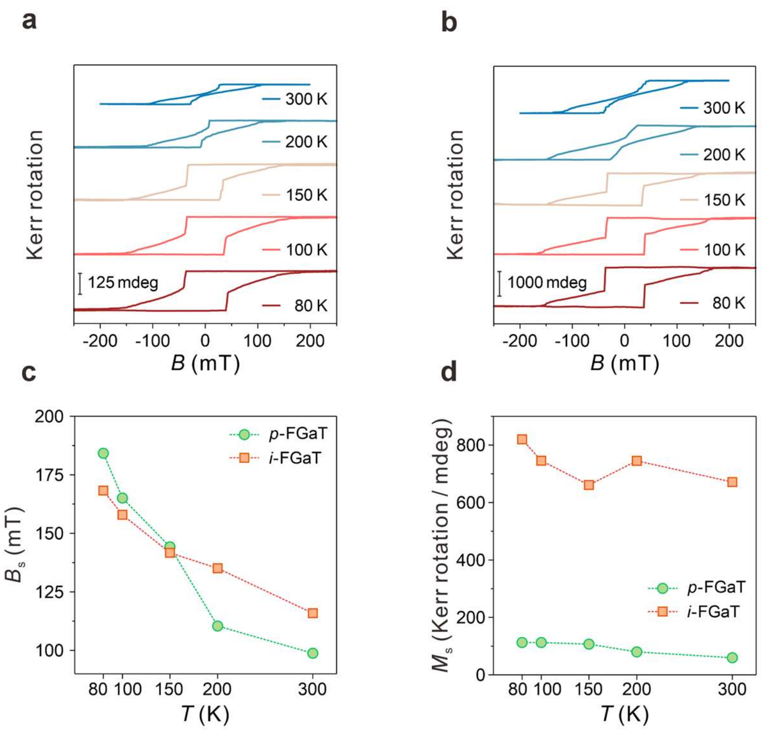

Supplementary Fig. 11 | Temperature-dependent magneto-optical Kerr effect (MOKE) signal comparison. a, b Hysteresis curves for *p*-FGaT (**a**) and *i*-FGaT (**b**). **c, d** Extracted B_s and M_s plots versus temperature.

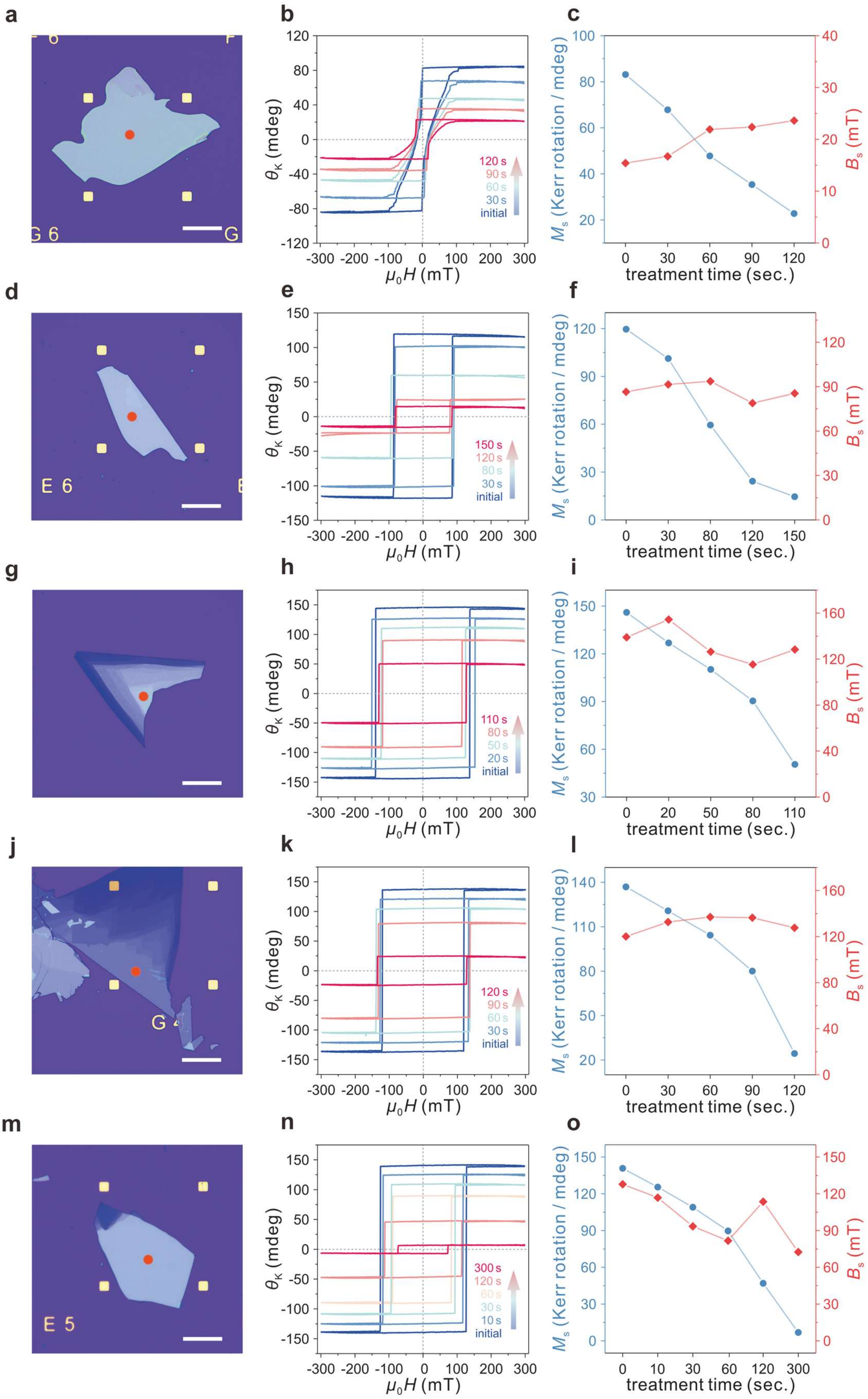
a
b
c
d
e
f
g
h
i
j
k
l
m
n
o
G 6
E 6
E 5
G
θ_K (mdeg)
μ_0H (mT)
M_s (Kerr rotation / mdeg)
B_s (mT)
treatment time (sec.)
120 s
90 s
60 s
30 s
initial
150 s
120 s
80 s
30 s
initial
110 s
80 s
50 s
20 s
initial
120 s
90 s
60 s
30 s
initial
300 s
120 s
60 s
30 s
10 s
initial

Supplementary Fig. 12 | Magnetic evolution of *p*-FGaT under cumulative oxygen plasma treatment. In situ MOKE characterization of five independent *p*-FGaT nanoflakes: **a-c** Sample 1; **d-f** Sample 2; **g-i** Sample 3; **j-l** Sample 4; **m-o** Sample 5. Left panels: optical micrographs with marked laser probing positions. Middle panels: evolution of magnetic hysteresis loops with increasing plasma treatment time. Right panels: extracted M_s and B_s as a function of treatment time. Scale bars, 20 μm for the optical micrographs in **a**, **d**, **g**, **j** and **m**.

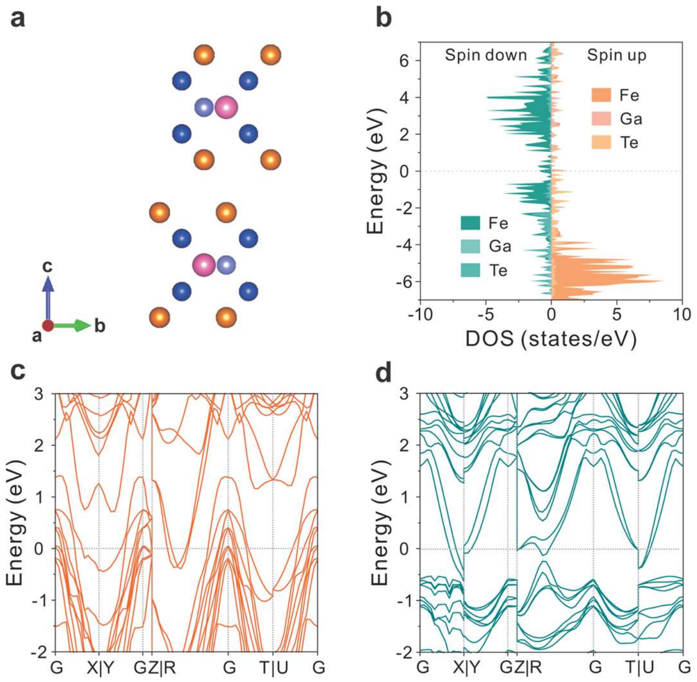

Supplementary Fig. 13 | Density functional theory (DFT) calculations of intrinsic *i*-FGaT. a Atomic structure model of the *i*-FGaT. **b** Calculated spin-polarized density of states (DOS), revealing a significant population of states at the Fermi level. **c, d** Electronic band structures for the spin-majority (**c**) and spin-minority (**d**) channels. The bands clearly cross the Fermi level (E_F = 0 eV) in both channels, confirming the metallic nature of the intrinsic phase, which contrasts with the semiconducting behavior of *p*-FGaT.

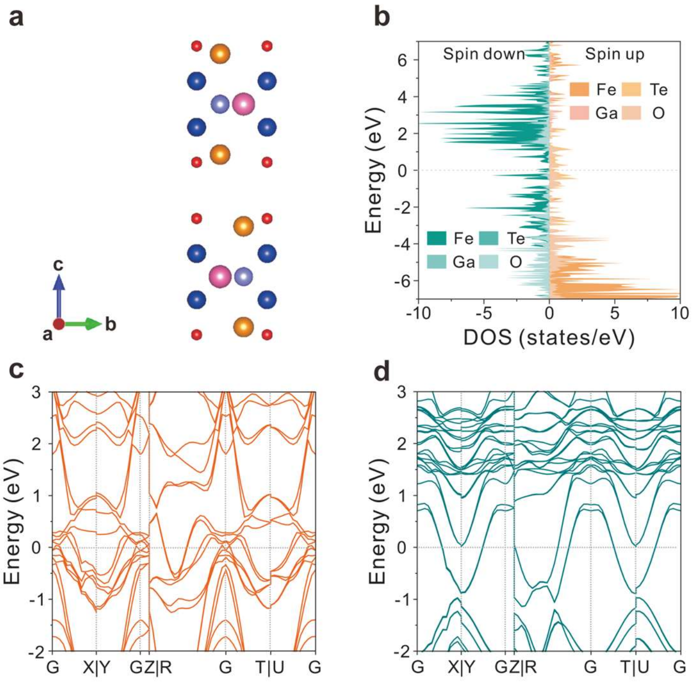

Supplementary Fig. 14 | DFT calculations of interlayer-oxygen-implanted FGaT. a Atomic structure model of FGaT with oxygen atoms situated in the interlayer space. **b** Calculated spin-polarized DOS, exhibiting a finite density of states at the Fermi level similar to the intrinsic phase. **c, d** Electronic band structures for the spin-majority (**c**) and spin-minority (**d**) channels. The bands clearly cross the Fermi level (E_F = 0 eV) in both channels, confirming that oxygen implantation in the interlayer region retains the metallic nature, failing to induce the metal-to-semiconductor transition observed in the experimentally realized *p*-FGaT.

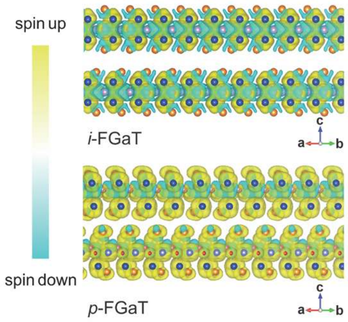

Supplementary Fig. 15 | Spin density distributions of FGaT before and after intralayer oxygen implantation. Comparison of spin density distributions in *i*-FGaT and *p*-FGaT, showing enhanced out-of-plane spin polarization in *p*-FGaT.

Supplementary Table 1 | Calculated elemental magnetic moments (unit: μ_B) of intralayer-oxygen-implanted FGaT.

Elements	*s*	*p*	*d*	Tot.
Te1	0.008	0.023	0.001	0.032
Te2	0.006	0.019	0.001	0.026
Te3	0.008	0.022	0.001	0.031
Te4	0.005	0.016	0.001	0.023
Fe1	0.041	0.040	3.555	3.636
Fe2	0.030	0.022	3.352	3.404
Fe3	0.042	0.038	3.550	3.630
Fe4	0.029	0.021	3.338	3.388
Fe5	0.031	0.019	3.454	3.505
Fe6	0.031	0.021	3.422	3.474
Ga1	0.040	-0.097	0.006	-0.051
Ga2	0.036	-0.102	0.005	-0.061
O1	0.018	0.143	0.000	0.161
O2	0.018	0.139	0.000	0.156

Supplementary Table 2 | Calculated elemental magnetic moments (unit: μ_B) of *i*-FGaT.

Elements	*s*	*p*	*d*	Tot.
Te1	0.006	-0.092	-0.002	-0.088
Te2	0.006	-0.092	-0.002	-0.088
Te3	0.006	-0.092	-0.002	-0.088
Te4	0.006	-0.092	-0.002	-0.088
Fe1	0.017	0.021	2.972	3.011
Fe2	0.017	0.021	2.972	3.011
Fe3	0.017	0.021	2.972	3.011
Fe4	0.017	0.021	2.972	3.011
Fe5	0.004	-0.011	2.677	2.671
Fe6	0.004	-0.011	2.677	2.671
Ga1	-0.035	-0.178	0.007	-0.207
Ga2	-0.035	-0.178	0.007	-0.207

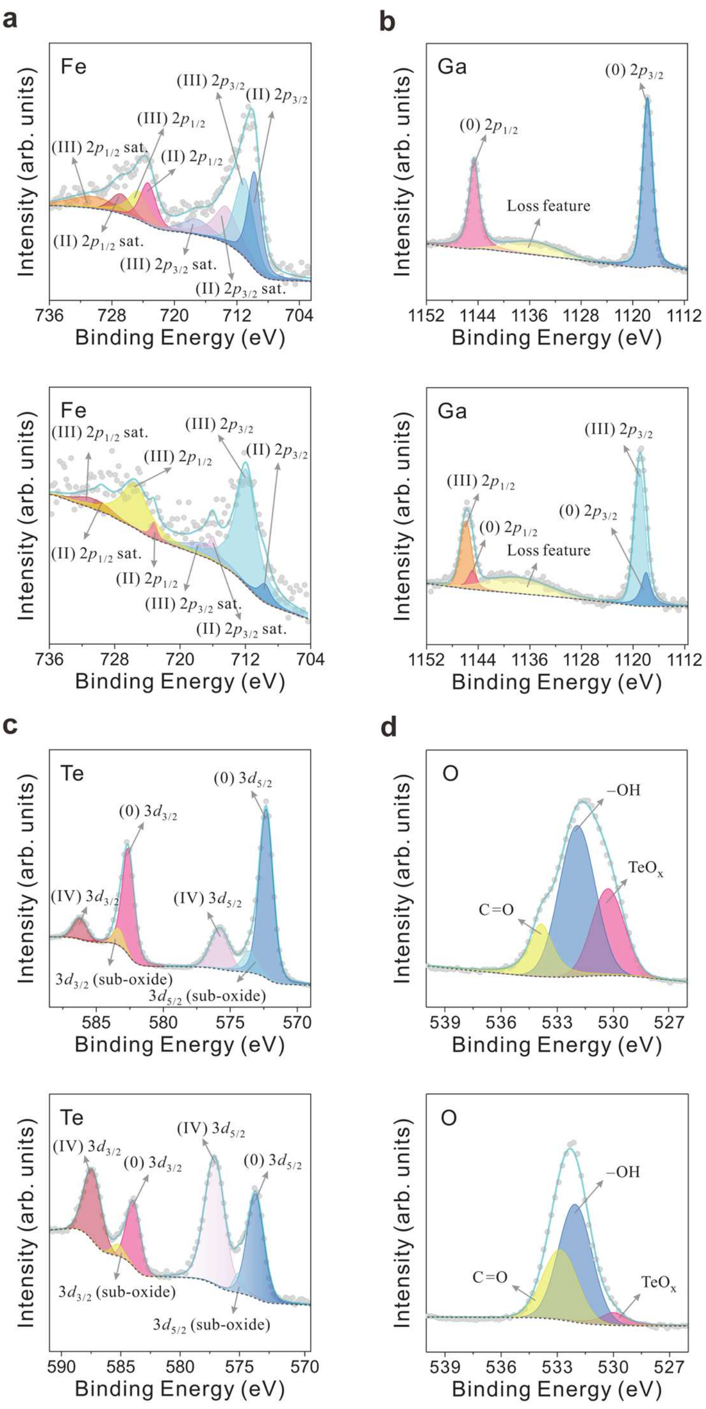

Supplementary Fig. 16 | X-ray photoelectron spectroscopy (XPS) characterization of FGaT nanoflakes. Top and bottom panels correspond to spectra before and after oxygen plasma implantation, respectively. **a** Fe-2*p*, **b** Ga-2*p*, **c** Te-3*d*, and **d** O-1*s*.

The valence states and chemical environment of Fe, Ga, Te and O before and after oxygen plasma implantation were analyzed by XPS. The relative proportions of Fe^{3+}, Ga^{3+}, and Te^{4+} are significantly enhanced compared to Fe^{2+}, Ga^{0}, and Te^{0} after implantation, which are closely correlated with electron-withdrawing capability of implanted oxygen species. The oxygen species located at ~530 eV refer to lattice oxygen in TeO_x due to surface mild oxidation of *i*-FGaT. Through plasma treatment, the surface TeO_x is mostly inhibited, indicating a potential oxidation resistance

ability of *p*-FGaT. The O-1*s* binding energies situated between 531 and 534 eV stem from adsorbed C=O and -OH species. Therefore, a higher oxidation state for Te element does not represent the formation of TeO_x layer, instead revealing the interaction between interstitial oxygen with Te layers. These spectral changes together demonstrate the formation of oxygen-related defects in the *p*-FGaT layer rather than conventional oxidation.

Supplementary Table 3 | Bader charges (*e*) of intralayer-oxygen-implanted FGaT, calculated using DFT.

Elements	QACF	ZVAL	Qbader
Te1	6.044955	6	-0.04
Te2	5.965409	6	0.03
Te3	5.971942	6	0.03
Te4	5.960389	6	0.04
Fe1	13.596955	14	0.40
Fe2	13.803714	14	0.20
Fe3	13.809427	14	0.19
Fe4	13.763144	14	0.24
Fe5	13.485362	14	0.51
Fe6	13.588619	14	0.41
Ga1	12.659533	13	0.34
Ga2	12.922609	13	0.08
O1	7.233103	6	-1.23
O2	7.194840	6	-1.19

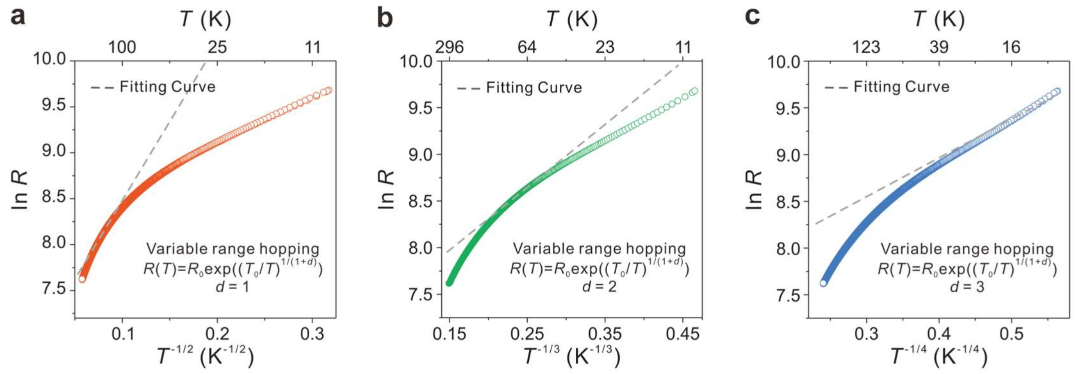

Supplementary Fig. 17 | Mott variable range hopping (VRH) fitting of the *p*-FGaT. Compared to three typical VRH models, three-dimensional disorder mechanism is the most ideal fit to describe the *p*-FGaT at low temperatures. The fitted dimensionality of d = 3 suggests that the observed transport behavior originates from the bulk effect.

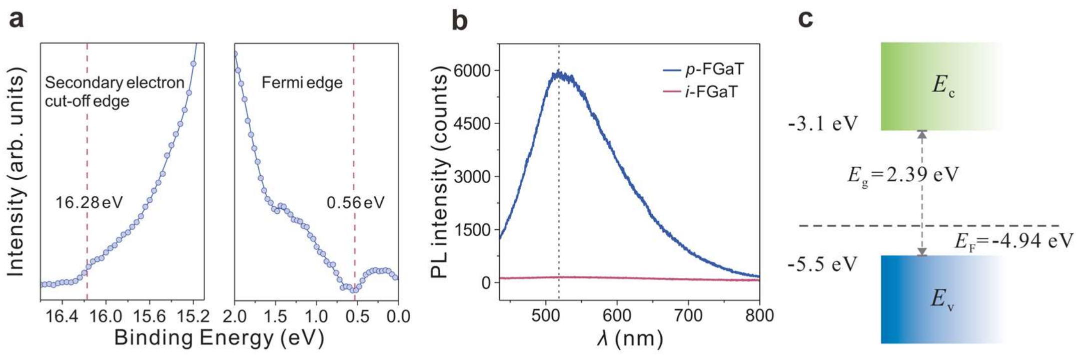

Supplementary Fig. 18 | Electronic structure characterization of *p*-FGaT. a Ultraviolet photoelectron spectroscopy (UPS) spectra near the secondary electron cut-off and Fermi edge. **b** Photoluminescence (PL) spectra of *i*-FGaT and *p*-FGaT. **c** Derived energy band diagram.

The Fermi level is estimated from the UPS cut-off energy, yielding $E_F \approx -4.94$ eV. Combined with the valence band offset, the valence band maximum (VBM) is determined to be ≈−5.5 eV. The optical bandgap (~2.39 eV) extracted from PL measurements gives a conduction band minimum (CBM) at ≈−3.1 eV.

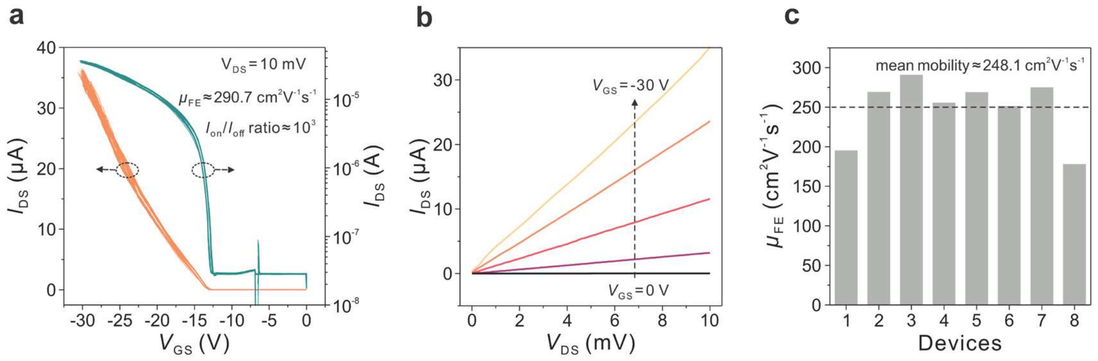

Supplementary Fig. 19 | Field-effect transistor (FET) performance evaluation. a Transfer characteristic curves (I_{DS}~V_{GS}) on linear and logarithmic scales. The measurements were conducted for 50 cycles and the source-drain voltage V_{DS} is fixed at 10 mV. **b** Output characteristic curves (I_{DS}~V_{DS}) by varying gating voltages V_{GS} from 0 V to −30 V. **c** Statistical distribution of field-effect mobilities (μ_{FE}) achieved from eight *p*-FGaT devices.

The negative gate voltages (V_{GS}) effectively modulates the source-drain current (I_{DS}) at a fixed source-drain voltage (V_{DS}) of 10 mV, indicating hole-dominated transport. The threshold voltage (V_{th}) is approximately −12.5 V, confirming that the device operates in enhancement mode. From the transfer characteristic curve (I_{DS}~V_{GS}), an on/off current ratio of 10^3 and a subthreshold slope of 310 mV dec^{-1} are observed. Considering that the output characteristic curves remain linear around V_{DS} = 10 mV, the linear field-effect mobility was calculated using Eq. (1):

$$\mu_{FE} = \frac{L}{WC_i V_{DS}} \frac{dI_{DS}}{dV_{GS}} \quad (1)$$

The obtained μ_{FE} is 290.7 cm^2 V^{-1}s^{-1}, using electrode width to length ratio (*W/L*) of 5 and a specific capacitance density (C_i) of 1.151×10^{-8} F cm^{-2}. The average mobilities across multiple devices are determined as 248.1 cm^2 V^{-1}s^{-1}.

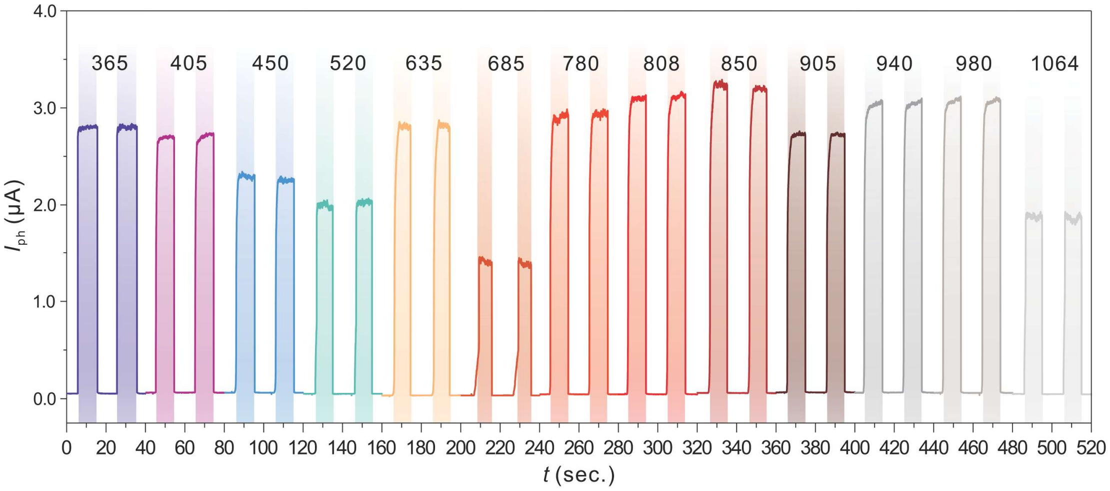

Supplementary Fig. 20 | Broadband photoresponse of *p*-FGaT sensor (365-1064 nm).

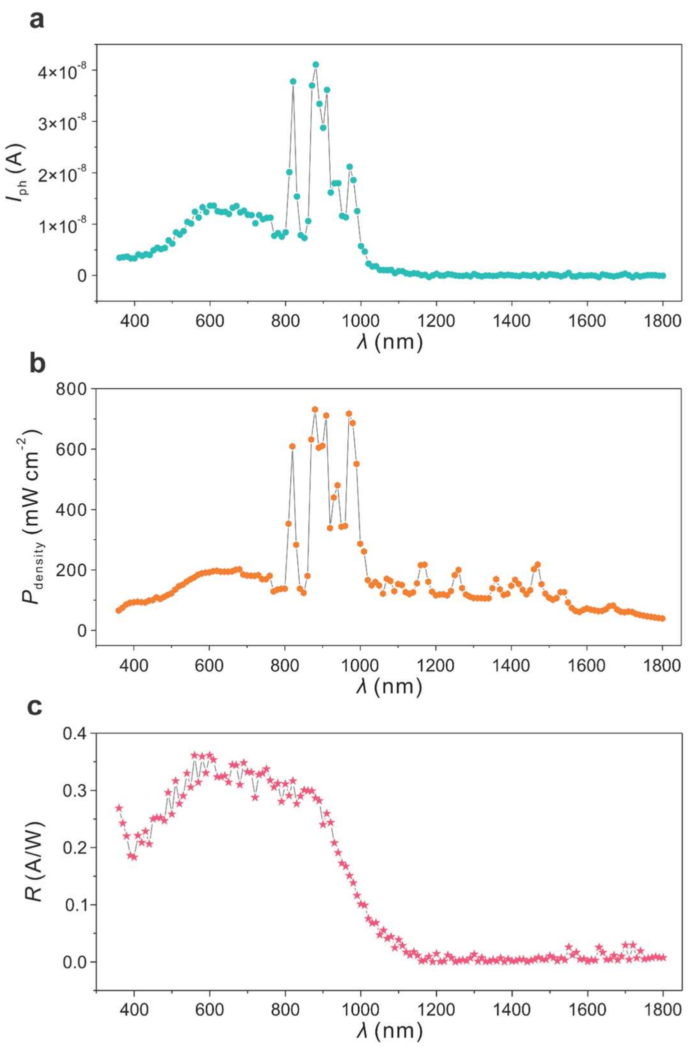

Supplementary Fig. 21 | Wavelength-dependent optoelectronic characteristics of the *p*-FGaT device. a Measured photocurrent (I_{ph}) spectrum ranging from 360 nm to 1800 nm. A distinct and measurable photoresponse is observed in the sub-bandgap region (λ > 520 nm), confirming the broadband detection capability. **b** Optical power density spectrum of the light source used for the measurement. The profile reflects the output characteristics of the excitation system. **c** Spectral responsivity (R) curve calculated from the data in **a** and **b**. The effective illumination area A_{eff} was determined to be ~19.63 μm^2 using a calibrated laser spot diameter of 5 μm.

Supplementary Note 2: Details on the calculation of responsivity (*R*)

To validate the wavelength-dependent responsivity, we provide the raw photocurrent spectrum, wavelength-dependent optical power density, and the derived responsivity curve in Supplementary Fig. 21. As clearly shown in Supplementary Fig. 21a, the device generates a substantial and measurable photocurrent not only in the visible range but also extending well into the near-infrared region ($\lambda > 520$ nm), which corresponds to photon energies below the intrinsic bandgap ($E_g \approx 2.39$ eV). This observation confirms that the sub-bandgap response reported in the main text is a real physical signal arising from defect-mediated transitions or internal polar fields, rather than a measurement artifact or noise.

To ensure the accuracy and reliability of the responsivity data presented in Fig. 3b and Supplementary Fig. 21c, we employed a rigorous calculation method based on precise calibration of the effective illuminated area. The spectral responsivity (*R*) was derived strictly from the measured photocurrent (I_{ph}) and optical power density ($P_{density}$) using Eq. (2):

$$R(\lambda) = \frac{I_{ph}(\lambda)}{P_{in}(\lambda)} = \frac{I_{ph}(\lambda)}{P_{density}(\lambda) \times A_{eff}} \quad (2)$$

where $I_{ph}(\lambda)$ is the measured photocurrent at each specific wavelength; $P_{density}(\lambda)$ is the wavelength-dependent optical power density of the excitation source (measured by a calibrated power meter, as shown in Supplementary Fig. 21b); A_{eff} is the effective photo-active area of the device.

In our experimental setup, the laser beam was focused to a spot diameter of 5 μm. Consequently, the effective area was calculated as $A_{eff} = \pi \times (2.5\ \mu m)^2 \approx 19.63\ \mu m^2$. We carefully ensured that the laser spot was fully confined within the device channel to maximize collection efficiency. This strict normalization process confirms that the observed spectral characteristics are intrinsic to the *p*-FGaT material.

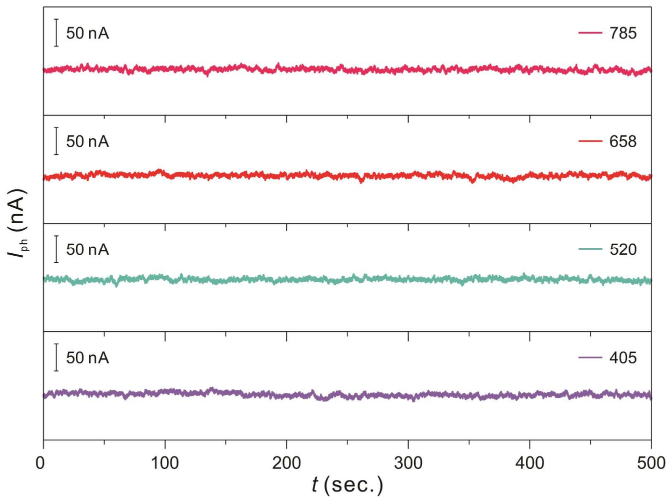

Supplementary Fig. 22 | Wavelength-dependent photocurrent response curves of *i*-FGaT device under 0.1 V bias. The incident light power densities are 570, 500, 415, and 460 mW mm^{-2} for 405, 520, 658, and 785 nm, respectively. The modulation frequencies (*f*) are uniformly set as 1 Hz. *i*-FGaT exhibits negligible photoresponse across all excitation wavelengths due to its metallic nature.

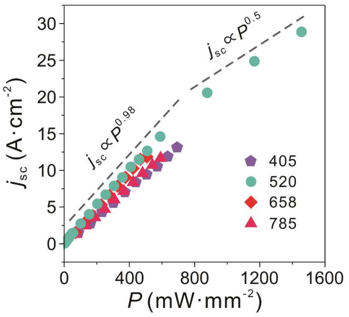

Supplementary Fig. 23 | Short-circuit current density (j_{sc}) versus optical power of the *p*-FGaT device illuminated by different light sources.

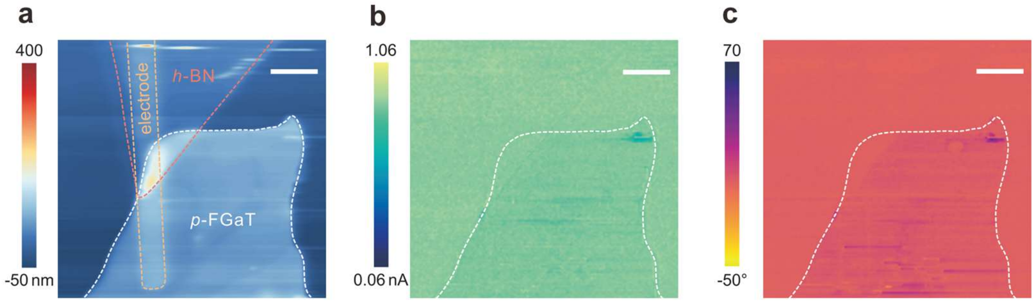

Supplementary Fig. 24 | Piezoresponse force microscopy (PFM) characterization of *p*-FGaT. **a** AFM topography, **b** PFM amplitude, and **c** PFM phase images of the *p*-FGaT nanoflake surface. Scale bars, 5 μm in **a**-**c**.

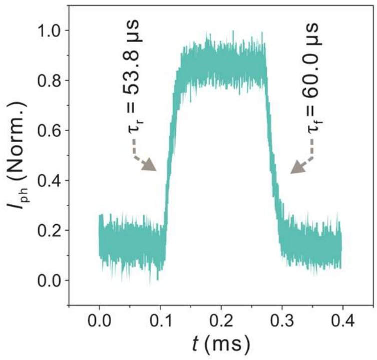

Supplementary Fig. 25 | Rise and fall dynamics of the short-circuit photocurrent under 520 nm illumination.

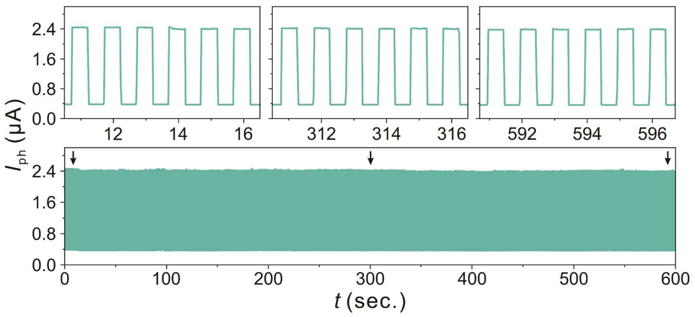

Supplementary Fig. 26 | Photocurrent response curves of *p*-FGaT device under 0.1 V bias conditions with modulated 520 nm excitation pulses (f = 1 Hz, P = 500 mW mm^{-2}).

Device #1:

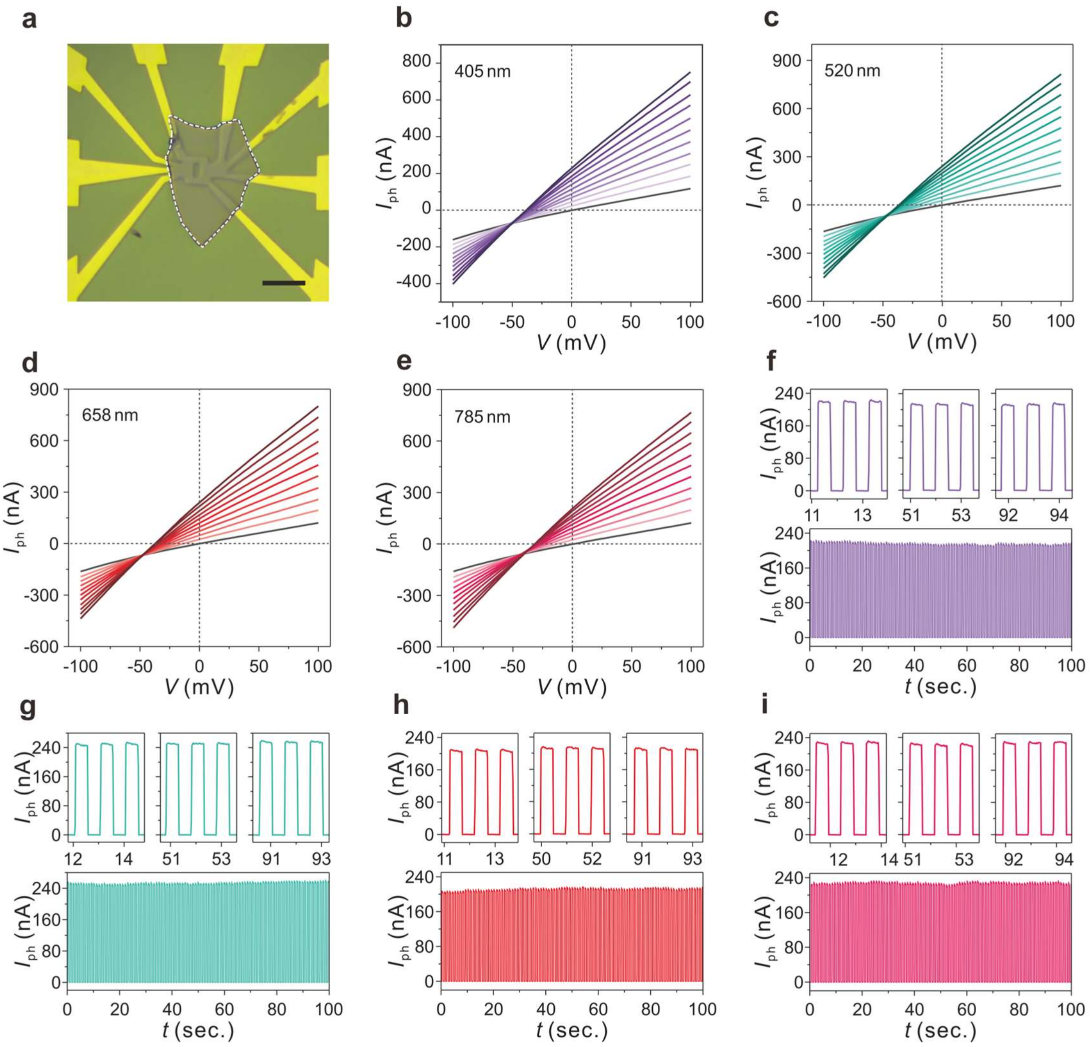

Supplementary Fig. 27 | Photovoltaic performance of *p*-FGaT Device #1 (~105 nm). a Optical micrograph of the fabricated device. **b-e** Current-voltage (*I*-*V*) curves measured under varying incident light power densities for excitation wavelengths of 405 nm (**b**), 520 nm (**c**), 658 nm (**d**), and 785 nm (**e**). The maximum optical power densities (P_{max}) are 570, 500, 415, and 460 mW mm^{-2}, respectively. **f-i** Time-resolved short-circuit photocurrent response curves recorded under self-driven conditions at the corresponding P_{max} for 405 nm (**f**), 520 nm (**g**), 658 nm (**h**), and 785 nm (**i**) laser illumination. The light switching frequency is fixed at 1 Hz. Scale bar, 20 μm in **a**.

Device #2:

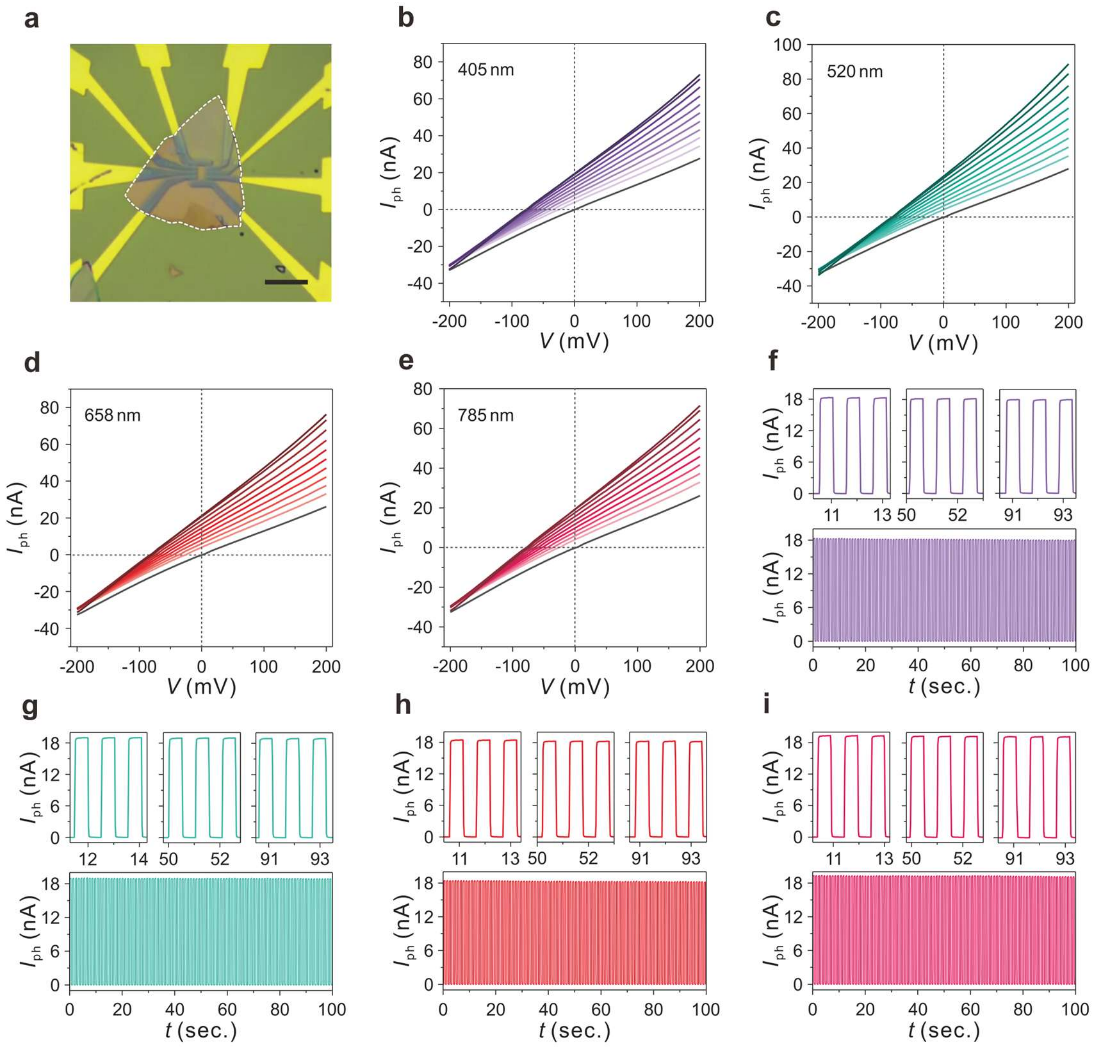

Supplementary Fig. 28 | Photovoltaic performance of *p*-FGaT Device #2 (~30 nm). a Optical micrograph of the device. **b-i** Corresponding power-dependent *I-V* curves (**b-e**) and time-resolved photocurrent responses (**f-i**) measured under illumination at 405, 520, 658, and 785 nm. The experimental conditions, including the maximum power densities and modulation frequency, are identical to those used for Device #1 (Supplementary Fig. 27). Scale bar, 20 μm in **a**.

Device #3:

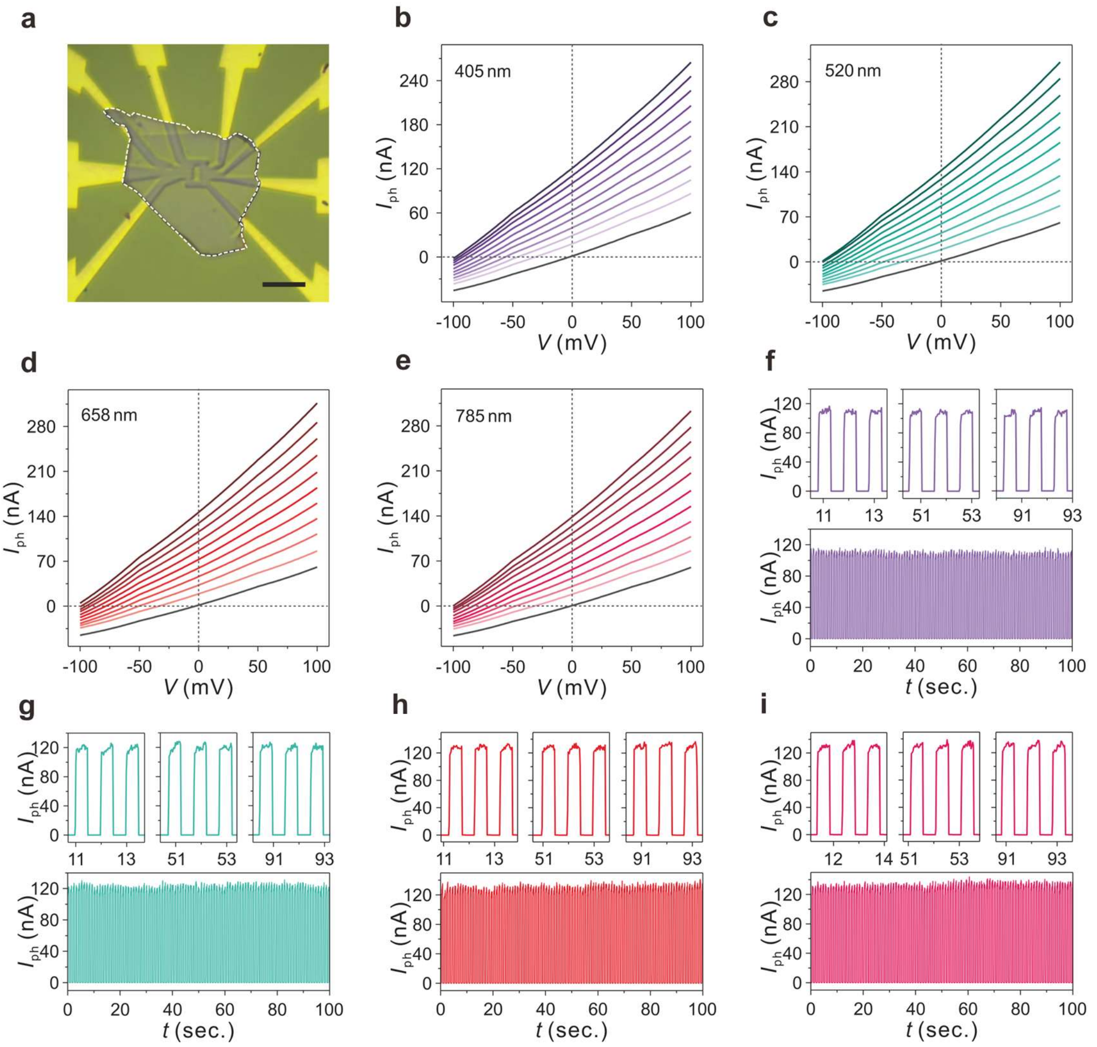

Supplementary Fig. 29 | Photovoltaic performance of *p*-FGaT Device #3 (~73 nm). a Optical micrograph of the device. **b-i** Comprehensive photoelectric characterization showing *I-V* curves (**b-e**) and periodic on/off switching responses (**f-i**) for the four indicated wavelengths. All testing parameters are consistent with those of Devices #1 and #2. Scale bar, 20 μm in **a**.

Measurements on multiple *p*-FGaT devices with varying thicknesses consistently reveal robust self-driven photovoltaic behavior. Despite differing thicknesses, all devices exhibit consistent current-voltage characteristics and time-resolved photoresponse. The high consistency across different samples strongly demonstrates that the observed BPV effect is an intrinsic property of oxygen plasma-engineered *p*-FGaT materials, rather than arising from device geometry or surface effects.

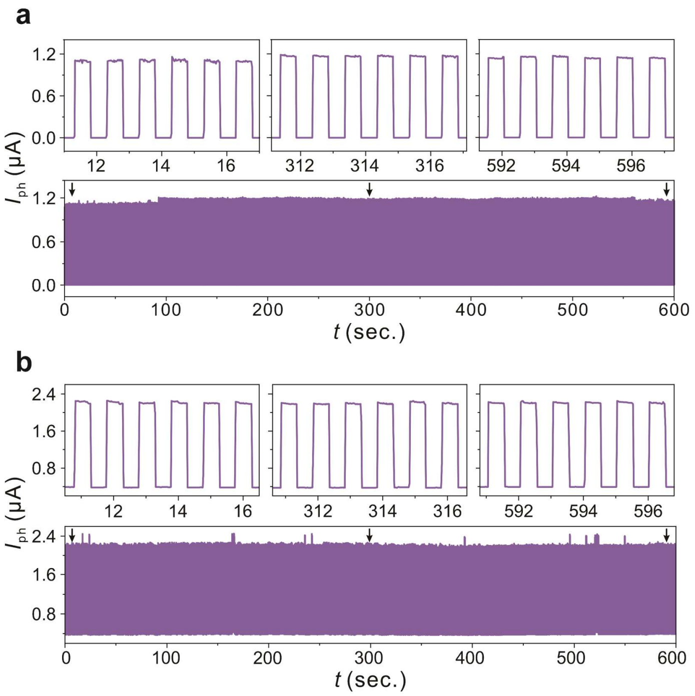

Supplementary Fig. 30 | Photocurrent response curves of *p*-FGaT device under self-driven and 0.1 V bias conditions with modulated 405 nm excitation pulses (f = 1 Hz, P = 570 mW mm^{-2}).

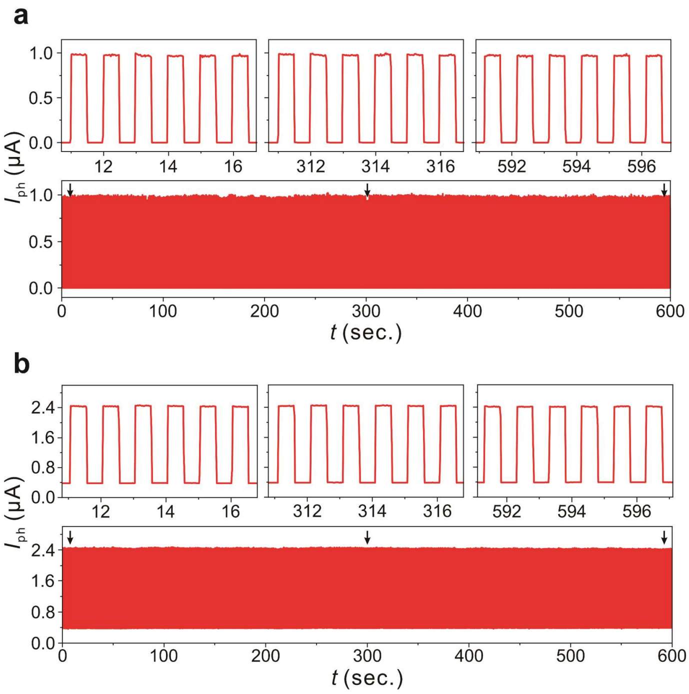

Supplementary Fig. 31 | Photocurrent response curves of *p*-FGaT device under self-driven and 0.1 V bias conditions with modulated 658 nm excitation pulses (*f* = 1 Hz, *P* = 415 mW mm^{-2}).

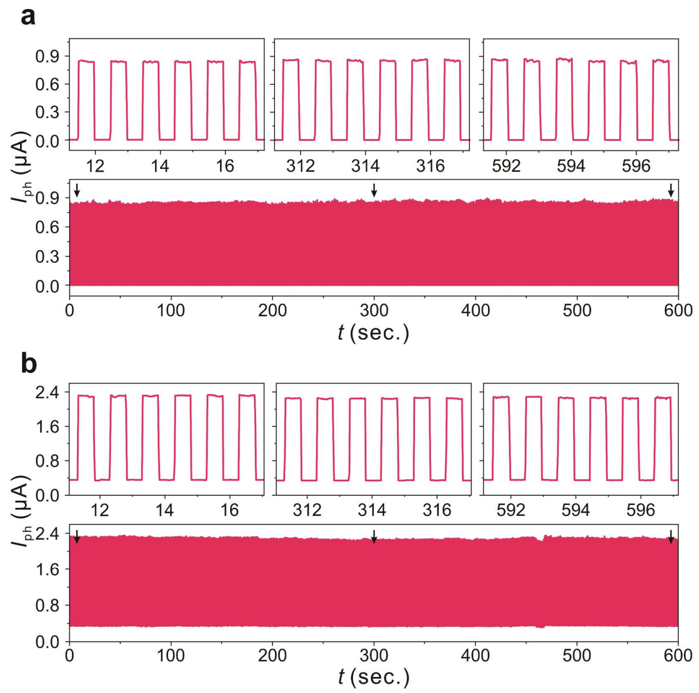

Supplementary Fig. 32 | Photocurrent response curves of *p*-FGaT device under self-driven and 0.1 V bias conditions with modulated 785 nm excitation pulses (*f* = 1 Hz, *P* = 460 mW mm^{-2}).

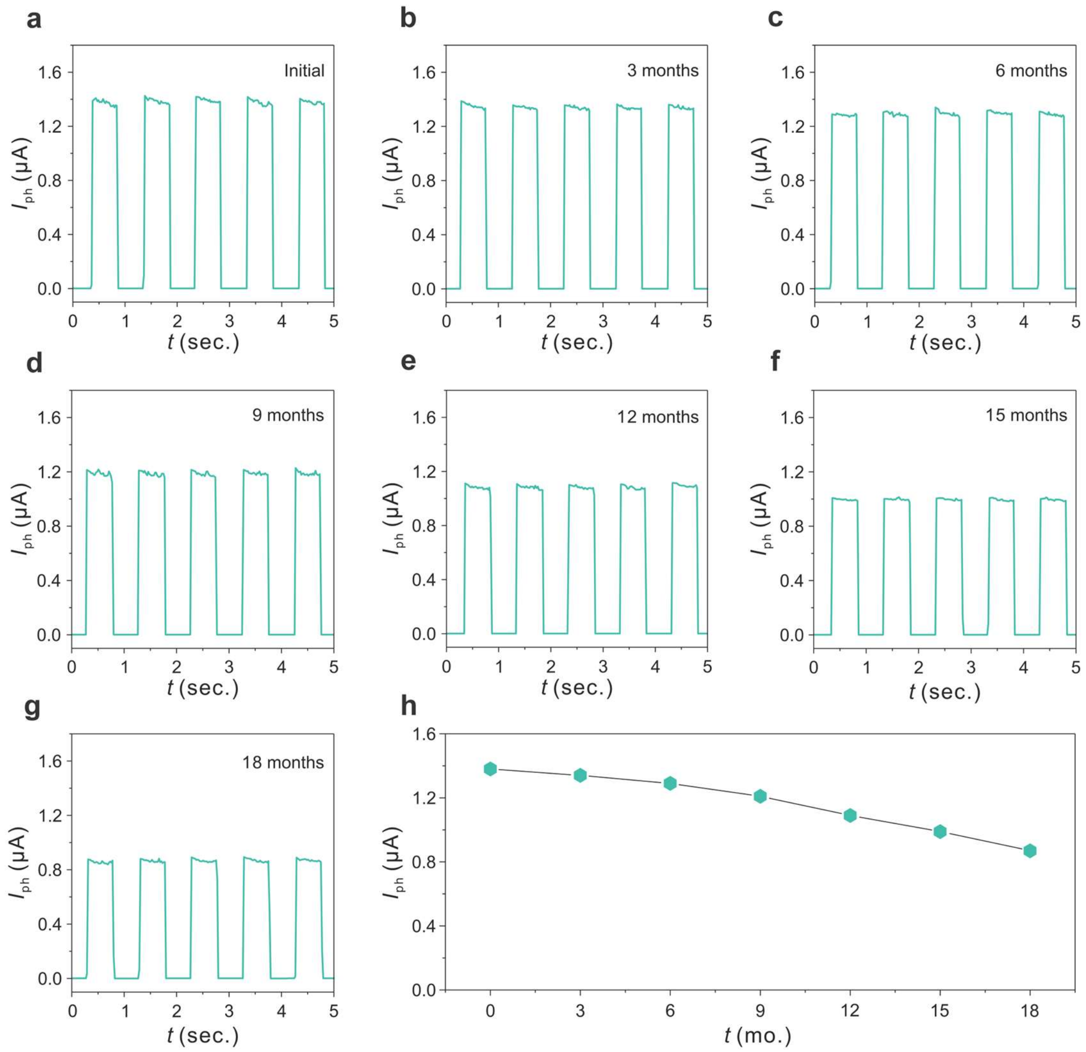

Supplementary Fig. 33 | Long-term environmental stability of the *p*-FGaT device. a-g Time-resolved short-circuit photocurrent response curves measured over an 18-month period. All measurements were conducted under self-driven conditions with 520 nm laser illumination at a power density of 500 mW mm^{-2}. The tests were performed at the following intervals: **a** Initial state, **b** 3 months, **c** 6 months, **d** 9 months, **e** 12 months, **f** 15 months, and **g** 18 months. The device was stored under ambient conditions between measurements. **h** Summary plot of the photocurrent as a function of storage time (months), extracted from the steady-state values in **a-g**.

Supplementary Table 4 | Performance comparison of characteristic response wavelengths and short-circuit photocurrent densities among previously reported BPV materials.

Materials	Photocurrent density ($A\cdot cm^{-2}$)	Light wavelength	Power ($mW\cdot mm^{-2}$)	Ref.
p-FGaT	1.51	405 nm	87.0	this work
p-FGaT	1.48	520 nm	54.0	this work
p-FGaT	1.53	658 nm	69.7	this work
p-FGaT	1.30	785 nm	70.4	this work
3*R*-WS_2	6.3×10^{-3}	632 nm	9.7×10^{-3}	1
Te	1.6×10^{-2}	1.31 μm	2.3×10^{-2}	2
MoS_2/BP	8.3×10^{-2}	633 nm	1.0	3
WSe_2/BP	6.4×10^{-2}	633 nm	1.5	3
MoS_2	0.1	638 nm	4.9	4
WS_2	0.2	638 nm	9.9	5
BFO	10.3	405 nm	397.5	6
TaAs	649.4	10 μm	2.0×10^{5}	7
$MoTe_2$	0.8	400 nm	98.7	8
$TaIrTe_4$	5.0	4 μm	8.0×10^{3}	9
CIPS	9.6×10^{-4}	405 nm	0.3	10
3*R*-MoS_2	10.1	630 nm	1.4×10^{4}	11
MPbI	1.0×10^{-4}	VIS	0.3	12
BFCO	1×10^{-3}	635 nm	15.0	13
KBNNO	4.4×10^{-8}	UV	4×10^{-3}	14
TTF-CA	1.1×10^{-6}	VIS	1.0	15
BTO	7.2×10^{-6}	360 nm	5.1	16
PZTO	1.1×10^{-8}	UV	0.3	16
PLZT	3.9×10^{-8}	UV	0.1	17
BBLT	7.0×10^{-9}	UV	0.1	18
OMPH	5.3×10^{-8}	450 nm	0.8	19

Abbreviations in Supplementary Tables 4 and 5: BFO, $BiFeO_3$; CIPS, $CuInP_2S_6$; MPbI, methylammonium lead iodide; BFCO, Bi_2FeCrO_6; KBNNO, $[KNbO_3]_{1-x}[BaNi_{1/2}Nb_{1/2}O_{3-\delta}]_x$; TTF-CA, tetrathiafulvalene-*p*-chloranil; BTO, $BaTiO_3$; PZTO, $Pb(Zr,Ti)O_3$; PLZT, lead lanthanum zirconate titanate; BBLT, $Ba_{0.875}(Bi_{0.5}Li_{0.5})_{0.125}TiO_3$; OMPH, organometallic perovskite-type halide; KBFO, $KBiFe_2O_5$; BFO:Mn, Mn-doped $BiFeO_3$; PZT, $Pb(Zr,Ti)O_3$; ZnO:Sb, Sb-doped ZnO; KNO:Fe, Fe-doped $KNbO_3$; LNO:Fe, Fe-doped $LiNbO_3$; BGO, $Bi_{12}GeO_{20}$; PTO, $PbTiO_3$.

Supplementary Table 5 | Comparison of the BPV coefficients of *p*-FGaT with previously reported materials.

Materials	Category	BPV Coefficient β (V^{-1})	Ref.
p-FGaT	Magnetic Semiconductor	0.25	this work
WS_2	TMD	~0.27	20
MoS_2	TMD	~0.075	4
BFCO	Multiferroic	~0.63	21
TaAs	Weyl Semimetal	~0.075	7
KBFO	Multiferroic	$\sim 3.7*10^{-3}$	22
BFO (bulk)	Ferroelectric	$\sim 3.7*10^{-4}$	23
BFO:Mn	Ferroelectric	$\sim 2.4*10^{-4}$	24
BFO (film)	Ferroelectric	$\sim 1.1*10^{-4}$	24
GaP	Semiconductor	$\sim 1.4*10^{-5}$	25
PZT	Ferroelectric	$\sim 1.1*10^{-5}$	17
BTO	Ferroelectric	$\sim 3.0*10^{-6}$	26
ZnO	Semiconductor	$\sim 1.9*10^{-6}$	27
ZnO:Sb	Semiconductor	$\sim 8.0*10^{-7}$	28
Te	Semiconductor	$\sim 3.2*10^{-7}$	29
ZnS	Semiconductor	$\sim 2.1*10^{-7}$	29
KNO:Fe	Ferroelectric	$\sim 6.0*10^{-8}$	29
SbSI	Ferroelectric	$\sim 5.8*10^{-8}$	30
GaAs	Semiconductor	$\sim 2.0*10^{-8}$	29
LNO:Fe	Ferroelectric	$\sim 1.0*10^{-8}$	31
BGO	Piezoelectric	$\sim 1.0*10^{-9}$	32
PTO	Ferroelectric	$\sim 5.6*10^{-10}$	29

Supplementary Table 5 shows that the *p*-FGaT device achieves a coefficient as high as 0.25 V^{-1}, several orders of magnitude higher than those of conventional bulk ferroelectrics (typically 10^{-4}-10^{-5} V^{-1}) and comparable to state-of-the-art low-dimensional systems such as WS_2 nanotubes. This performance originates from the intrinsic properties of *p*-FGaT as a magnetic semiconductor: unlike wide-bandgap oxides, it combines strong visible light absorption with significant inversion symmetry breaking caused by oxygen plasma implantation, enabling efficient BPV generation.

Supplementary Note 3: Preparation details of thermally-oxidized FGaT (*o*-FGaT)

The device fabrication process is identical to that in the main text, except that it is thermally annealed under ambient atmospheric conditions. Specifically, the fabricated devices were transferred to a hot plate preheated to 200°C, heated isothermally for 180 minutes, and then naturally cooled to room temperature. By systematically employing this heat treatment protocol, a comparison with similar devices treated with oxygen plasma was established, allowing for an assessment of the influence of *o*-FGaT on device performance.

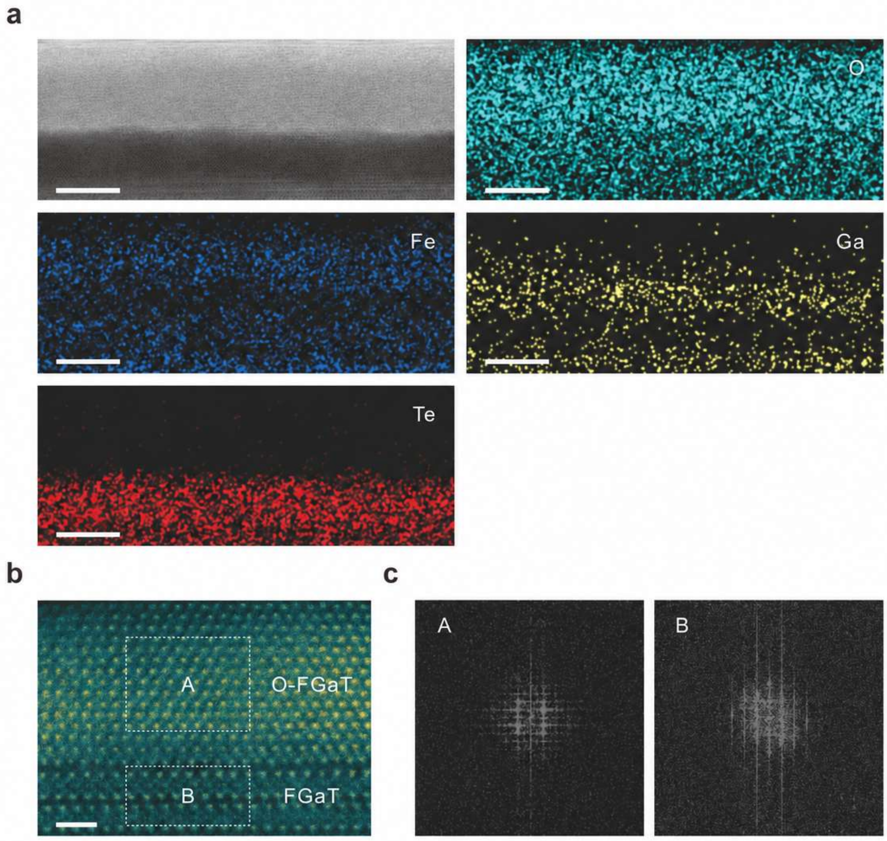

Supplementary Fig. 34 | Structural characterization of thermally-oxidized FGaT (*o*-FGaT). a Cross-sectional STEM image and corresponding EDS elemental mapping. **b** High-resolution TEM image. **c** Fast Fourier transform (FFT) patterns corresponding to dashed box regions in **b**. Scale bars, 5 nm in all panels of **a** and 1 nm in **b**.

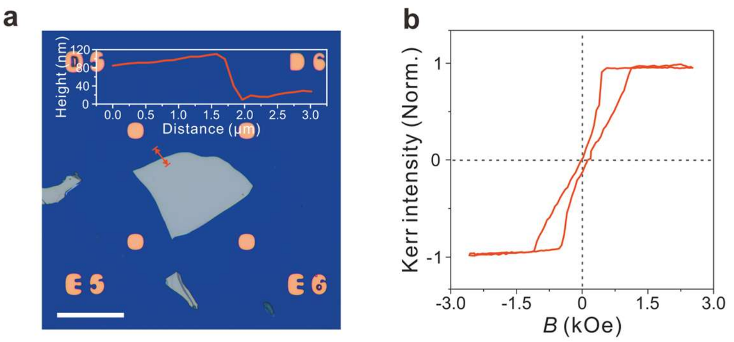

Supplementary Fig. 35 | Morphology and magnetic characterization of *o*-FGaT. a Optical micrograph and corresponding AFM height profile. **b** Normalized MOKE hysteresis loop. Scale bar, 30 μm in **a**.

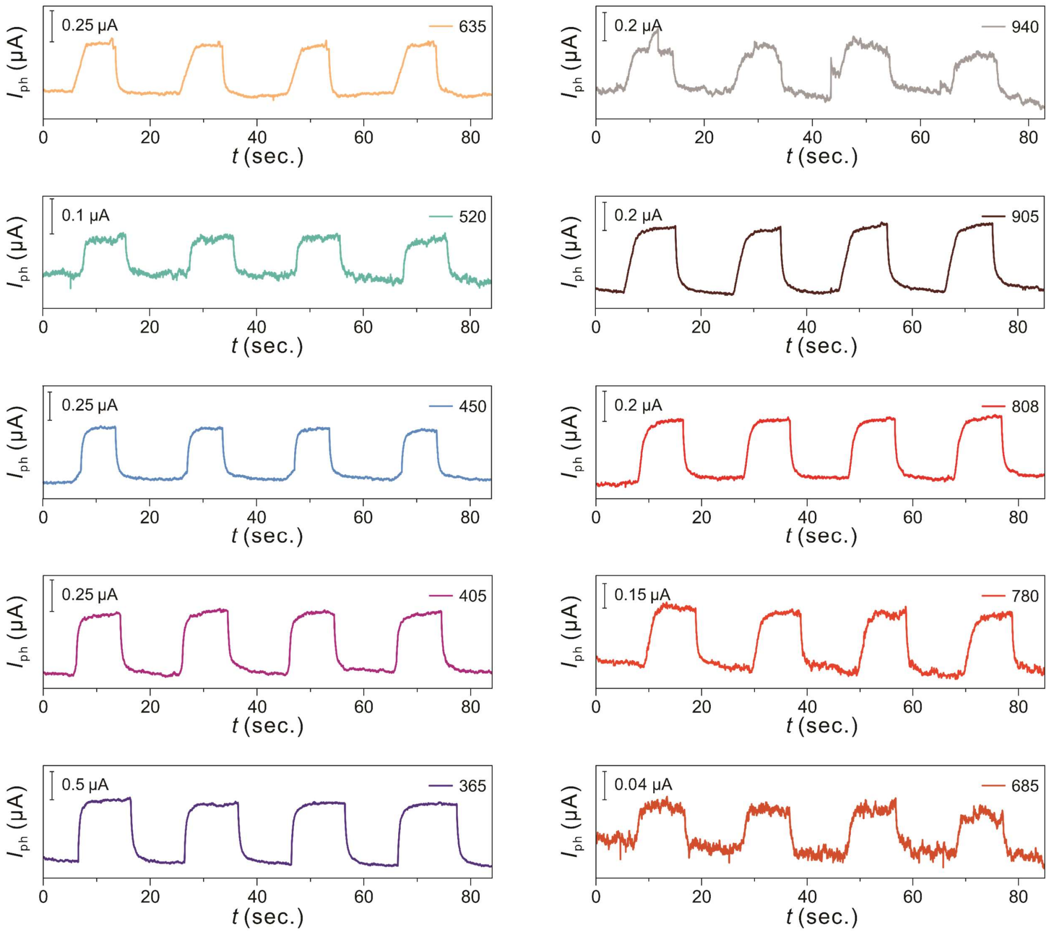

Supplementary Fig. 36 | Photocurrent response curves of *o*-FGaT device under 0.1 V bias condition illuminated by different light sources.

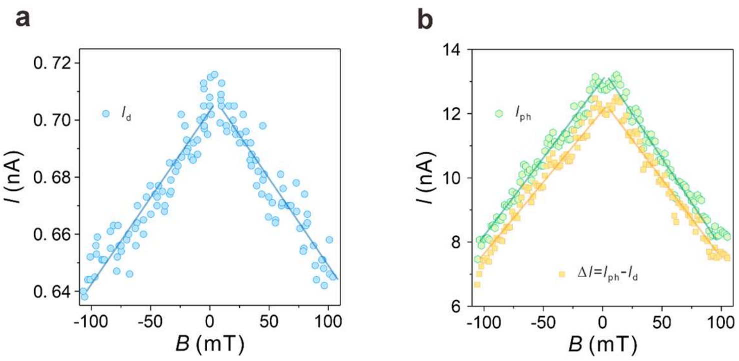

Supplementary Fig. 37 | Comparative analysis of magnetic field-dependent transport in dark and illuminated states. a Magnetic field dependence of the longitudinal current measured in the dark (I_d), exhibiting a characteristic positive magnetoresistance (PMR) effect where the current is symmetrically suppressed by the external magnetic field. **b** The magnetic field dependence of the net photocurrent is obtained by subtracting the dark current background from the total current under illumination ($\Delta I = I_{ph} - I_d$). Measurements were performed using a 520 nm laser with a power density of 5 mW mm^{-2}. The net photocurrent exhibits symmetric modulation, consistent with the symmetry of the dark magnetoresistance effect shown in **a**.

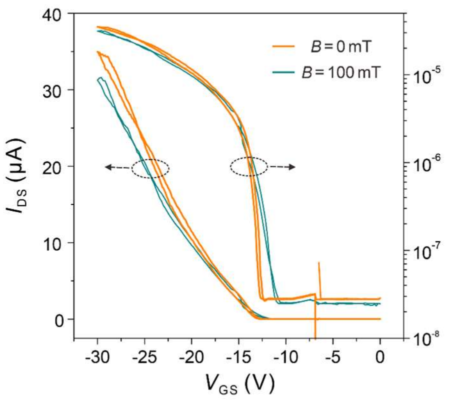

Supplementary Fig. 38 | Transfer characteristics (I_{DS}~V_{GS}) of the *p*-FGaT field-effect transistor (FET) under zero magnetic field (B = 0 mT) and an applied perpendicular magnetic field (B = 100 mT).

The linear-scale I_{DS}~V_{GS} curves (left axis) reveal a distinct decrease in transconductance upon magnetic field application. The transconductance was calculated using Eq. (3):

$$g_m = dI_{DS}/dV_{GS} \tag{3}$$

The semi-logarithmic I_{DS}~V_{GS} curves (right axis) indicate a positive shift in threshold voltage (V_{th}) from −12.5 V to −10.6 V and a degradation in subthreshold slope. The subthreshold slope was calculated using Eq. (4):

$$SS = dV_{GS}/d(\log_{10} I_{DS}) \tag{4}$$

The 2D hole density (n) at a specific gate bias (V_{GS} = −30 V) is calculated based on the MOS capacitor model using Eq. (5):

$$n = C_i|V_{GS} - V_{th}|/e \tag{5}$$

All measurements were performed at a constant drain-source voltage V_{DS} of 10 mV.

Based on the above equations, the fundamental parameters can be calculated as follows.

Conditions	V_{th} (V)	μ_{FE} (cm^2 V^{-1}s^{-1})	n (cm^{-2})	SS (mV dec^{-1})
B = 0 mT	−12.5	290.7	1.26×10^{12}	308.7
B = 100 mT	−10.6	246.0	1.40×10^{12}	415.4

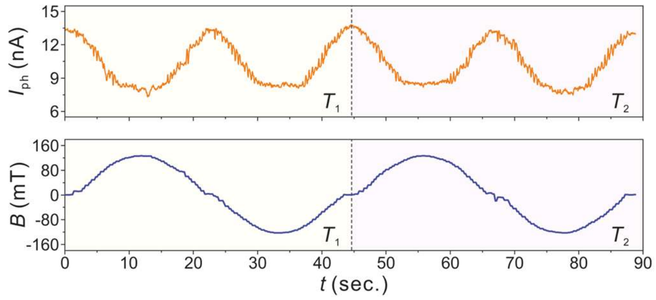

Supplementary Fig. 39 | Magnetic-field-dependent photocurrent modulation under sinusoidal field excitation.

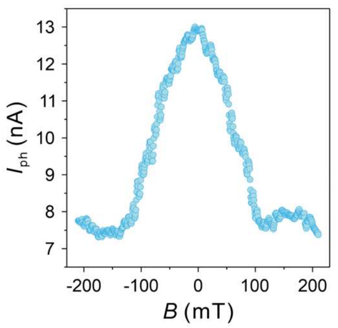

Supplementary Fig. 40 | Photocurrent response under magnetic field. Illumination conditions are λ = 520 nm and P = 5 mW mm^{-2}.

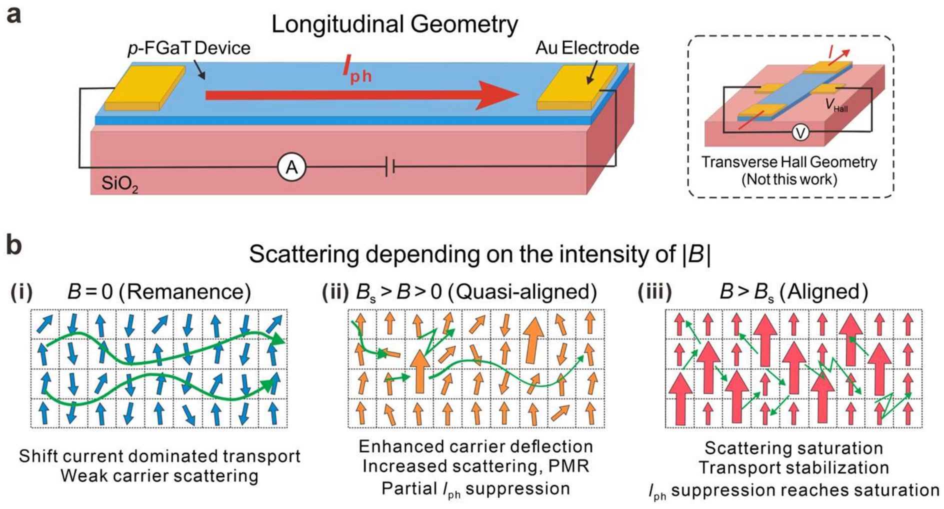

Supplementary Fig. 41 | Schematic illustration of magnetic-field modulation of photocurrent in *p*-FGaT devices. a Device geometry used for longitudinal photocurrent measurements under illumination and applied magnetic field. **b** Conceptual depiction of carrier transport evolution with increasing magnetic field.

Supplementary Note 4: Mechanism of magnetic-field-modulated photocurrent

In our p-FGaT system, the photocurrent generation is dominated by the shift current mechanism. As evidenced by the SHG and STEM characterizations in the main text, oxygen plasma implantation induces local polar distortions and non-centrosymmetric structures. The resulting shift current is governed by this lattice polarity, with its direction fixed by the polar axis and therefore remaining unchanged under reversal of the external magnetic field. This naturally explains why the photocurrent maintains the same polarity under both $+B$ and $-B$ conditions. The experimentally observed symmetric suppression of the photocurrent originates from magnetic-field-dependent modulation of carrier transport, rather than changes in the generation mechanism itself. Magneto-transport measurements (Fig. 4b) reveal a pronounced positive magnetoresistance (PMR) in p-FGaT. With increasing magnetic field, ferromagnetic domains progressively align, leading to enhanced carrier scattering and increased resistance, which in turn reduces the collection efficiency of photogenerated carriers (Supplementary Fig. 39). Since magnetoresistance depends on the magnitude of magnetization rather than its sign, the resulting suppression of the shift current is symmetric with respect to the magnetic field polarity.

$$J_{\text{total}}(B) \approx J_{\text{sh}} \times \left(1 - \delta_{\text{PMR}}(B)\right) \tag{6}$$

- $J_{\text{total}}(B)$: The total photocurrent measured in the experiment, varying with the magnetic field B.
- $\delta_{\text{PMR}}(B)$ (positive magnetoresistance term): The modulation of carrier transport processes by the magnetic field (MR effect).

A key piece of evidence supporting this interpretation is the consistency of the saturation behavior. As shown in Supplementary Fig. 40, the photocurrent suppression saturates at approximately 120 mT, which coincides with the magnetic saturation field B_s determined from MOKE measurements. Such agreement supports a close correlation between the photocurrent modulation and the field-dependent magnetotransport response. In contrast, non-magnetic mechanisms (e.g., Lorentz-force-induced effects) would not reproduce this saturation behavior. In summary, the large shift current generated by inversion-symmetry breaking is symmetrically modulated through magnetoresistance governed by magnetic ordering. This establishes a clear physical picture in which photocurrent generation is dictated by structural polarity, while magnetic order controls carrier transport.

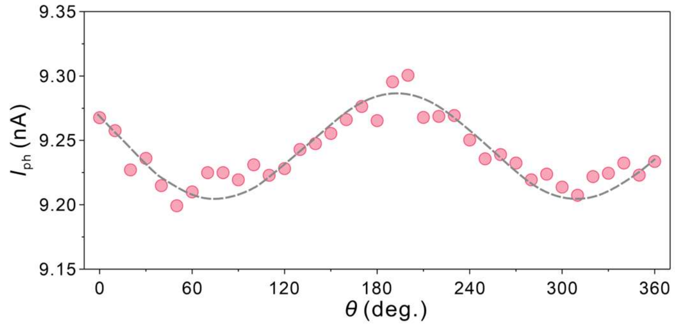

Supplementary Fig. 42 | Circularly polarized photocurrent (I_{ph}-θ) of the *p*-FGaT device (λ = 638 nm, P = 10 mW mm^{-2}).

To further probe the symmetry-related characteristics of the photoresponse, circular photogalvanic effect (CPGE) measurements were performed. As shown in Supplementary Fig. 42, a helicity-dependent photocurrent oscillating with the phase angle of the quarter-wave plate is observed. This behavior reflects asymmetric carrier excitation processes enabled by inversion symmetry breaking in the system.

We emphasize that CPGE can arise in both magnetic and nonmagnetic systems and therefore does not constitute direct evidence for magnetic injection current. Instead, this measurement serves as a complementary characterization, confirming helicity-sensitive optical transitions and the presence of inversion-symmetry-breaking conditions relevant to nonlinear photoresponse.

Importantly, the macroscopic linear photocurrent discussed in the main text remains dominated by shift-current generation together with magnetic-field-modulated transport, and is not governed by CPGE-related mechanisms.

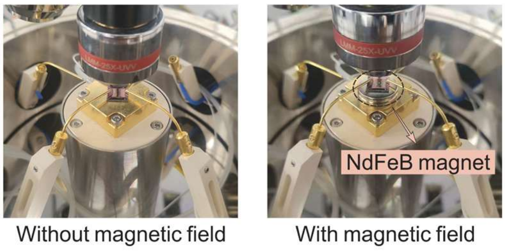

Supplementary Fig. 43 | Digital photograph of photoelectric imaging equipment under an applied magnetic field of 120 mT provided by a commercial NdFeB permanent magnet.

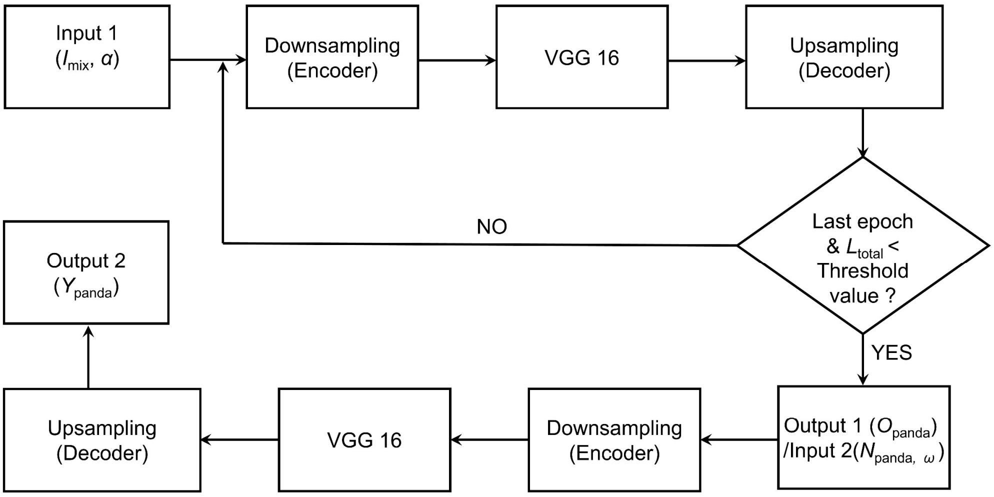

Supplementary Fig. 44 | Flowchart of the physics-driven two-stage training protocol. The magnetic field functions as a physical tunable weight controller to modulate feature transmission.

1. Input 1 (I_{mix}, α): The pipeline initiates by feeding the blended image batch (I_{mix}), and the blending ratio (α). Crucially, α serves as the control variable that dictates the intensity of the magnetic field for the separation task.

2. Downsampling (Encoder): The downsampling stage employs three layers of convolutions to extract features.

3. VGG 16: This module utilizes a pre-trained VGG16 network to process the initial feature maps obtained from the preceding convolutional layers. It aims to extract deeper, high-level semantic representations and refine the latent features, providing a rich multi-scale information basis for the subsequent magnetic modulation.

4. Upsampling (Decoder): This module receives the magnetically modulated feature maps and performs progressive upsampling. Its primary function is to reconstruct the spatial resolution and restore the physically filtered latent features back into the visual image domain to generate the final output.

5. Last epoch & L_{total} < Threshold value? This step determines whether to terminate the training loop. The process proceeds to the next stage only if the current epoch is the final one and the total loss is below the set threshold. Otherwise, the system iterates to further optimize the parameters.

6. Output 1/Input 2 (O_{panda}/O_{cat})/ (N_{panda}, ω): This transitional block bridges the two learning stages. It captures the coarse output from Stage 1 (O_{panda}), which is redefined as the noise-compromised input (N_{panda}) for the subsequent restoration task. Together with the degradation factor (ω), these variables constitute the input tensor for Stage 2.

7. **Output 2 (Y_{panda}):** This final block yields the high-fidelity restored image (Y_{panda}). Following the physics-driven refinement in Stage 2, the output exhibits recovered fine details with noise artifacts effectively removed.

Supplementary Note 5: Computational framework of the magnetically-driven in-sensor computing system

1. Dynamic training optimization mechanism

Initial classification accuracy exhibits stochastic fluctuations when processing composited image inputs through the magnetically controlled hardware simulation system. Progressive accuracy enhancement is achieved through epoch-wise parameter refinement (Epoch ≥ 100) coupled with dynamic magnetic modulation parameter matching, ultimately reaching feature-decoupling equilibrium.

2. Neural network implementation specifications

2.1 Data preparation protocol

A dual-category hybrid image dataset was constructed with 1000 high-resolution samples per class (256 × 256 pixels, RGB channels).

Magnetic-field-dependent image blending (Stage 1 Input) was defined using Eq. (7):

$$I_{\text{mix}} = \alpha I_{\text{cat}} + (1-\alpha) I_{\text{panda}} \tag{7}$$

where:

- $\alpha \in [0, 1]$: Magnetic-field-dependent blending coefficient ($B \propto \alpha$).
- I_{cat}, I_{panda}: Original class image tensors.

Noise Injection (Stage 2 Input):

For the restoration phase, the input is the noise-compromised output from Stage 1. The magnetic field is calibrated to the noise level ω ($B \propto \omega$).

2.2 Feature extraction architecture

Encoder module:

- Input preprocessing: 3-channel RGB images expanded to 256 channels via 3 × 3 convolutions (stride = 1).
- Spatial compression: Max-pooling layers (kernel = 2, stride = 2) reduce resolution from 256^2 to 32^2.
- Feature preservation: Integrated ResNet blocks maintain original feature integrity through skip connections.
- Regularization: Implemented loss (L_1) for pixel-level fidelity was calculated using Eq. (8):

$$L_1 = \frac{1}{N} \sum_{i=1}^{N} \left| F_{\text{encoder}}^{(i)} - F_{\text{initial}}^{(i)} \right| \tag{8}$$

where:

- $F_{\text{encoder}}^{(i)}$: Encoder output feature maps.
- $F_{\text{initial}}^{(i)}$: Baseline features extracted from input images via pre-trained ResNet.

VGG16 transfer learning strategy:

- Initialized with ImageNet-pre-trained weights.
- Multi-scale perceptual loss (L_{perc}) defined as a weighted sum of feature discrepancies across three critical VGG16 layers was calculated using Eq. (9):

$$L_{\text{perc}} = \sum_{k=1}^{3} \lambda_k \, \mathbb{E}\left[\left\| \Phi_k(I_{\text{rec}}) - \Phi_k(I_{\text{gt}}) \right\|_2^2 \right] \tag{9}$$

where:

- Φ_1, Φ_2, Φ_3: Feature maps from *conv1_2*, *conv2_2*, and *conv3_3* layers, respectively.
- $\lambda_1 = 0.2,\ \lambda_2 = 0.5,\ \lambda_3 = 1.0$: Layer-wise importance weights.
- I_{rec}: Reconstructed image tensor.
- I_{gt}: Ground truth image tensor.
- $\mathbb{E}$: math expectation.

2.3 Magnetically-driven decoding mechanism

Functioning as a physical tunable weight controller, the modulation layer explicitly embeds the *p*-FGaT device's attenuation law ($I_{\text{ph}} \propto -B$) to regulate feature channel transmission. By applying physics-derived suppression weights to the encoder outputs, it generates the modulated feature map (X_{mod}) as the input for the decoder, effectively mimicking the device's native magnetic control capabilities.

The magnetically driven weights were generated using Eq. (10):

$$\begin{cases} \gamma(B) = \tanh\left(-\,|W_\gamma| B + b_\gamma\right) \\ \beta(B) = W_\beta + b_\beta \end{cases} \tag{10}$$

Here, the negative constraint ($-|W_\gamma|$) ensures that a negative modulation response with increasing magnetic field, thereby effectively simulating the positive magnetoresistance effect observed in Fig. 4d.

The modulated feature map was calculated using Eq. (11):

$$X_{\text{mod}} = \text{Norm}\left(X_{\text{encoder}}\right) \odot (1+\gamma) + \beta \tag{11}$$

The term $(1 + \gamma)$ functions as a transmission coefficient. As B increases, this coefficient drops below unity, reducing feature channel transmission and thereby effectively suppressing the interference features.

where:

- W_γ, W_β: Trainable weight matrices.
- b_γ, b_β: Bias terms.
- $\odot$: Hadamard product.

Channel-wise normalization was defined using Eq. (12):

$$\text{Norm}\left(X_{\text{encoder}}\right) = \frac{X - \mu_X}{\sigma_X + \epsilon} \tag{12}$$

where:

- μ_X, σ_X: Channel-wise mean and standard deviation.
- $\epsilon = 10^{-5}$: Numerical stability constant.

Physics-driven loss formulation:

To ensure physical consistency during magnetic field modulation, a multi-objective joint constraint loss (L_{phys}) was defined using Eq. (13):

$$L_{\text{phys}} = \lambda_{\text{reg}} L_{\text{reg}} + \lambda_{\text{bound}} L_{\text{bound}} \tag{13}$$

2.3.1 Regularization constraint (suppressing parameter overfitting) was calculated using Eq. (14):

$$L_{\text{reg}} = \|W_\gamma\|_F^2 + \|W_\beta\|_F^2 \tag{14}$$

where $\|\cdot\|_F^2$ denotes the Frobenius norm.

2.3.2 Parameter boundary constraint (ensuring modulation factors adhere to physical limits) was calculated using Eq. (15):

$$L_{\text{bound}} = \mathbb{E}\left[\max\left(|Y| - 0.9, 0\right)^2\right] \tag{15}$$

Parameters:

- $\lambda_{\text{reg}} = 1.0$: Regularization weight.
- $\lambda_{\text{bound}} = 0.5$: Boundary penalty coefficient.

2.3.3 Decoder architecture:

Following the modulation phase, this block serves as a reconstruction engine. It performs progressive upsampling (PixelShuffle, scale factor = 8) to reconstruct the spatial resolution and restore the physically filtered latent features back into the visual image domain.

2.4 Image enhancement (restoration) framework (Stage 2)

Leveraging a cooperative architecture reuse mechanism, the system directly employs the unified encoder-decoder structure for both the initial feature separation and the subsequent image restoration, effectively mitigating the complexity of multi-task networks. In the restoration phase, the magnetic field is systematically diminished to progressively relax the suppression weights. This process explicitly mimics the recovery of photocurrent observed in the *p*-FGaT device. As the external magnetic field is withdrawn, the previously suppressed signal amplitude gradually returns to its original state. By synchronizing this computational weight relaxation with the decoding process, the network allows fine image details to re-emerge, effectively eliminating reconstruction artifacts and recovering high-fidelity visuals. This magnetically-driven in-sensor computing architecture enables adaptive visual processing, preserving robust separation capabilities while achieving a significant 10.4 dB PSNR improvement (baseline: 28.3 dB → restored: 38.7 dB) for degraded images.

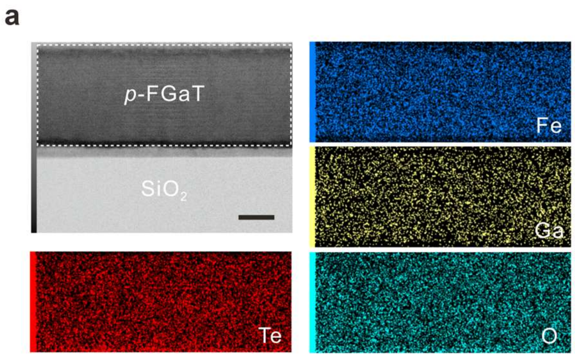

b

Elements	Atomic %
Fe	40.44
Ga	13.70
Te	30.10
O	15.76

Supplementary Fig. 45 | Quantitative elemental analysis of oxygen-implanted *p*-FGaT validating the theoretical model. a TEM image and corresponding large-area EDS elemental mapping of Fe, Ga, Te, and O. **b** Quantitative atomic percentages of each element. Scale bar, 25 nm in **a**.

Supplementary References

1. Gong Y, et al. Reconfigurable and nonvolatile ferroelectric bulk photovoltaics based on 3R-WS_2 for machine vision. *Nat. Commun.* **16**, 230 (2025).

2. Wang Z, et al. Giant infrared bulk photovoltaic effect in tellurene for broad-spectrum neuromodulation. *Light Sci. Appl.* **13**, 277 (2024).

3. Zeng Z, et al. Dual polarization-enabled ultrafast bulk photovoltaic response in van der Waals heterostructures. *Nat. Commun.* **15**, 5355 (2024).

4. Jiang J, et al. Flexo-photovoltaic effect in MoS_2. *Nat. Nanotechnol.* **16**, 894-901 (2021).

5. Ellmer K. Preparation routes based on magnetron sputtering for tungsten disulfide (WS_2) films for thin-film solar cells. *Phys. Status Solidi B* **245**, 1745-1760 (2008).

6. Yang SY, et al. Above-bandgap voltages from ferroelectric photovoltaic devices. *Nat. Nanotechnol.* **5**, 143-147 (2010).

7. Osterhoudt GB, et al. Colossal mid-infrared bulk photovoltaic effect in a type-I Weyl semimetal. *Nat. Mater.* **18**, 471-475 (2019).

8. Aftab S, Shehzad MA, Salman Ajmal HM, Kabir F, Iqbal MZ, Al-Kahtani AA. Bulk photovoltaic effect in two-dimensional distorted $MoTe_2$. *ACS Nano* **17**, 17884-17896 (2023).

9. Ma J, et al. Nonlinear photoresponse of type-II Weyl semimetals. *Nat. Mater.* **18**, 476-481 (2019).

10. Li Y, et al. Enhanced bulk photovoltaic effect in two-dimensional ferroelectric $CuInP_2S_6$. *Nat. Commun.* **12**, 5896 (2021).

11. Dong Y, et al. Giant bulk piezophotovoltaic effect in 3*R*-MoS_2. *Nat. Nanotechnol.* **18**, 36-41 (2023).

12. Liu Y, Dong Q, Fang Y, Lin Y, Deng Y, Huang J. Fast growth of thin $MAPbI_3$ crystal wafers on aqueous solution surface for efficient lateral-structure perovskite solar cells. *Adv. Funct. Mater.* **29**, 1807707 (2019).

13. Quattropani A, et al. Tuning photovoltaic response in Bi_2FeCrO_6 films by ferroelectric poling. *Nanoscale* **10**, 13761-13766 (2018).

14. Grinberg I, et al. Perovskite oxides for visible-light-absorbing ferroelectric and photovoltaic materials. *Nature* **503**, 509-512 (2013).

15. Nakamura M, et al. Shift current photovoltaic effect in a ferroelectric charge-transfer complex. *Nat. Commun.* **8**, 281 (2017).

16. Brody PS. High voltage photovoltaic effect in barium titanate and lead titanate-lead zirconate ceramics. *J. Solid State Chem.* **12**, 193-200 (1975).

17. Ichiki M, Maeda R, Morikawa Y, Mabune Y, Nakada T, Nonaka K. Photovoltaic effect of lead lanthanum zirconate titanate in a layered film structure design. *Appl. Phys. Lett.* **84**, 395-397 (2004).

18. Pal S, et al. Giant photovoltaic response in band engineered ferroelectric perovskite. *Sci. Rep* **8**, 8005 (2018).

19. Sun Z, et al. A Photoferroelectric Perovskite-Type Organometallic Halide with Exceptional Anisotropy of Bulk Photovoltaic Effects. *Angew. Chem. Int. Ed* **55**, 6545-6550 (2016).

20. Zhang YJ, et al. Enhanced intrinsic photovoltaic effect in tungsten disulfide nanotubes. *Nature* **570**, 349-353 (2019).

21. Nechache R, et al. Photovoltaic properties of Bi_2FeCrO_6 epitaxial thin films. *Appl. Phys. Lett.* **98**, (2011).

22. Zhang G, et al. New high T_c multiferroics $KBiFe_2O_5$ with narrow band gap and promising photovoltaic effect. *Sci. Rep.* **3**, 1265 (2013).

23. Choi T, Lee S, Choi YJ, Kiryukhin V, Cheong SW. Switchable ferroelectric diode and photovoltaic effect in $BiFeO_3$. *Science* **324**, 63-66 (2009).

24. Matsuo H, Noguchi Y, Miyayama M. Gap-state engineering of visible-light-active ferroelectrics for photovoltaic applications. *Nat. Commun.* **8**, 207 (2017).

25. Asada S, Shinokita K, Watanabe K, Taniguchi T, Matsuda K. Nonlinear photovoltaic effects in monolayer semiconductor and layered magnetic material hetero-interface with *P*- and *T*-symmetry broken system. *Nat. Commun.* **16**, 4827 (2025).

26. Zenkevich A, Matveyev Y, Maksimova K, Gaynutdinov R, Tolstikhina A, Fridkin V. Giant bulk photovoltaic effect in thin ferroelectric $BaTiO_3$ films. *Phys. Rev. B* **90**, 161409 (2014).

27. Fridkin VM, Grekov AA, Rodin AI. The bulk photovoltaic effect in the crystals without a center of symmetry. *Ferroelectrics* **43**, 99-108 (1982).

28. Laurenti M, et al. Nanobranched ZnO structure: *p*-type doping induces piezoelectric voltage generation and ferroelectric–photovoltaic effect. *Adv. Mater.* **27**, 4218-4223 (2015).

29. Sturman BI, Firdkin VM. Photovoltaic and photo-refractive effects in noncentrosymmetric materials. *Ferroelectrics*, (1992).

30. Mistewicz K, Nowak M, Stróż D. A Ferroelectric-photovoltaic effect in SbSI nanowires. *Nanomaterials* **9**, 580 (2019).

31. Nadupalli S, Kreisel J, Granzow T. Increasing bulk photovoltaic current by strain tuning. *Sci. Adv.* **5**, eaau9199 (2019).

32. Burger AM, et al. Direct observation of shift and ballistic photovoltaic currents. *Sci. Adv.* **5**, eaau5588 (2019).
