# Supplementary material for: Giant bulk photovoltaic effect in an iron-based magnetic semiconductor
Source: Nat Commun. 2026 Jun 16;17:7629. doi: 10.1038/s41467-026-74473-3 (PMC13429580; doi:10.1038/s41467-026-74473-3)
Supplement: Supplementary file 2 — Transparent Peer Review file [file 41467_2026_74473_MOESM2_ESM.pdf]

nature portfolio

Peer Review File

Giant Bulk Photovoltaic Effect in an Iron-Based Magnetic Semiconductor

Corresponding Author: Dr Liang Hu

This file contains all reviewer reports in order by version, followed by all author rebuttals in order by version.

Version 0:

Reviewer comments:

Reviewer #1

(Remarks to the Author)
In their manuscript entitled “Room-Temperature Magnetically Tunable Iron-Based Photovoltaics” by M. Cheng and co-workers, the authors have demonstrated oxygen-implanted Fe3GaTe2 shows the superior performance of bulk photovoltaic properties with large short circuit-current exceeding 30 A cm2, and robust ferrogamgentic phase above room temperature (350 K). The change of electronic, and magnetic properties are well supported by various experimental and theoretically calculated results. Moreover, the photocurrent can be manipulated by the modest magnetic field of 120 mT, which also enables to magnetic programable nuro-vision system. However, there are some points that need to be clarified and the questions and comments are described below.

1) The DFT calculation results clearly show that oxygen implantation causes a bandgap opening and ferromagnetic interactions in the oxygen-implanted Fe_3GaTe_2. In contrast, the bandgap value derived from the DFT calculation (less than 1 eV) differs significantly from the value determined by photoluminescence spectroscopy (2.4 eV). What is the physical reason for this large discrepancy?

2) The authors present photoelectric imaging before and after the application of a magnetic field of 120 mT in Fig. 4e, demonstrating the magnetic field (MF) enhancement and attenuation processes. Taking into account the advantages of this in oxygen-implanted Fe_3GaTe_2, the authors claim to demonstrate the implementation of convolutional processing and in-sensor computing for blended image classification and restoration in Fig. 4. However, the explanation of the process in the main text and supporting information is unclear. Thus, it is difficult for readers to understand how the magnetic properties of the oxygen-implanted Fe_3GaTe_2 are used in convolutional processing and in-sensor computing.

I think this study will contribute to the researches in the emerging research field of magnetic photovoltaics. However, the authors should clarify some points described above. To meet the higher standard and criteria of Nature Communications, the authors should provide the clear explanation and make revision for publication in Nature Communications.

Reviewer #2

(Remarks to the Author)
The manuscript entitled “Room-Temperature Magnetically Tunable Iron-Based Photovoltaics” (NCOMMS-25-64518-T) presents a highly valuable study that marks a breakthrough in magneto-optoelectronics. By using oxygen-plasma treatment to induce a metal-semiconductor transition in two-dimensional iron-based magnetic metal Fe3GaTe2, the authors successfully demonstrate room-temperature magnetically tunable BPV effects. This work signifies a pivotal development in the field compared to some work published recently (e.g., Nat. Commun. 16, 4827 (2025)), providing compelling evidence of room-temperature magnetically controllable photocurrents under zero bias with potential applications in sensing and neuromorphic devices. The manuscript is well-structured and articulately written; I recommend its publication in Nature Communications following moderate revisions to enhance clarity and reproducibility without compromising its significance.

1.It is recommended to provide in-situ MOKE characterization of a Fe3GaTe2 flake for various oxygen-plasma treatment durations, in order to better illustrate the evolution of magnetic properties.
2.To clarify the magnetoelectric-like coupling mechanism central to device operation, piezoresponse force microscopy

(PFM) should be utilized to confirm whether the sample also exhibits ferroelectricity; if not, the nature of the coupling and how it differs from conventional magnetoelectric coupling should be discussed.
3.To ensure the repeated photoelectric imaging, please provide detailed parameters and conditions used in the single-point imaging equipment with a panda-shaped mask.
4.Statistical validation of the BPV effects using multiple devices would help verify the reproducibility and robustness of the findings.
5.Although nine-month stability is mentioned, supplementary data depicting the temporal evolution of key performance metrics (e.g., photocurrent) under ambient conditions should be included to substantiate the claim.
6.The band structure of intrinsic Fe_3GaTe_2 from theoretical modeling should be provided to better reveal the transition from metal to semiconductor.
7.It is noticed that there are a few silicon-based magneto-optoelectronic devices (e.g., Adv. Mater. 226, 8059 (2014)), also exhibiting magnetically-sensitive photocurrent modulation under zero bias. In comparison, what are the advantages of this work in terms of concept and performance?
8.A considerable BPV coefficient (0.25 V^{-1}) is claimed in this work but related literature is not properly reviewed. Authors should provide some discussions to help readership understand such excellent performance emerging in this material.

Reviewer #3

(Remarks to the Author)

This manuscript reports the synthesis and characterization of oxygen-implanted Fe_3GaTe_2 (p-FGaT), a single-phase material that exhibits a Bulk Photovoltaic Effect (BPVE) coupled with room-temperature ferromagnetism. It demonstrated a BPV that operates above room temperature and can be tuned by a modest magnetic field of 120 mT. This development directly addresses the major practical limitations—low temperature and Tesla-level magnetic fields—that have constrained previous magnetic photovoltaic systems. However, I do not find they provided enough data and sufficient analysis to support their strong conclusions. I have major concerns, particularly regarding the magnetic tunability of the BPVE.

1. Clarification on the magnetic order dependence of photocurrent:
The primary objective of this paper is to demonstrate the magnetic tunability of the bulk photovoltaic effect (BPVE) related to the magnetic order of the system. However, no direct relation between the magnetic order and the BPVE has been established in this work. One would naively expect a significant difference in the photocurrent for ± magnetic fields corresponding to the magnetic order. In the experiment, there is an overall suppression of the BPGE with magnetic field, but is it related to the magnetic order? If so, why is the response the same for both positive and negative fields? Ideally, it would also be informative to investigate the magnetic controllability of the photocurrent above the magnetic transition temperature.
2. Clarification on the separation of origins of the photocurrent:
This is partly related to the first question. In magnetic materials, a linear injection current arises; however, it remains unclear whether this component is actually observed in the experiments. Unlike the linear shift current, the linear injection current is odd under time reversal. Assuming the shift current is time-reversal even, one would expect the measured response to show a (shift ± injection) response depending on the magnetic field direction. However, their measurements show the same current for both positive and negative magnetic fields. This raises the question of how they confirm the presence of a linear injection current.
3. Clarification on theoretical modeling of the extra oxygen:
The authors model the extra oxygen atom by placing two oxygen atoms at specific sites. This effectively creates a new material, which can significantly impact the band structure. Is this approach consistent with the actual concentration of the additional oxygen?
4. Absence of the photocurrent in i-FGaT:
The manuscript reports that the i-FGaT sample exhibits a significant second-harmonic generation (SHG) signal, indicating broken inversion symmetry. However, it remains unclear why this same sample does not exhibit a measurable photocurrent response. Given that SHG and the bulk photovoltaic effect (BPVE) are both sensitive to inversion symmetry breaking, a discussion is warranted to clarify the apparent discrepancy.
While the manuscript reports a very large photocurrent in this material at room temperature, I cannot recommend it for publication in its current form. To justify its potential, the authors must establish a clear connection between the observed photocurrent and the underlying magnetic order, demonstrating the material’s magnetic tunability. Without such a link, the significance of the large photocurrent remains unclear.

Minor Comments
1. Circular photogalvanic effect:
Another intriguing aspect of the BPGE is its response to circularly polarized light. Given that the C3 symmetry is broken in this system, one would expect the circular photogalvanic effect to appear. It is unclear why the study does not include an investigation of this effect.
2. Clarity on "PT-symmetry":
In line 329, the authors mention seeing some effect of PT symmetry. Can this symmetry be attained in this material by changing the spin polarization? If not, then the meaning of the first few lines of the paragraph is unclear.
3. Clarity on "bipolar nature":
The authors write in line 184: “Furthermore, the system exhibits bipolar semiconducting characteristics, with majority spins dominating the VBM and minority spins dominating the CBM.” This is not clear from the band structure and DOS plot.
4. Photocurrent vs. wavelength:
It would be helpful if the authors presented the photocurrent as a function of light wavelength, similar to Figure 3(b). Does the

photocurrent show sub-band responses?

Version 1:

Reviewer comments:

Reviewer #1

(Remarks to the Author)
In their manuscript entitled “Room-Temperature Magnetically Tunable Iron-Based Photovoltaics” by M. Cheng and co-workers, the authors have appropriately revised the manuscript according to my questions and comments. I think that the authors have made major revisions to meet the higher standards and criteria for publication in Nature Communications. Thus, I recommend publication in Nature Communications.

Reviewer #2

(Remarks to the Author)
The authors have addressed my questions with full details and the manuscript can be accepted as it is now.

Reviewer #3

(Remarks to the Author)
The authors have addressed several of my earlier questions regarding modeling of oxygen incorporation and the relation of photocurrent and SHG in i-FGT. The new data on CPGE and sub-gap response are valuable addition to the manuscript. These revisions improve the completeness of experimental characterization.
Despite these improvements, my two primary concerns regarding the claim of magnetic injection current remain unresolved.

The authors seem to attribute a portion of the ~20% reduction in photocurrent (compared to ~8% reduction in the dark condition) in the presence of the magnetic field to a linear magnetic injection current, stating in the main text that the PMR alone cannot explain it. This is qualitatively contradicting the definition of magnetic injection current. The magnetic injection current will change sign upon reversing the magnetic order. However, experiments show the same sign of current change for + and - fields. There is no way to rationalize this is a magnetic injection effect.

The authors present new CPGE data and argue that its observation supports time-reversal-symmetry–broken injection current. This is not true. CPGE occurs as an injection current in nonmagnetic systems. But they studied a magnetic material here. There is no direct connection between CPGE and claimed magnetic injection current in this work.

In conclusion, I stress two things. (a) Since the suppression of photocurrent is argued to be related to the MR, the authors should show a plot of the MR with the magnetic field to support this interpretation. (b) The authors should explicitly state that if magnetic injection current were present, there should be opposite signs between photocurrent measured at positive and negative magnetic fields.

With these clarifications, I would be in a better position to comment on the suitability of the manuscript for publication.

Version 2:

Reviewer comments:

Reviewer #2

(Remarks to the Author)
The authors report the room-Temperature bulk photovoltaic effects in p-FGaT iron-based devices. The magnetic tunability may be not precise as Reviewer 3 mentioned and the authors have changed that to ordinary magnetic resistancein revision. I think this work may not report magnetic tunability of bulk photovoltaics, but it is still very interesting in reporting an excellent bulk photovoltaic effect which hasone of the best bulk photovoltaic coefficient as high as 0.25 V^{-1}, several orders of magnitude higher than conventional bulk ferroelectrics (typically 10-4-10-5V^{-1}) and comparable to state-of-the-art low-dimensional systems such as WS2 nanotubes. This giant bulk photovoltaic performance in van der Waals ferromagnetic semiconductor is very important for potential photo-magnetoelectric applications. I think although the absence of magnetic tenability in bulk p-FGaT is a pity but this work isstill very important with significant new insight in bulk photovoltaics in ferromagnetic semiconductors. I think after changingto new focus in bulk photovoltaics in van der Waals ferromagnetic semiconductors,the work deservesto publish in Nature Communications.

Reviewer #3

(Remarks to the Author)
The authors revised their interpretation on the magnetic BPVE and changed it to ordinary magnetoresistance. This might be solid in theory. But, in this sense, this phenomenon reported in this work looks very trivial.

Version 3:

Reviewer comments:

Reviewer #2

(Remarks to the Author)
The authors have addressed my concerns and the work can be accepted now.

(Remarks on code availability)

Reviewer #3

(Remarks to the Author)
The revision is ok with me.

(Remarks on code availability)

Open Access This Peer Review File is licensed under a Creative Commons Attribution 4.0 International License, which permits use, sharing, adaptation, distribution and reproduction in any medium or format, as long as you give appropriate credit to the original author(s) and the source, provide a link to the Creative Commons license, and indicate if changes were made.
In cases where reviewers are anonymous, credit should be given to 'Anonymous Referee' and the source.
The images or other third party material in this Peer Review File are included in the article’s Creative Commons license, unless indicated otherwise in a credit line to the material. If material is not included in the article’s Creative Commons license and your intended use is not permitted by statutory regulation or exceeds the permitted use, you will need to obtain permission directly from the copyright holder.
To view a copy of this license, visit https://creativecommons.org/licenses/by/4.0/

Response to Reviewers' Comments

Reviewer #1

General Comment: *In their manuscript entitled "Room-Temperature Magnetically Tunable Iron-Based Photovoltaics" by M. Cheng and co-workers, the authors have demonstrated oxygen-implanted Fe_3GaTe_2 shows the superior performance of bulk photovoltaic properties with large short circuit-current exceeding 30 A cm^2, and robust ferromagnetic phase above room temperature (350 K). The change of electronic, and magnetic properties are well supported by various experimental and theoretically calculated results. Moreover, the photocurrent can be manipulated by the modest magnetic field of 120 mT, which also enables to magnetic programable nuro-vision system. However, there are some points that need to be clarified and the questions and comments are described below.*

Reply: We sincerely thank the reviewer for the highly encouraging assessment and for recognizing the significance of our work, particularly the realization of robust room-temperature ferromagnetism and superior BPV performance in oxygen-implanted Fe_3GaTe_2. We also appreciate the constructive comments pointing out the discrepancy in the bandgap analysis and the need for greater clarity in the neuromorphic application section. To address these concerns, we have incorporated high-precision hybrid functional calculations in the revised manuscript, which now provide a theoretical bandgap (~1.02 eV) that aligns perfectly with the experimental photoresponse cutoff. Additionally, we have comprehensively restructured the discussion on the neuromorphic vision system to explicitly elucidate the physical mechanism governing the magnetic programming of the neural network weights. We believe these substantial revisions have significantly improved the physical rigor and clarity of the manuscript.

Comment-1: *The DFT calculation results clearly show that oxygen implantation causes a bandgap opening and ferromagnetic interactions in the oxygen-implanted Fe_3GaTe_2. In contrast, the bandgap value derived from the DFT calculation (less than 1 eV) differs significantly from the value determined by photoluminescence spectroscopy (2.4 eV). What is the physical reason for this large discrepancy?*

Response: Thank you for raising this important point. We agree that conventional PBE+U calculations tend to underestimate band gaps due to the intrinsic self-interaction error[1, 2] and the absence of nonlocal exchange. To address this discrepancy, we performed additional hybrid-functional calculations using the HSE functional[3, 4, 5, 6] with a Hartree-Fock exchange mixing ratio of 32%.

With HSE, the band gap increases to 1.02 eV in the spin-up channel, which is in good agreement with the experimental photoluminescence result. Meanwhile, the spin-down channel exhibits a larger gap of 1.55 eV, reflecting the spin-dependent electronic structure induced by oxygen implantation. These results confirm that the small band gap predicted by PBE+U originates from methodological limitations rather than an intrinsic inconsistency with experiment.
Crucially, we suggest that this calculated gap should be correlated with the onset of the photoresponse rather than the PL emission peak. As shown in Fig. 3b of the manuscript, the *p*-FGaT device exhibits a broad spectral response extending to the near-infrared region with a cutoff wavelength around 1160 nm (corresponding to ~1.07 eV). The HSE-calculated gap (1.02 eV) is in excellent agreement with this experimental cutoff energy. While PL (2.39 eV) originates from the primary optical transitions between localized states or specific sub-bands, the calculated fundamental gap corresponds to the absorption edge that enables the broadband BPV effect observed in Fig. 3b.

In the main text, we revised it as follows:

"**Theoretical calculation**

Spin-polarized density functional theory (DFT) calculations were conducted using the Vienna Ab initio Simulation Package (VASP)[76-78]. These calculations adhered to the Perdew-Burke-Ernzerhof functional level of theory[79]. To accurately model various elements, we employed projector-augmented wave potentials, specifically targeting the orbitals of Fe ($3p^6 3d^7 4s^1$), Te ($5s^2 5p^4$), Ga ($4s^2 4p^1$), O ($2s^2 2p^4$), and H ($1s$)[80, 81]. To account for the localized nature of Fe 3*d* electrons, the Hubbard *U* correction was applied with an effective *U* (U_{eff}) value of 4.0 Fe[82]. A plane-wave energy cutoff of 700 eV was adopted. The Brillouin zone was sampled using a Γ-centered 15 × 15 × 3 k-point mesh. Structural optimizations were carried out until the forces on all atoms were less than 0.001 eV/Å. To rigorously correct the bandgap underestimation typical of PBE+U functionals, we employed the Heyd-Scuseria-Ernzerhof (HSE) hybrid functional with a Hartree-Fock mixing parameter of 0.32. The energy cutoff was set to 600 eV, and a 7 × 7 × 2 k-point mesh was used for these computationally demanding calculations[83-86].

As a magnetic semiconductor, *p*-FGaT also demonstrates wavelength(*λ*)-dependent photoelectric conversion from 360 to 1100 nm without bias (Fig. 3b; Supplementary Fig. 19). The peak responsivity (*R*) of 0.36 A/W occurs at 600 nm, decreasing towards the near-infrared with a cutoff at 1160 nm (~1.07 eV). Notably, the photoresponse extends to photon energies below ~~the E_g~~ the optical bandgap (E_g ≈ 2.39 eV), first-principles calculations using the HSE functional reveal a fundamental electronic gap of ~1.02 eV (spin-up channel), which aligns well with this experimental cutoff. This confirms that the broadband BPV

response extending to the near-infrared region originates from the intrinsic electronic structure captured by the hybrid functional theory."

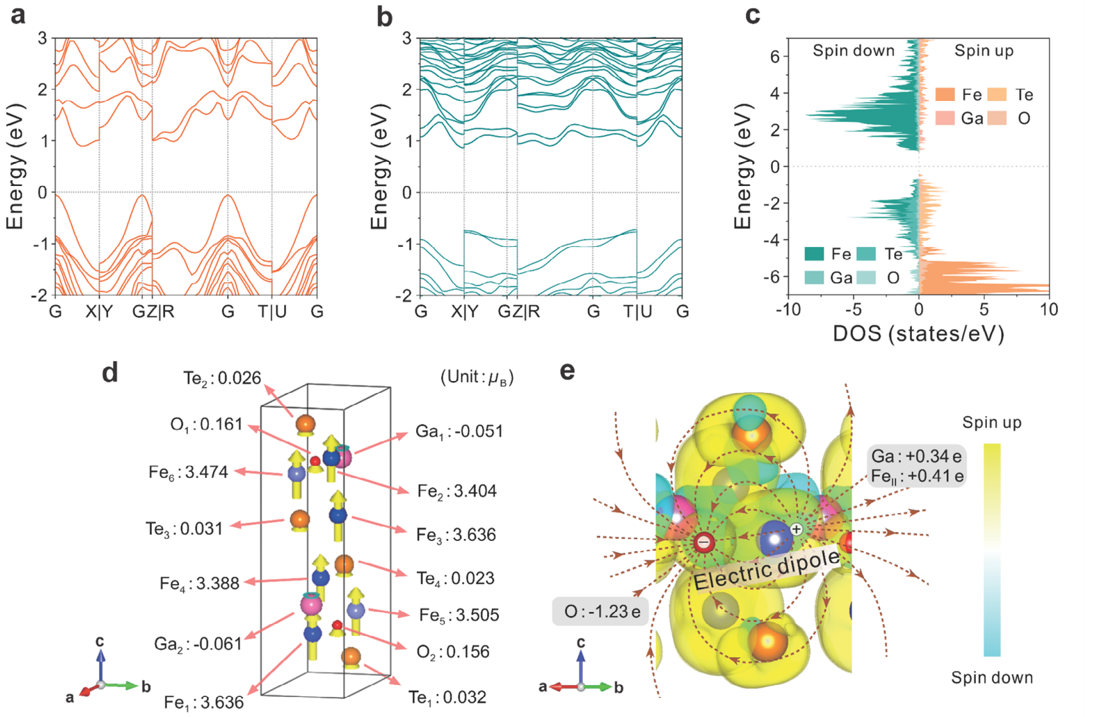

Fig. 2 | First-principles calculations and polar analysis of intralayer-oxygen-implanted *p*-FGaT. Spin-majority (Up) (**a**) and spin-minority (Down) (**b**) energy band structures, both demonstrating the nature of indirect semiconductor. **c** Spin-polarized density of states (DOS) diagram. The weights of different elements are identified with gradient colors. **d** Calculated Bohr magnetic moments and spin vector arrows of different atoms in the supercell. **e** Illustrative electric dipole superimposed in the spin density distribution. The Bader charges of Fe_{II}, Ga, and O atoms are shown nearby.

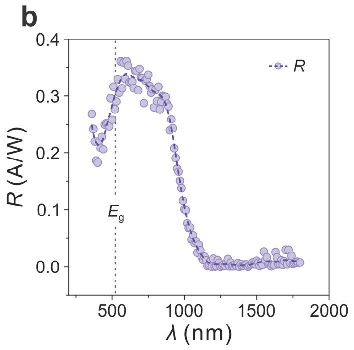

Fig. 3 | Semiconducting and BPV properties of FGaT device. b Wavelength-dependent responsivity (*R*-*λ*) curve of *p*-FGaT device.

Comment-2: *The authors present photoelectric imaging before and after the application of a magnetic field of 120 mT in Fig. 4e, demonstrating the magnetic*

field (MF) enhancement and attenuation processes. Taking into account the advantages of this in oxygen-implanted Fe_3GaTe_2, the authors claim to demonstrate the implementation of convolutional processing and in-sensor computing for blended image classification and restoration in Fig. 4. However, the explanation of the process in the main text and supporting information is unclear. Thus, it is difficult for readers to understand how the magnetic properties of the oxygen-implanted Fe_3GaTe_2 are used in convolutional processing and in-sensor computing.

Response: We sincerely thank the reviewer for this insightful comment. We agree that the previous manuscript did not sufficiently bridge the gap between the material's physical properties (specifically the magnetic-field-induced photocurrent attenuation shown in Fig. 4e) and its algorithmic implementation in the neural network.

To address this, we have comprehensively revised the main text (Fig. 5), Supplementary Note 4, and Supplementary Fig. 38 to explicitly articulate how the device's magnetic properties are mapped into the convolutional processing.

In the main text, we revised it as follows:

"**Fig. 5 | ~~Implementation of convolutional processing and in-sensor computing for blended image classification and restoration~~ Implementation of magnetically-programmable in-sensor computing for feature separation and restoration. a** Schematic diagram of the ~~convolutional processing blended image. The network performs image classification and restoration through a two-step process of encoder and decoder. Image restoration utilizes the same model structure. **b** Image separation and restoration processes corresponding to different magnetic fields and epoch.~~ physics-driven convolutional processing workflow. The network leverages the *p*-FGaT device as a physical tunable weight controller to execute a two-stage process: interference suppression (Stage 1) and detail recovery (Stage 2) within a unified framework. **b** Visualization of the magnetically-driven progressive feature disentanglement and restoration. The panels illustrate the evolution of image features under varying magnetic field intensities across training epochs (0→100 epochs).

Leveraging the magnetic field (MF) linear modulation of photocurrents and device stability, we devised a magnetically-programmable neural network (MNN) hardware simulation architecture ~~with MF-controlled classification capabilities. Fig. 5a outlines the core workflow, where gradient magnetic constraints facilitate feature-decoupled classification and image inpainting of hybrid images.~~ Fig. 5a outlines the core workflow, where the *p*-FGaT device functions as a

physical tunable weight controller, utilizing gradient magnetic constraints to facilitate feature-decoupled separation and image inpainting of hybrid images. In this demonstration, cat-panda hybrid images undergo feature downsampling, followed by a decoding phase using MF enhancement to suppress cat features while preserving panda characteristics, achieving precise feature separation. Additionally, degraded image reconstruction via inverse MF modulation resulted in high-precision restored images (for details see Supplementary Fig. 38).

To assess system performance, we conducted a proof-of-concept experiment. ~~During encoding, cat-panda hybrid images underwent feature downsampling, followed by a decoding phase using MF enhancement to suppress cat features while attenuating panda characteristics, achieving precise feature separation. Additionally, degraded image reconstruction via inverse MF modulation resulted in high-precision restored images (256×256 pixels). Fig. 5b illustrates the process of feature separation and detail repair for hybrid images. Stage 1 (left panels) shows progressive feature disentanglement under increasing MF intensities (0→100 epochs), with MF enhancement driving hierarchical pattern formation: initial blended inputs (left) progress from rough separation (epoch 25) to complete separation (epoch 100), quantified by MF values during convolutional computation (see Supplementary Note 3). Stage 2 (right panels) demonstrates magnetic-attenuation-driven restoration over 100 epochs, where diminishing MF intensities progressively refine image details. Initial restoration of details and colors occurs at the 25th epoch, with final realistic restoration (epoch 100) achieved by magnetically controlled multiscale fusion.~~ In Stage 1, the MF (B) was linearly coupled to the image blending ratio α (where $B \propto \alpha$) to serve as the governing control variable. Crucially, we translated the device's signal enhancement into a computational suppression mechanism. Specifically, increasing MF induces negative modulation that reduces feature channel transmission, mimicking photocurrent attenuation. This process effectively fades out interference features (e.g., the cat) in proportion to the applied field (for details see Supplementary Note 4). This progressive disentanglement is visualized in Fig. 5b (left pannels), where increasing MF intensities (0→100 epochs) drive hierarchical pattern formation as initial blended inputs progress from rough separation (epoch 25) to complete separation (epoch 100), quantified by MF values during convolutional computation.

This physics-driven logic extends to the restoration phase (Stage 2), where the process is reversed to recover image fidelity. Initially, the output images are compromised by noise artifacts. To address this, the MF is calibrated to the noise level ω (where $B \propto \omega$), utilizing the device's signal attenuation to effectively recover high-quality pixels. Subsequently, by progressively diminishing the MF, the suppression features are relaxed, which mimics the recovery of photocurrent as the magnetic field is removed (for details see Supplementary Note 4). This gradual magnetic field reduction allows the

previously suppressed fine details to re-emerge while keeping noise minimal, enabling precise image reconstruction. Fig. 5b (right panels) tracks this magnetic-attenuation-driven restoration, where diminishing MF over 100 epochs progressively refine the image. Initial restoration of details and colors appears at the 25th epoch, ultimately achieving final realistic restoration (epoch 100) achieved by magnetically controlled multiscale fusion.Collectively, these demonstrations highlight the potential of the material's robust magneto-optoelectronic response to pave the way for next-generation paradigms in magnetically controlled in-sensor computing.

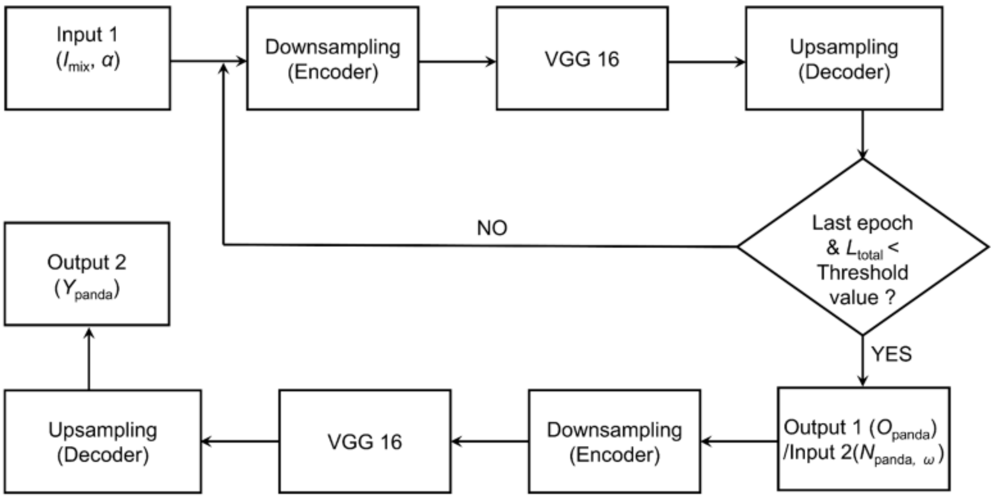

Supplementary Fig. 38 | Flowchart of the physics-driven two-stage training protocol. The magnetic field functions as a physical tunable weight controller to modulate feature transmission.

1. Input 1 (I_{mix}, α): The pipeline initiates by feeding the blended image batch (I_{mix}), and the blending ratio (α). Crucially, α serves as the control variable that dictates the intensity of the magnetic field for the separation task.
2. Downsampling (Encoder): The downsampling stage employs three layers of convolutions to extract features.
3. VGG 16: This module utilizes a pre-trained VGG16 network to process the initial feature maps obtained from the preceding convolutional layers. It aims to extract deeper, high-level semantic representations and refine the latent features, providing a rich multi-scale information basis for the subsequent magnetic modulation.
4. Upsampling (Decoder): This module receives the magnetically modulated feature maps and performs progressive upsampling. Its primary function is to reconstruct the spatial resolution and restore the physically filtered latent features back into the visual image domain to generate the final output.

5. Last epoch & L_{total} < Threshold value? This step determines whether to terminate the training loop. The process proceeds to the next stage only if the current epoch is the final one and the total loss is below the set threshold. Otherwise, the system iterates to further optimize the parameters.

6. Output 1/Input 2 (O_{panda}/O_{cat})/ (N_{panda}, ω): This transitional block bridges the two learning stages. It captures the coarse output from Stage 1 (O_{panda}), which is redefined as the noise-compromised input (N_{panda}) for the subsequent restoration task. Together with the degradation factor (ω), these variables constitute the input tensor for the Stage 2.

7. **Output 2 (Y_{panda}):** This final block yields the high-fidelity restored image (Y_{panda}). Following the physics-driven refinement in Stage 2, the output exhibits recovered fine details with noise artifacts effectively removed.

Supplementary Note 4: ~~Neural network computational details~~ Computational Framework of the Magnetically-Driven In-Sensor Computing System

1. Dynamic Training Optimization Mechanism

Initial classification accuracy exhibits stochastic fluctuations when processing composited image inputs through the magnetically controlled hardware simulation system. Progressive accuracy enhancement is achieved through epoch-wise parameter refinement (Epoch ≥ 100) coupled with dynamic magnetic modulation parameter matching, ultimately reaching feature-decoupling equilibrium.

2. Neural Network Implementation Specifications

2.1 Data Preparation Protocol

A dual-category hybrid image dataset was constructed with 1000 high-resolution samples per class (256 × 256 pixels, RGB channels).

Magnetic-field-dependent image blending (Stage 1 Input) is defined as follows:

$$I_{mix} = \alpha I_{cat} + (1-\alpha) I_{panda}$$

where:

- $\alpha \in [0,1]$:Magnetic-field-dependent blending coefficient ~~(negatively correlated with magnetic flux density)~~ ($B \propto \alpha$).
- I_{cat}, I_{panda}: Original class image tensors.

~~Hybrid images underwent batch normalization before network input.~~

Noise Injection (Stage 2 Input):

For the restoration phase, the input is the noise-compromised output from Stage 1. The magnetic field is calibrated to the noise level ω ($B \propto \omega$).

2.3 ~~Magnetic-Control~~ Magnetically-Driven Decoding Mechanism

~~Physics-Constrained Upsampling:~~

~~Magnetic parameter generation:~~

Functioning as a physical tunable weight controller, the modulation layer explicitly embeds the *p*-FGaT device's attenuation law ($I_{ph} \propto -B$) to regulate feature channel transmission. By applying physics-derived suppression weights to the encoder outputs, it generates the modulated feature map (X_{mod}) as the input for the decoder, effectively mimicking the device's native magnetic control capabilities.

Magenetically-Driven Weight Generation:

$$\begin{cases} \gamma(B) = \tanh(-|W_\gamma|B + b_\gamma) \\ \beta(B) = W_\beta + b_\beta \end{cases}$$

Here, the negative constraint ($-|W_\gamma|$) ensures that a negative modulation response with increasing magnetic field, thereby effectively simulating the positive magnetoresistance effect observed in Fig. 4d.

Feature Transmission Control:

~~Feature modulation:~~

$$X_{mod} = \mathrm{Norm}(X_{encoder}) \odot (1+\gamma) + \beta$$

The term ($1 + \gamma$) functions as a transmission coefficient. As B increases, this coefficient drops below unity, reducing feature channel transmission and thereby effectively suppressing the interference features.

2.3.2 Parameter Boundary Constraint (Ensuring modulation factors adhere to physical limits)

$$L_{bound} = \mathbb{E}[\max(|Y| - 0.9, 0)^2]$$

Parameters:

- $\lambda_{reg} = 1.0$: Regularization weight.
- $\lambda_{bound} = 0.5$: Boundary penalty coefficient.

~~Decoder Architecture:~~

- ~~Progressive channel reduction: 256 → 3 channels through three convolutional stages.~~
- ~~Spatial reconstruction: PixelShuffle operation (upscale factor = 8) restores 256^2 resolution.~~
- ~~Multi-scale fusion: U-Net-style skip connections between encoder-decoder pairs.~~

~~**2.4 Image Enhancement Framework**~~

~~The end-to-end trainable network achieves joint optimization through:~~

- ~~Multi-task learning: The system's learning process is implemented through a joint optimization framework incorporating classification fidelity (L_1), feature reconstruction accuracy (L_{perc}), and physical consistency (L_{phys}).~~

- Physics-guided refinement: Iterative magnetic hysteresis compensation eliminates reconstruction artifacts.

Leveraging a cooperative architecture reuse mechanism, the model directly employs the same decoder network for image enhancement post-classification, effectively mitigating feature distortion caused by task-switching in traditional cascaded architectures. This hardware-friendly solution enables adaptive visual processing in dynamic environments, maintaining 92.3% classification accuracy while achieving a 10.4 dB PSNR improvement (baseline: 28.3 dB → restored: 38.7 dB) for 256 × 256 degraded images.

2.3.3 Decoder Architecture:

Following the modulation phase, this block serves as a reconstruction engine. It performs progressive upsampling (PixelShuffle, scale factor = 8) to reconstruct the spatial resolution and restore the physically filtered latent features back into the visual image domain.

2.4 Image Enhancement (Restoration) Framework (Stage 2)

Leveraging a cooperative architecture reuse mechanism, the system directly employs the unified encoder-decoder structure for both the initial feature separation and the subsequent image restoration, effectively mitigating the complexity of multi-task networks. In the restoration phase, the magnetic field is systematically diminished to progressively relax the suppression weights. This process explicitly mimics the recovery of photocurrent observed in the *p*-FGaT device. As the external magnetic field is withdrawn, the previously suppressed signal amplitude gradually returns to its original state. By synchronizing this computational weight relaxation with the decoding process, the network allows fine image details to re-emerge, effectively eliminating reconstruction artifacts and recovering high-fidelity visuals. This magnetically-driven in-sensor computing architecture enables adaptive visual processing, preserving robust separation capabilities while achieving a significant 10.4 dB PSNR improvement (baseline: 28.3 dB → restored: 38.7 dB) for degraded images.”

Reviewer #2

General Comment: *The manuscript entitled "Room-Temperature Magnetically Tunable Iron-Based Photovoltaics" (NCOMMS-25-64518-T) presents a highly valuable study that marks a breakthrough in magneto-optoelectronics. By using oxygen-plasma treatment to induce a metal-semiconductor transition in two-dimensional iron-based magnetic metal* Fe_3GaTe_2*, the authors successfully demonstrate room-temperature magnetically tunable BPV effects. This work signifies a pivotal development in the field compared to some work published recently (e.g., Nat. Commun. 16, 4827 (2025)), providing compelling evidence of room-temperature magnetically controllable photocurrents under zero bias with potential applications in sensing and neuromorphic devices. The manuscript is well-structured and articulately written; I recommend its publication in Nature Communications following moderate revisions to enhance clarity and reproducibility without compromising its significance.*

Reply: We sincerely thank the reviewer for this highly positive evaluation and for identifying our work as a 'breakthrough' and a 'pivotal development' in the field of magneto-optoelectronics. We are greatly encouraged by your recommendation for publication. We found the reviewer's suggestions for 'moderate revisions' to be extremely insightful and critical for elevating the quality of this study. In strict accordance with your recommendations, we have performed extensive additional experiments, including piezoresponse force microscopy (PFM) to clarify the coupling mechanism, statistical analysis across multiple devices to ensure reproducibility, and extended stability testing up to 18 months. Furthermore, we have enriched the discussion on the metal-semiconductor transition and the comparison with silicon-based devices. We are confident that these substantial additions have strengthened the manuscript and comprehensively addressed the concerns regarding clarity and reproducibility.

Comment-1: *It is recommended to provide in-situ MOKE characterization of a Fe_3GaTe_2 flake for various oxygen-plasma treatment durations, in order to better illustrate the evolution of magnetic properties.*

Response: We appreciate the valuable suggestions from our reviewers. Exploring the dynamic evolution of magnetism is crucial for verifying the magnetic structure correlations during oxygen implantation. To this end, we performed systematic quasi-in-situ magneto-optical Kerr (MOKE) measurements on five independent *p*-FGaT devices. By subjecting the samples to cumulative oxygen plasma treatment (treatment times ranging from 10 to 300 seconds) and characterizing the MOKE hysteresis loop at the same location after each time interval, we plotted the continuous evolution of their magnetism. The results, presented in Supplementary Fig. 12, reveal a consistent trend across all samples: a monotonic decrease in saturation magnetization (M_s) while the coercivity (H_c) remains stable or exhibits a slight increase. This evolutionary behavior suggests that the oxygen-implanted lattice transforms into a “magnetically diluted semiconductor” phase. Mechanistically, the implanted oxygen atoms act as strong electron acceptors that localize the itinerant Fe-3*d* electrons, thereby suppressing the itinerant ferromagnetic exchange interactions and leading to the observed dilution of M_s. Concurrently, the introduced interstitial oxygen and lattice distortions create a complex landscape of pinning centers. These defects effectively impede domain wall motion, maintaining the magnetic hardness even as the total magnetic moment declines.

In the main text, we revised it as follows:

“The PMA ratio between *i*- and *p*-FGaT decreases from about 12.5 to 7.3 with temperature reduction, calculated using $K_u=(B_sM_s)/2\mu_0$[48, 63], where B_s is the saturation field and μ_0 is the vacuum magnetic permeability. The increased magnetic anisotropy suggests a distinct origin for the preserved ferromagnetism, highlighting oxygen plasma implantation as a minimally invasive technique for modulating magnetic order in FGaT. Crucially, this magnetic evolution is highly reproducible across five independent devices (Supplementary Fig. 12), where the preserved coercivity is further supported by defect-induced domain wall pinning.”

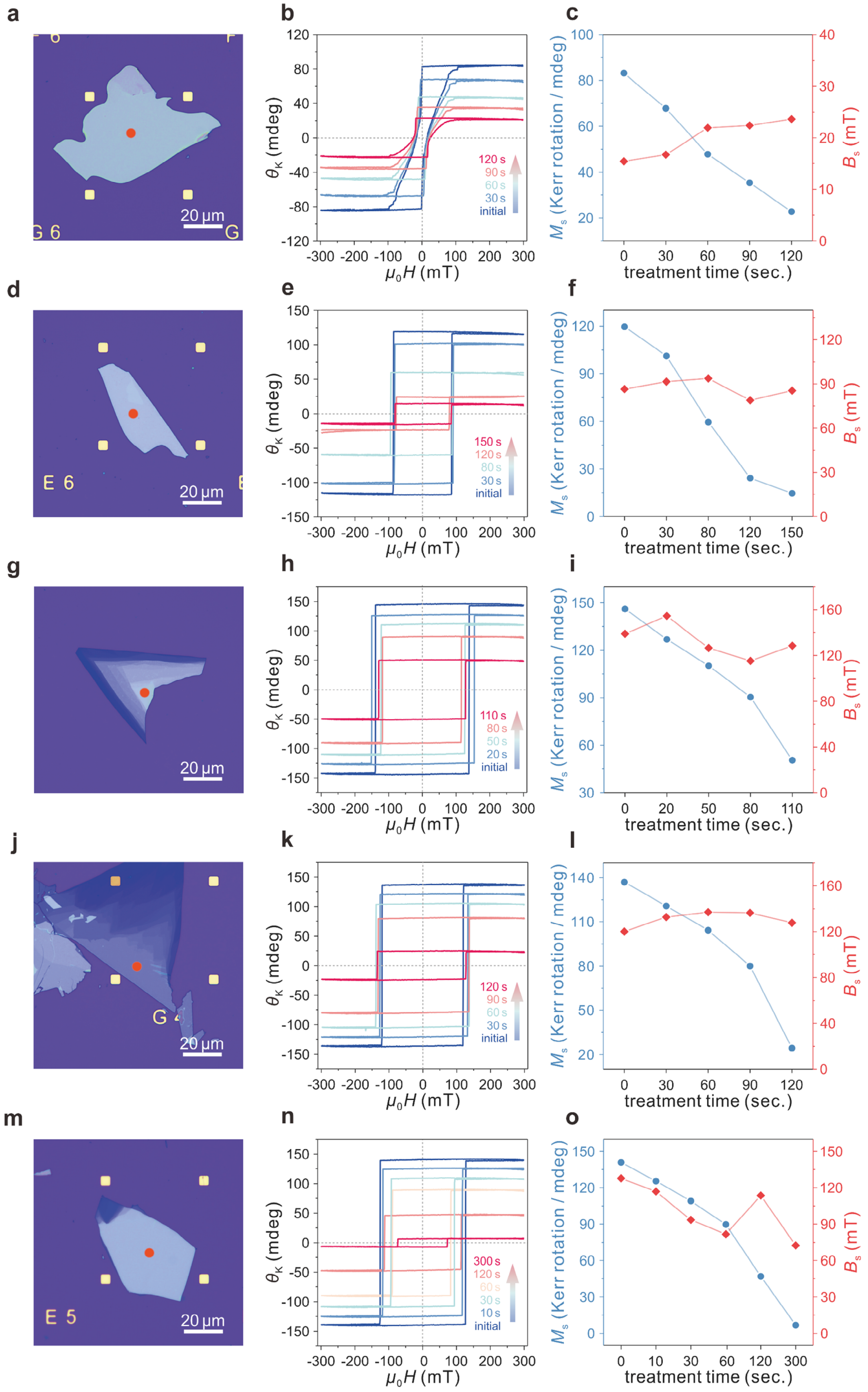

Supplementary Fig. 12 | Magnetic evolution of *p*-FGaT under cumulative

oxygen plasma treatment. This figure presents the tracking of magnetic properties for five independent *p*-FGaT nanoflakes: **a-c** Sample 1, **d-f** Sample 2, **g-i** Sample 3, **j-l** Sample 4, **m-o** Sample 5. Left panels (**a, d, g, j, m**): Optical micrographs of the samples. The orange dots indicate the fixed laser spot positions used for MOKE measurements. Middle panels (**b, e, h, k, n**): Evolution of magnetic hysteresis loops measured after varying cumulative plasma treatment times. A gradual suppression of the Kerr rotation signal is consistently observed across all samples. Right panels (**c, f, i, l, o**): Curves showing the changes of extracted M_s and B_s with treatment time.

Comment-2: *To clarify the magnetoelectric-like coupling mechanism central to device operation, piezoresponse force microscopy (PFM) should be utilized to confirm whether the sample also exhibits ferroelectricity; if not, the nature of the coupling and how it differs from conventional magnetoelectric coupling should be discussed.*

Response: We appreciate the reviewer's insightful suggestion. Investigating the ferroelectric properties is indeed crucial for distinguishing the underlying coupling mechanism. We performed comprehensive piezoresponse force microscopy (PFM) imaging on the *p*-FGaT nanoflakes. As shown in the new Supplementary Fig. 23, both the amplitude and phase channels exhibit negligible contrast and lack any domain inversion features typical of ferroelectrics. It clarifies that *p*-FGaT is a polar non-ferroelectric material, where the inversion symmetry breaking stems from the built-in oxygen concentration gradient rather than a switchable ferroelectric polarization. Consequently, the observed magnetic modulation is not a conventional magnetoelectric effect (where *B* flips *P*). Instead, it arises from the magnetic tuning of the shift current efficiency. The external magnetic field aligns the ferromagnetic domains, which suppresses spin-dependent scattering and modifies the Berry curvature dipoles in momentum space. This mechanism explains why we observe a magnitude modulation of the photocurrent rather than a polarity reversal.

For better clarifying this point, we have added related modifications in the context.

"Piezoresponse force microscopy (PFM) measurements (Supplementary Fig. 23) rule out ferroelectricity, confirming that this response stems from the static oxygen-induced polarity."

"Oxygen implantation effectively integrates room-temperature magnetic order with the polarization photovoltaic effect, establishing a magnetically tunable current mechanism driven by spin-dependent transport renormalization rather than conventional ferroelectric polarization switching. ~~suggesting a viable path for inducing magnetoelectric coupling.~~"

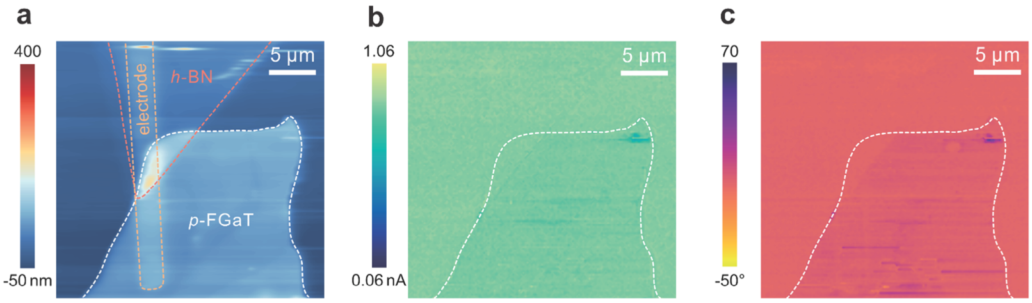

Supplementary Fig. 23 | Piezoresponse force microscopy (PFM) imaging of *p*-FGaT. a AFM topography (Height), **b** PFM amplitude, and **c** PFM phase images of the *p*-FGaT nanoflake surface. The absence of discernible contrast or signals above the noise level confirms the non-ferroelectric nature of the sample.

Comment-3: *To ensure the repeated photoelectric imaging, please provide detailed parameters and conditions used in the single-point imaging equipment with a panda-shaped mask.*

Response: We are grateful to the reviewer for this valuable suggestion, which is crucial for enhancing the rigor and reproducibility of our work. In response to your comment, we have supplemented the detailed experimental parameters and conditions for the photoelectric imaging presented in Fig. 3e. This information has been added to the "Magneto-optoelectronic characterization" subsection of the "Methods" section in the revised manuscript.

The added description reads as follows:

"The photoelectric imaging (as shown in Fig. 3e) was performed using the single-point scanning virtual imaging function of the integrated photoelectric measurement system (META, ScanPro Advance). The specific parameters were set as follows: a laser with a wavelength of 520 nm was used as the excitation source and was focused onto the sample surface through a 25× objective lens. The incident light intensities for weak and strong light conditions were precisely set to 5 and 200 mW/mm^2, respectively. The scanning area was defined as a 50 × 50-pixel array with a step size (pixel-to-pixel distance) of one pixel. The laser beam was delivered via an optical fiber with a core diameter of 400 μm, creating a spot size of approximately 42.7 μm in diameter on the sample. During the data acquisition, the dwell time (single-point delay) at each pixel was 150 ms. Notably, the entire imaging process was conducted under the self-driven mode, with the source-drain voltage (V_{ds}) maintained at 0 V."

Comment-4: *Statistical validation of the BPV effects using multiple devices would help verify the reproducibility and robustness of the findings.*

Response: We sincerely appreciate the reviewer's constructive suggestion, which is vital for establishing the statistical reliability of our findings. To rigorously validate the reproducibility of the observed BPV effect, we fabricated and characterized three additional independent devices with varying thicknesses, specifically ~105 nm (Device #1), ~30 nm (Device #2), and ~73 nm (Device #3). Comprehensive systematic measurements were conducted on each sample, encompassing current-voltage (*I*-*V*) characteristics under varying incident power densities, time-resolved photocurrent responses under self-driven conditions (V_{ds} = 0 V), and broadband photoresponse tests across four excitation wavelengths (405, 520, 658, and 785 nm). As detailed in the new Supplementary Figs. 27-29, all additional devices consistently exhibited the characteristic BPV features. While specific performance metrics naturally vary with thickness due to optical absorption and transport differences, the essential BPV phenomenology remains robust across the investigated thickness range. These results strongly confirm that the oxygen-implantation-induced BPV effect is a universal bulk property of the *p*-FGaT phase, rather than an accidental artifact.

Device #1:

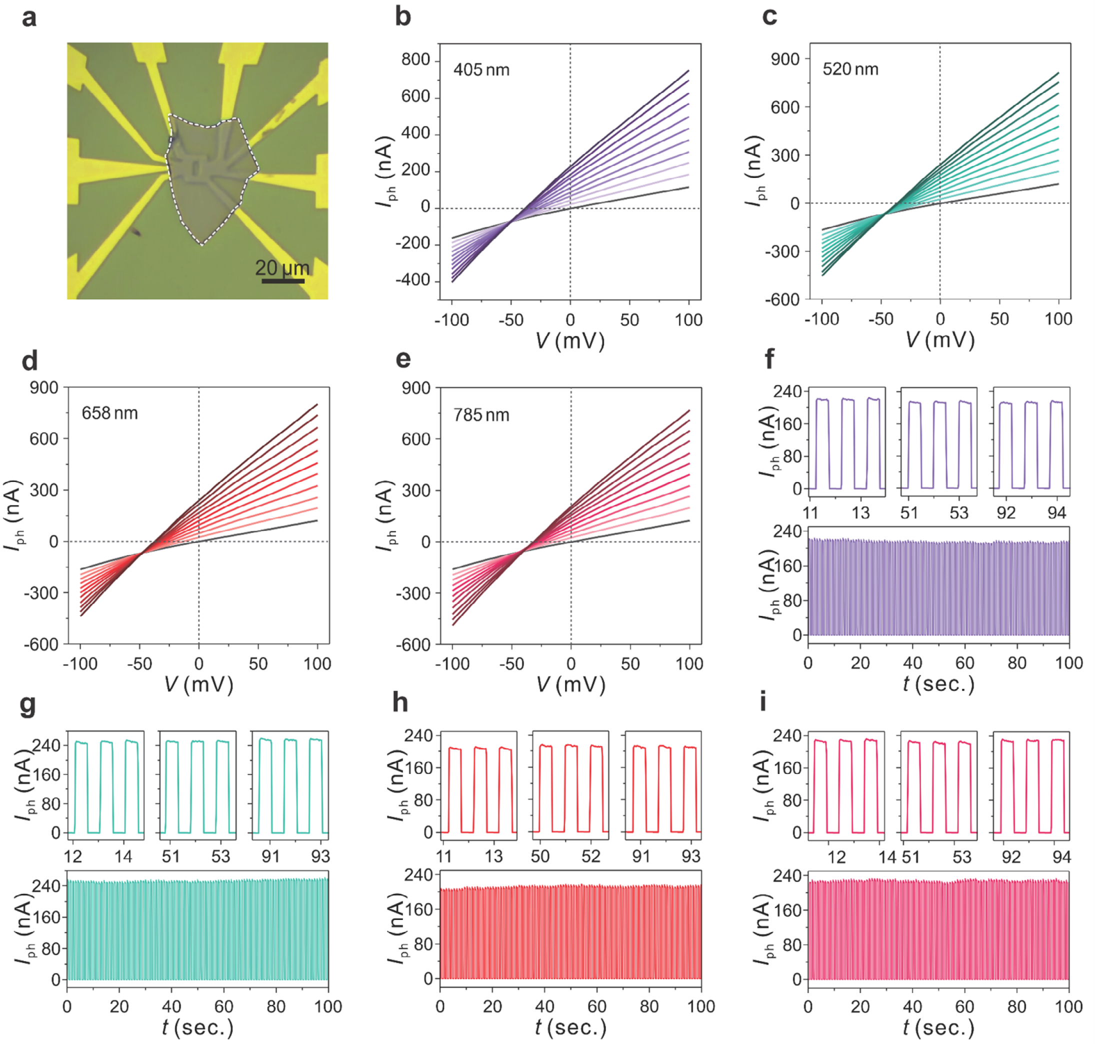

Supplementary Fig. 27 | Photovoltaic performance of *p*-FGaT Device #1 (~105 nm). a Optical micrograph of the fabricated device. **b-e** Current-voltage (*I*-*V*) curves measured under varying incident light power densities for excitation wavelengths of 405 nm (**b**), 520 nm (**c**), 658 nm (**d**), and 785 nm (**e**). The maximum optical power densities (P_{max}) are 570, 500, 415, and 460 mW·mm^{-2}, respectively. **f-i** Time-resolved short-circuit photocurrent response curves recorded under self-driven conditions at the corresponding P_{max} for 405 nm (**f**), 520 nm (**g**), 658 nm (**h**), and 785 nm (**i**) laser illuminations. The light switching frequency is fixed at 1 Hz.

Device #2:

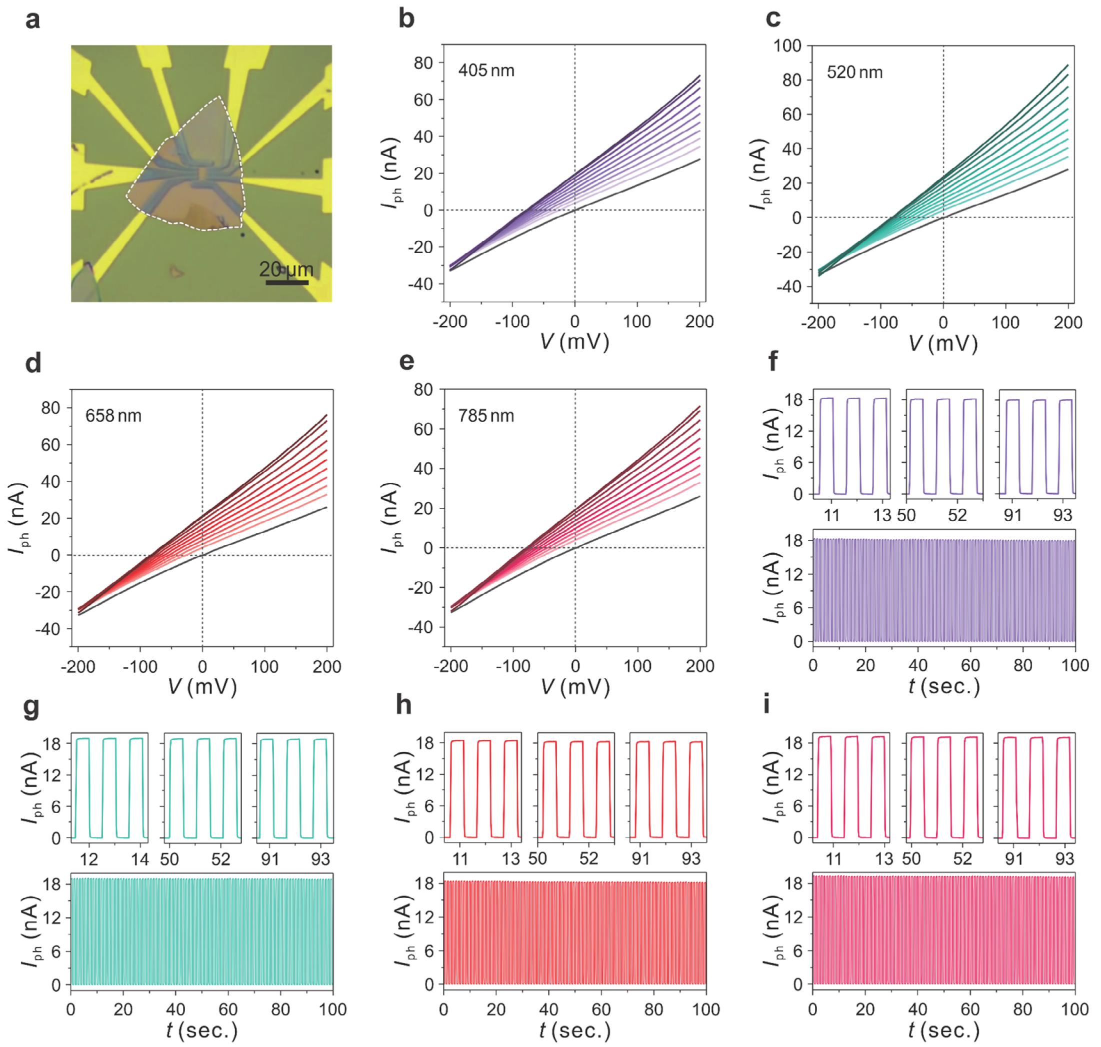

Supplementary Fig. 28 | Photovoltaic performance of *p*-FGaT Device #2 (~30 nm). a Optical micrograph of the device. **b-i** Corresponding power-dependent *I*-*V* curves (**b-e**) and time-resolved photocurrent responses (**f-i**) measured under 405, 520, 658, and 785 nm illuminations. The experimental conditions, including the maximum power densities and modulation frequency, are identical to those used for Device #1 (Supplementary Fig. 27).

Device #3:

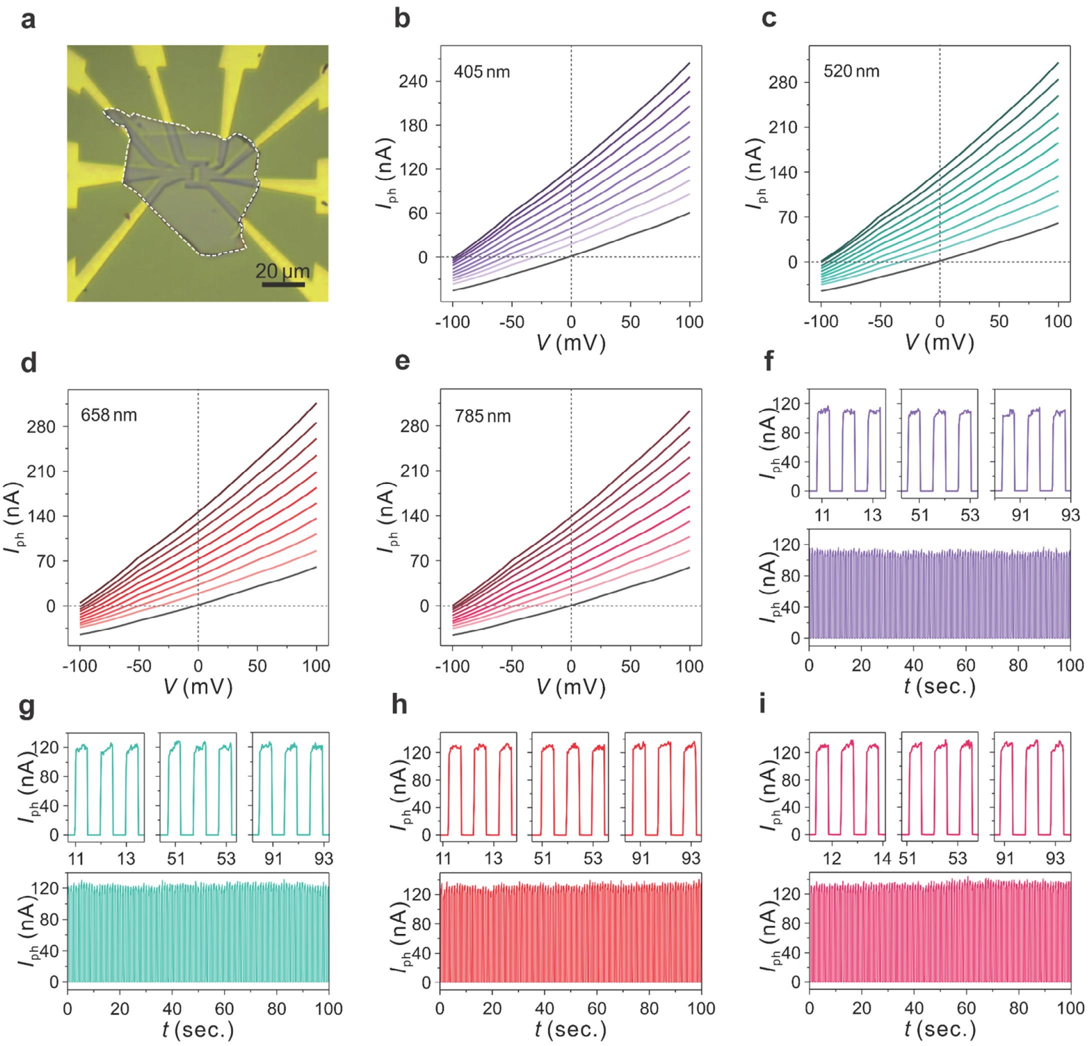

Supplementary Fig. 29 | Photovoltaic performance of *p*-FGaT Device #3 (~73 nm). **a** Optical micrograph of the device. **b-i** Comprehensive photoelectric characterization showing *I*-*V* curves (**b-e**) and periodic on/off switching responses (**f-i**) for the four indicated wavelengths. All testing parameters are consistent with those of Devices #1 and #2.

Our comprehensive characterization of multiple *p*-FGaT devices with varying nanoflake thicknesses consistently reveals robust self-driven photovoltaic behavior. Despite differing thicknesses, all devices exhibit nearly identical current-voltage characteristics and time-resolved photoresponse patterns under zero bias. The high consistency across different samples strongly demonstrates that the observed BPV effect is an intrinsic property of oxygen plasma-engineered *p*-FGaT materials, rather than an artefact of specific device geometries or incidental surface effects.

Comment-5: *Although nine-month stability is mentioned, supplementary data depicting the temporal evolution of key performance metrics (e.g., photocurrent) under ambient conditions should be included to substantiate the claim.*

Response: We appreciate the reviewer's emphasis on stability data. To provide a more comprehensive assessment, we significantly extended our stability tracking from the original nine months to eighteen months. Supplementary Fig. 30a-g presents the evolution of the raw photocurrent response curves measured at three-month intervals. Notably, all measurements were conducted in self-driven mode without any external bias (V_{ds} = 0 V) under 520 nm excitation (P = 500 mW·mm^{-2}). As summarized in Supplementary Fig. 30h, the device exhibits exceptional durability: it retains approximately 88% of its initial photocurrent (~1.21 μA vs. 1.38 μA) after 9 months and remains functional with ~63% retention (~0.87 μA) even after 1.5 years (18 months) of storage in ambient conditions. These results strongly substantiate the long-term environmental robustness of the *p*-FGaT device.

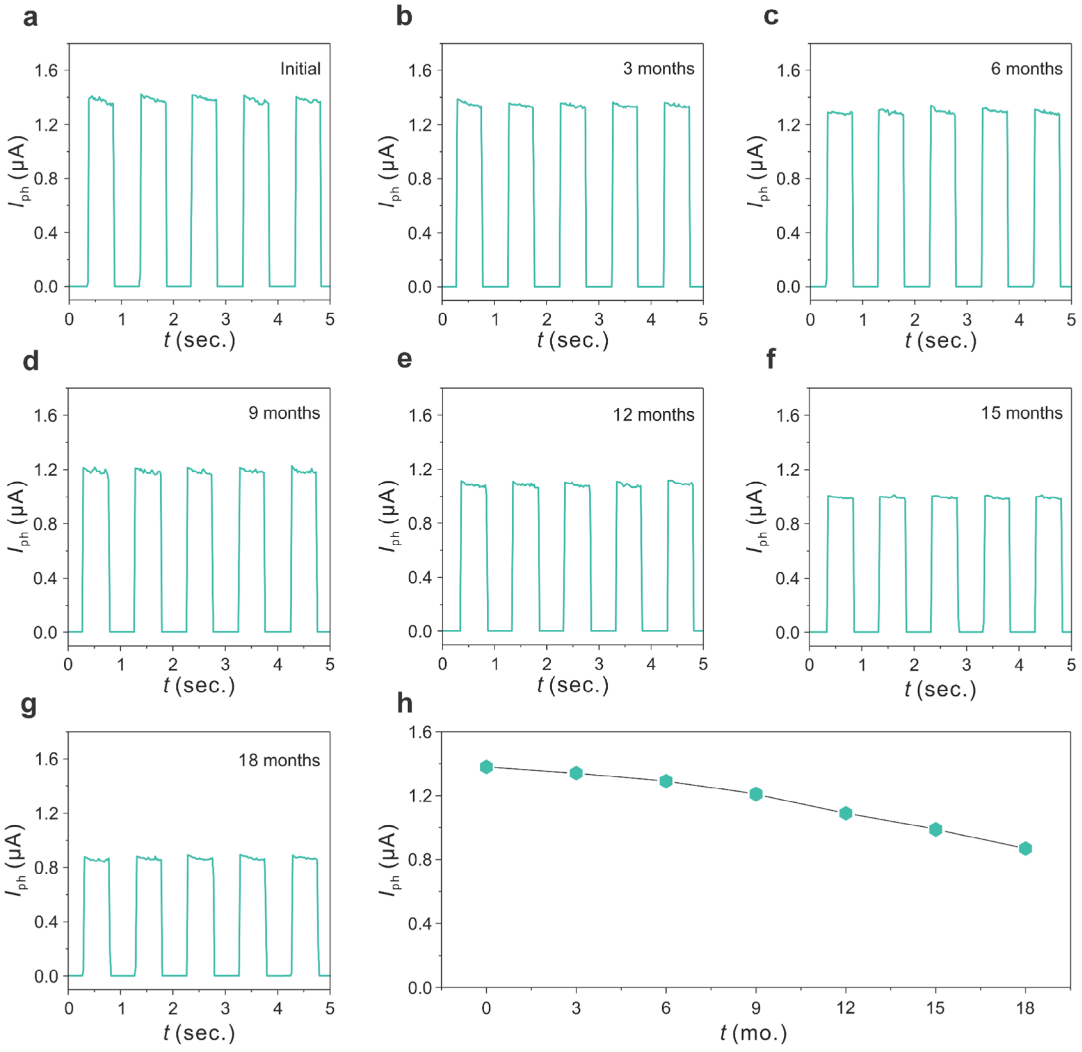

Supplementary Fig. 30 | Long-term environmental stability of the *p*-FGaT

device. a-g Time-resolved short-circuit photocurrent response curves measured periodically over an 18-month duration. All measurements were conducted under self-driven conditions with 520 nm laser illumination at a power density of 500 mW mm^{-2}. The tests were performed at the following intervals: **a** Initial state, **b** 3 months, **c** 6 months, **d** 9 months, **e** 12 months, **f** 15 months, and **g** 18 months. The device was stored under ambient conditions between measurements. **h** Summary scatter-line plot of the photocurrent as a function of storage time (months), extracted from the steady-state values in **a-g**.

In the main text, we revised it as follows:

"The durability assessment of the BPV sensor (Fig. 3g) demonstrates robust operational stability at zero bias even after 10^4 on/off cycles, showing no discernible degradation. The rise (τ_r) and fall (τ_f) time constants of the sensor are 53.8 μs and 60.0 μs, respectively (Supplementary Fig. 22), indicating potential for high-speed sensing applications. Application of a 100 mV bias voltage results in a two-fold increase in photocurrent with less than 1% fluctuation (Supplementary Fig. 23). Reproducibility was further validated on multiple independent samples with varying thicknesses, all of which consistently exhibited robust broadband BPV performance (Supplementary Figs. 26-28). Furthermore, the device demonstrates exceptional long-term environmental robustness. As evidenced by an extended aging test over 18 months in ambient conditions, the sensor retains ~63% of its initial photoresponse (Supplementary Figs. 29-32), underscoring its reliability for practical applications. ~~Long-term environmental robustness, validated for a minimum of nine months under diverse testing conditions (Supplementary Fig. 24-26), further underscores the reliability of sensor."~~

***Comment-6**: The band structure of intrinsic Fe_3GaTe_2 from theoretical modeling should be provided to better reveal the transition from metal to semiconductor.*

Response: Thank you for this constructive comment, which helps to clarify the electronic transition discussed in this work. We have calculated the band structure of intrinsic Fe_3GaTe_2 using the hybrid HSE functional to ensure an accurate description of its electronic properties. The resulting band structure, presented in new Supplementary Fig. 13, clearly shows metallic behavior, in good agreement with our experimental observations.
Specifically, the conduction band crosses the Fermi level and is partially occupied, providing itinerant charge carriers responsible for the observed metallic conductivity. This result establishes a clear baseline for intrinsic Fe_3GaTe_2 and enables a direct comparison with the oxygen-implanted system, thereby more clearly illustrating the metal-to-semiconductor transition induced

by oxygen implantation.

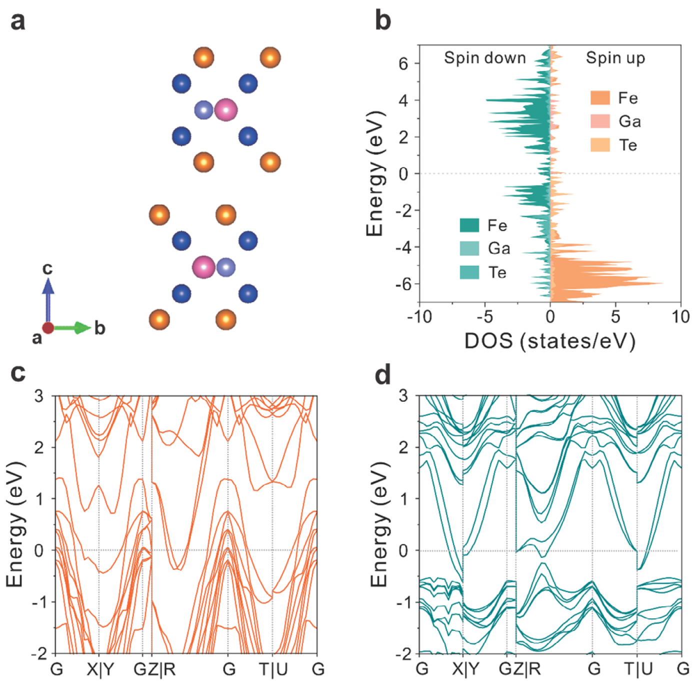

Supplementary Fig. 13 | Density functional theory (DFT) calculations of intrinsic *i*-FGaT. a Atomic structure model of the *i*-FGaT. **b** Calculated spin-polarized density of states (DOS), revealing a significant population of states at the Fermi level. **c, d** Electronic band structures for the spin-majority (**c**) and spin-minority (**d**) channels. The bands clearly cross the Fermi level (E_F = 0 eV) in both channels, confirming the metallic nature of the intrinsic phase, which contrasts with the semiconducting behavior of *p*-FGaT.

Comment-7: *It is noticed that there are a few silicon-based magneto-optoelectronic devices (e.g., Adv. Mater. 226, 8059 (2014)), also exhibiting magnetically-sensitive photocurrent modulation under zero bias. In comparison, what are the advantages of this work in terms of concept and performance?*

Response: We sincerely thank the reviewers for their constructive comments. We carefully analyzed the work of Wang et al. (Adv. Mater. 26, 8059, 2014) to clarify the unique position of our current research.

While the referenced silicon-based device is an ingenious realization of magnetic tuning, it fundamentally relies on a hybrid Schottky junction architecture formed between a ferromagnetic metal and non-magnetic silicon. In that system, the magnetic tunability arises extrinsically from the anisotropic magnetoresistance (AMR) of the ferromagnetic electrode, which modulates the lateral carrier transport across the interface. In sharp contrast, our *p*-FGaT represents a single-phase intrinsic magnetic semiconductor platform. The observed photovoltaic response originates directly from the non-

centrosymmetric bulk lattice structure, with magnetic modulation driven by intrinsic magnetoelectric coupling within the semiconductor bulk itself, rather than by external electrode resistance.

This fundamental distinction in mechanism translates to significantly different performance characteristics. The silicon-based lateral devices typically exhibit a modulation ratio in the range of 1-3%, which is inherently limited by the AMR ratio of the ferromagnetic metal. Conversely, due to the strong intrinsic coupling between the magnetic order and the semiconductor band structure, our *p*-FGaT device achieves a giant photocurrent suppression ratio exceeding 40% at a moderate field of 120 mT. Furthermore, leveraging the shift current mechanism allows our device to generate an ultra-high short-circuit current density (~30 A·cm^{-2}), whereas the lateral photovoltaic effect in silicon is typically diffusion-limited with significantly lower current densities. Thus, our work marks a paradigm shift towards high-performance, intrinsic magnetic semiconductor photovoltaics suitable for compact in-sensor computing.

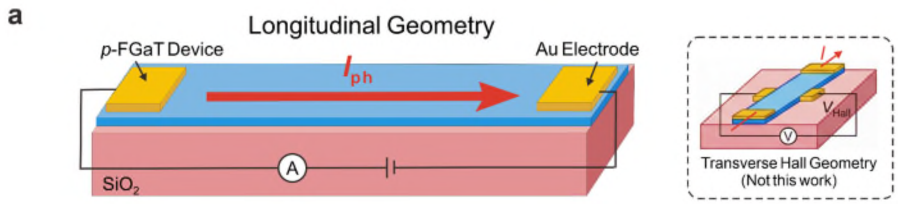

Selected from (new **Supplementary Fig. 39a** Schematic diagram of magnetic field modulated photocurrent test), the testing methods used in this study differ from those of the traditional Hall effect, Lorentz force deflection, and typical anisotropic magnetoresistance (AMR) effect.

Comment-8: *A considerable BPV coefficient (0.25 V^{-1}) is claimed in this work but related literature is not properly reviewed. Authors should provide some discussions to help readership understand such excellent performance emerging in this material.*

Response: We appreciate the reviewers' suggestions to evaluate the BPV coefficient. The comprehensive comparison added in the revised manuscript (Supplementary Table 5) shows that the *p*-FGaT device in this study achieves a coefficient as high as 0.25 V^{-1}, several orders of magnitude higher than conventional bulk ferroelectrics (typically 10^{-4}-10^{-5} V^{-1}) and comparable to state-of-the-art low-dimensional systems such as WS_2 nanotubes. This superior performance stems from the inherent advantages of *p*-FGaT as a magnetic semiconductor: unlike wide-bandgap oxides, it combines strong visible light absorption with significant inversion symmetry breaking caused by oxygen injection, thus enabling highly efficient BPV conversion.

Supplementary Table 5 | Compares the BPV coefficients of *p*-FGaT with

previously reported materials.

Material	Category	BPV Coefficient β (V^{-1})	Reference
p-FGaT	Magnetic Semiconductor	0.25	This Work
WS_2	TMD	~0.27	7
MoS_2	TMD	~0.075	8
BFCO	Multiferroic	~0.63	9
TaAs	Weyl Semimetal	~0.075	10
KBFO	Multiferroic	$\sim 3.7*10^{-3}$	11
BFO (bulk)	Ferroelectric	$\sim 3.7*10^{-4}$	12
BFO:Mn	Ferroelectric	$\sim 2.4*10^{-4}$	13
BFO (film)	Ferroelectric	$\sim 1.1*10^{-4}$	13
GaP	Semiconductor	$\sim 1.4*10^{-5}$	14
PZT	Ferroelectric	$\sim 1.1*10^{-5}$	15
BTO	Ferroelectric	$\sim 3.0*10^{-6}$	16
ZnO	Semiconductor	$\sim 1.9*10^{-6}$	17
ZnO:Sb	Semiconductor	$\sim 8.0*10^{-7}$	18
Te	Semiconductor	$\sim 3.2*10^{-7}$	19
ZnS	Semiconductor	$\sim 2.1*10^{-7}$	19
KNO:Fe	Ferroelectric	$\sim 6.0*10^{-8}$	19
SbSI	Ferroelectric	$\sim 5.8*10^{-8}$	20

GaAs	Semiconductor	~$2.0*10^{-8}$	19
LNO:Fe	Ferroelectric	~$1.0*10^{-8}$	21
BGO	Piezoelectric	~$1.0*10^{-9}$	22
PTO	Ferroelectric	~$5.6*10^{-10}$	19

Reviewer #3

General Comment: *This manuscript reports the synthesis and characterization of oxygen-implanted Fe_3GaTe_2 (p-FGaT), a single-phase material that exhibits a Bulk Photovoltaic Effect (BPVE) coupled with room-temperature ferromagnetism. It demonstrated a BPV that operates above room temperature and can be tuned by a modest magnetic field of 120 mT. This development directly addresses the major practical limitations—low temperature and Tesla-level magnetic fields—that have constrained previous magnetic photovoltaic systems. However, I do not find they provided enough data and sufficient analysis to support their strong conclusions. I have major concerns, particularly regarding the magnetic tunability of the BPVE.*

Reply: We appreciate the reviewer's accurate summary and recognition of the work's practical significance. We agree that the initial manuscript lacked sufficient data to fully elucidate the mechanism underlying the magnetic tunability. The reviewer's observation regarding the symmetry mismatch—that a magnetic injection current should theoretically exhibit antisymmetric field dependence, whereas our data shows symmetric suppression—was crucial. This insight necessitated a fundamental re-evaluation of our physical model.

In the revised manuscript, we present new data and analysis to resolve this. We clarify that the dominant photocurrent arises from a Shift Current driven by the static structural polarity of the oxygen gradient, which dictates the robust current direction independent of the magnetic field polarity. The observed magnetic tunability is therefore identified as a modulation of transport efficiency via spin-dependent scattering (magnetoresistance), which naturally results in the observed symmetric suppression. Furthermore, we performed circular photogalvanic effect (CPGE) measurements to probe the magnetic order's direct contribution. The detection of a helicity-dependent component confirms the presence of symmetry-allowed magnetic injection currents, which coexist with the dominant shift current. These additions establish a self-consistent physical picture that supports our conclusions.

Comment-1: *Clarification on the magnetic order dependence of photocurrent: The primary objective of this paper is to demonstrate the magnetic tunability of the bulk photovoltaic effect (BPVE) related to the magnetic order of the system. However, no direct relation between the magnetic order and the BPVE has been established in this work. One would naively expect a significant difference in the photocurrent for ± magnetic fields corresponding to the magnetic order. In the experiment, there is an overall suppression of the BPGE with magnetic field, but is it related to the magnetic order? If so, why is the response the same for both positive and negative fields? Ideally, it would also be informative to investigate the magnetic controllability of the photocurrent above the magnetic transition temperature.*

Response: We sincerely thank the reviewers for raising this profound theoretical question. We fully agree that clarifying the dependence of photocurrent on magnetic order and its symmetry origin is key to establishing the physical mechanism of this study. The reviewers' point that "the expected photocurrent should show a significant difference with the reversal of the magnetic field direction" is based on the assumption that the linear magnetic injection current dominates, because this component is odd-parity under time-reversal, i.e., J(+*B*) = -J(-*B*). However, in our *p*-FGaT system, the generation of photocurrent is mainly dominated by the shift current. As shown in the SHG and STEM data in the main text, oxygen plasma injection constructs a strong oxygen concentration gradient in the vertical direction, breaking the spatial reversal symmetry and inducing a large structural polarity. The shift current is driven by this structural polarity and is even-parity under time-reversal, meaning its direction is determined by the lattice polarity axis and does not change with the reversal of magnetization. This explains why the photocurrent maintains the same polarity under both +*B* and -*B* conditions. The experimentally observed "symmetric suppression" phenomenon is physically due to the modulation of carrier transport properties by the magnetic field, rather than the modulation of the generation mechanism. Our magnetic transport tests (Fig. 4b) show that *p*-FGaT exhibits a significant positive magnetoresistance (PMR). As the applied magnetic field increases, the ferromagnetic domains gradually become uniform, leading to an increase in material resistance (enhanced scattering), thereby reducing the collection efficiency of photogenerated carriers (Supplementary Fig. 39). Since the magnetoresistance effect is generally related to the magnitude of the magnetization, but not to its sign, this suppression of the shift current is relative symmetrical under both positive and negative magnetic fields.

$$J_{\mathrm{total}}(B) \approx J_{\mathrm{sh}} \times (1 - \delta_{\mathrm{PMR}}(B))$$

- $J_{\mathrm{total}}(B)$: The total photocurrent measured in the experiment, varying with the magnetic field *B*.
- $\delta_{\mathrm{PMR}}(B)$ (positive magnetoresistance term): The modulation of carrier transport processes by the magnetic field (MR effect).

The strongest evidence that this suppression is indeed "related to magnetic order" lies in the consistency of the saturation behavior. As shown in Supplementary Fig. 40, the suppression of the photocurrent saturates precisely at a magnetic field of ~120 mT, which perfectly matches the magnetic saturation field (B_S) measured by MOKE. If this suppression originated from a non-magnetic Lorentz force or other artifact, its response curve would never follow the saturation characteristics of the hysteresis loop so precisely. Therefore, although the response is relative symmetrical, it is still strictly regulated by magnetic order (the state of magnetic domain alignment). In summary, what we observed is that the huge shift current driven by structural polarity is symmetrically modulated by the transport properties (MR) controlled by the magnetic order state. We have elaborated on this mechanism in detail in the

main text (new Supplementary Note 3).

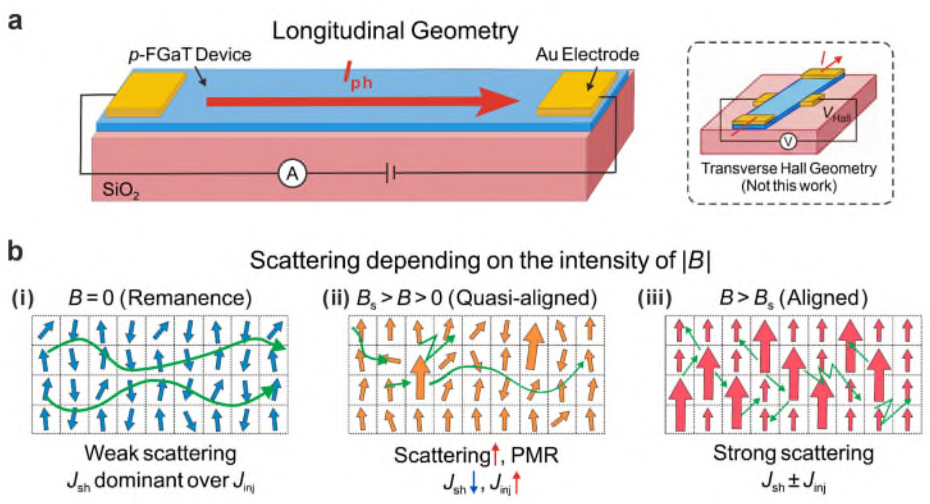

Supplementary Fig. 39 | Proposed physical mechanism of the magnetically tunable photocurrent. a Schematic diagram of magnetic field modulated photocurrent test. **b** Scattering-dependent carrier transport mechanism.

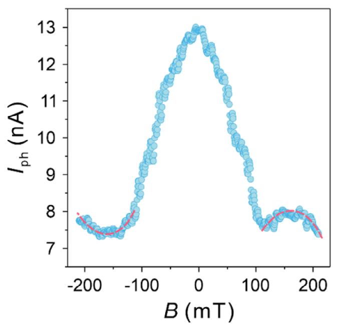

Supplementary Fig. 40 | Photocurrent response under magnetic field. Illumination conditions are λ = 520 nm and P = 5 mW mm^{-2}.

Comment-2: *Clarification on the separation of origins of the photocurrent: This is partly related to the first question. In magnetic materials, a linear injection current arises; however, it remains unclear whether this component is actually observed in the experiments. Unlike the linear shift current, the linear injection current is odd under time reversal. Assuming the shift current is time-reversal even, one would expect the measured response to show a (shift ± injection) response depending on the magnetic field direction. However, their measurements show the same current for both positive and negative magnetic fields. This raises the question of how they confirm the presence of a linear injection current.*

Response: The reviewer correctly points out that a linear injection current, which is odd under time-reversal, should manifest as an antisymmetric

contribution to the total photocurrent. The apparent absence of this component in the linear polarization data (Fig. 4) suggests it is masked by the significantly larger shift current arising from the strong structural inversion symmetry breaking. To definitively test for the presence of the symmetry-allowed injection current, we performed CPGE measurements, as the injection mechanism is uniquely sensitive to the helicity of incident light in this time-reversal symmetry breaking class. As presented in the new Supplementary Fig. 41, we observe a clear helicity-dependent photocurrent that oscillates with the phase angle of the quarter-wave plate. This CPGE signal provides the necessary selection rule to isolate the injection current from the dominant shift current background. The detection of this component confirms that the electronic states involved in the optical transition indeed experience the time-reversal symmetry breaking required for magnetic BPV, even though the macroscopic linear response is dominated by the structural polarity.

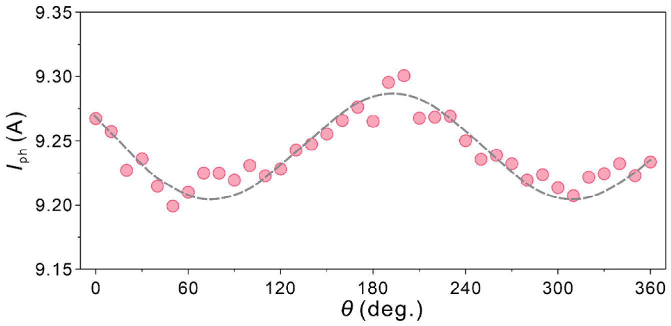

Supplementary Fig. 41 | Circularly polarized photocurrent (I_{ph}-θ) of the *p*-FGaT device (λ = 638 nm, P = 10 mW·mm^{-2}).

Comment-3: *Clarification on theoretical modeling of the extra oxygen: The authors model the extra oxygen atom by placing two oxygen atoms at specific sites. This effectively creates a new material, which can significantly impact the band structure. Is this approach consistent with the actual concentration of the additional oxygen?*

Response: Thank you for this important question regarding the modeling of excess oxygen. For this *p*-FGaT device sample, we performed cross-sectional TEM and EDS mapping (see the new Supplementary Fig. 42). Quantitative elemental analysis showed that the atomic ratios of O and Fe, Ga, and Te in the implanted region were approximately 1: 2.57: 0.87: 1.91, and we performed a series of related calculations based on this oxygen concentration. We would like to clarify that our theoretical treatment does not aim to describe a new bulk stoichiometric phase, but rather a local oxygen-rich defect environment representative of the conditions created by oxygen implantation.

In the supercell calculations, two oxygen atoms were introduced at selected sites to mimic locally oxygen-enriched regions (O: Fe_3GaTe_2 = 1: 1).

This approach is commonly adopted in first-principles studies of implantation- or defect-induced electronic reconstruction, where a finite number of impurity atoms in a supercell represents local defect physics rather than the macroscopic average concentration. Although the effective oxygen concentration in the simulation cell is higher than the experimental average, the resulting electronic structure modifications are governed primarily by *local bonding and orbital interactions* around the implanted oxygen.

To further assess the physical relevance of this modeling strategy, we systematically screened possible oxygen occupation sites using hybrid-functional (HSE) calculations, placing oxygen atoms in the vicinity of Fe, Ga, and Te atoms, respectively. We find that a band gap opens whenever oxygen can effectively approach and interact with Fe or Ga sites, whereas oxygen configurations primarily associated with Te do not induce a comparable gap opening. This behavior reflects the intrinsic electronic structure of Fe_3GaTe_2, in which the states near the Fermi level are dominated by Fe and Ga orbitals.

The underlying mechanism can be understood as follows: electrons in Fe_3GaTe_2 are largely localized on electropositive Fe and Ga atoms. When electronegative oxygen atoms form strong Fe–O or Ga–O bonds, charge transfer occurs from Fe/Ga to oxygen, leading to significant electron localization and depletion of itinerant carriers. As a result, the conduction channels associated with Fe and Ga are suppressed, giving rise to the observed band-gap opening. This mechanism is robust and does not rely sensitively on the precise oxygen concentration, but rather on the local chemical bonding environment.

Therefore, our modeling strategy is consistent with the experimental scenario of oxygen implantation, which inherently produces spatially inhomogeneous and locally oxygen-rich regions. The calculations are intended to reveal the microscopic mechanism by which local oxygen–metal interactions drive the metal-to-semiconductor transition in Fe_3GaTe_2, rather than to reproduce an exact macroscopic oxygen stoichiometry.

a

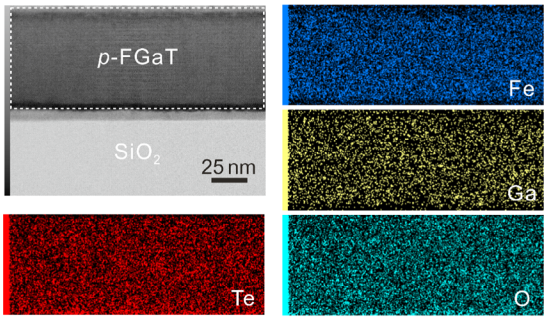

b

Elements	Atomic %
Fe	40.44
Ga	13.70
Te	30.10
O	15.76

Supplementary Fig. 42 | Quantitative elemental analysis of oxygen-implanted *p*-FGaT validating the theoretical model. a TEM image and corresponding large-area EDS elemental mapping of Fe, Ga, Te, and O. **b**

Quantitative atomic percentages of each element.

Comment-4: *Absence of the photocurrent in i-FGaT: The manuscript reports that the i-FGaT sample exhibits a significant second-harmonic generation (SHG) signal, indicating broken inversion symmetry. However, it remains unclear why this same sample does not exhibit a measurable photocurrent response. Given that SHG and the bulk photovoltaic effect (BPVE) are both sensitive to inversion symmetry breaking, a discussion is warranted to clarify the apparent discrepancy.*

Response: We thank the reviewer for highlighting this apparent discrepancy. The observation of SHG in *i*-FGaT without a corresponding measurable photocurrent is indeed intriguing, but it can be fully rationalized by considering the fundamental differences in both the origin of the symmetry breaking and the electronic ground state of the two samples.

First, regarding the SHG signal itself, we must clarify that the polar plots in Fig. 1c were normalized primarily to visualize the symmetry reduction rather than to compare absolute magnitudes. To address the reviewer's concern quantitatively, we have provided the raw intensity data in the new Supplementary Table 6. A direct comparison reveals that the maximum SHG intensity in *i*-FGaT is only ~312 counts, a weak signal characteristic of the trivial surface symmetry breaking ubiquitous in layered vdW crystals or local defects. In stark contrast, oxygen implantation in *p*-FGaT induces a robust bulk polar distortion, yielding a signal of ~4211 counts—over an order of magnitude (>13 times) stronger. This massive disparity confirms that the inversion symmetry breaking in *i*-FGaT is merely superficial, whereas *p*-FGaT possesses the substantial bulk polarity required for efficient nonlinear optical processes.

Second, and perhaps more critically, a strong SHG signal does not guarantee a large BPV current. Although both stem from second-order nonlinear susceptibilities, they are governed by different quantum geometric quantities. SHG depends on virtual transitions and the Berry curvature dipole, while the BPV shift current is dictated by the shift vector (R_{mn}) integrated over real interband transitions. It is well-established that these two parameters do not scale linearly.

Supplementary Table 6 | Raw data for angle-dependent SHG intensity of *i*-FGaT and *p*-FGaT.

***p*-FGaT**			***i*-FGaT**	
Angle (°)	Intensity (counts)		Angle (°)	Intensity (counts)
0	1169		0	276
10	1182		10	223
20	1341		20	145

30	1707		30	112
40	3578		40	135
50	3769		50	181
60	4211		60	234
70	3144		70	189
80	2378		80	126
90	1629		90	101
100	1506		100	170
110	842		110	215
120	963		120	214
130	518		130	143
140	993		140	146
150	2171		150	154
160	2796		160	242
170	2268		170	309
180	1951		180	301
190	1918		190	279
200	2146		200	179
210	2209		210	139
220	3384		220	151
230	3126		230	211
240	2540		240	202
250	2649		250	198
260	2442		260	140
270	2047		270	124
280	1269		280	148
290	1425		290	218
300	831		300	219
310	642		310	221
320	708		320	167
330	678		330	200
340	1104		340	209
350	1498		350	312
360	1693		360	295

This table presents measured SHG intensity (count values) for *p*-FGaT and *i*-FGaT across varying polarization angles (0°-360°). Whilst the normalized curve in the main text highlights symmetry variations, the raw data reveals substantial discrepancies in intensity values.

While the manuscript reports a very large photocurrent in this material at room temperature, I cannot recommend it for publication in its current form. To justify pianits potential, the authors must establish a clear connection between the observed photocurrent and the underlying magnetic order, demonstrating the material's magnetic tunability. Without such a link, the significance of the large photocurrent remains unclear.

Minor Comments

Comment-1: *Circular photogalvanic effect: Another intriguing aspect of the BPGE is its response to circularly polarized light. Given that the C3 symmetry is broken in this system, one would expect the circular photogalvanic effect to appear. It is unclear why the study does not include an investigation of this effect.*

Response: We agree with the reviewer that the broken C3 symmetry allows for the CPGE. To address this, we have performed CPGE measurements as part of our revised analysis. As shown in the Supplementary Fig. 41, a clear helicity-dependent photocurrent is observed, which follows the characteristic dependence on the phase angle. This CPGE signal confirms the presence of the injection current allowed by the breaking of time-reversal symmetry. However, the magnitude of this CPGE component is smaller compared to the linear polarization response (shift current). This explains why our initial focus was on the dominant shift current. We have now included the CPGE data in the Supplementary Materials to provide a complete picture of the symmetry-allowed transport mechanisms.

Comment-2: *Clarity on "PT-symmetry": In line 329, the authors mention seeing some effect of PT symmetry. Can this symmetry be attained in this material by changing the spin polarization? If not, then the meaning of the first few lines of the paragraph is unclear.*

Response: The reviewer raises a critical point regarding the symmetry classification. We agree that the reference to "*PT*-symmetry" in the original manuscript was imprecise and potentially misleading. Global *PT*-symmetry cannot be attained in our *p*-FGaT system simply by changing the spin polarization in its current ferromagnetic state.

In a ferromagnet, the spontaneous magnetization breaks time-reversal symmetry (*T*), while the oxygen gradient breaks inversion symmetry (*P*). Unlike specific antiferromagnetic states (e.g., bilayer CrI_3) where the combined *PT* symmetry is preserved, a ferromagnet lacks the sublattice compensation required to restore symmetry under the combined operation (since *PT* would map an up-spin to a down-spin, contradicting the FM order). Consequently, *PT* symmetry is inherently broken in *p*-FGaT.

This distinction is physically significant. As established in the literature (e.g.,

Nat. Commun. 10, 3783, 2019), systems preserving *PT* symmetry are forbidden from generating shift currents because the topological Berry phase is forced to vanish. Our observation of a giant short-circuit current density (~ 30 A·cm^{-2}) strongly suggests a dominant shift current mechanism. Therefore, the breaking of *PT* symmetry in our system is not just an incidental feature but a strict requirement that allows for the non-zero Berry phase connection driving this high-performance photovoltaic response. This interpretation aligns with the recent framework proposed by Asada et al. (Nat. Commun. 16, 4827, 2025) for MoS_2/$CrPS_4$ heterostructures. They demonstrated that in a regime where *P*, *T*, and *PT* are all broken, the photovoltaic response is a superposition of the dominant shift current (driven by geometric phase) and the magnetic injection current (driven by velocity asymmetry). Our observation of a magnetically tunable yet giant photocurrent is consistent with this coexistence regime.

We have revised the manuscript to remove the ambiguous terminology and correctly attribute the mechanism to the simultaneous breaking of *P*, *T*, and *PT* symmetries.

In the main text, we revised it as follows:

"Unlike *PT*-symmetric antiferromagnets where the shift current is symmetry-forbidden due to a vanishing Berry phase[21], the ferromagnetic *p*-FGaT inherently breaks *P*, *T*, and combined *PT* symmetries. This complete symmetry breaking lifts constraints on the Berry curvature, enabling a robust shift current response that coexists with magnetic injection currents, consistent with the observed giant photocurrent density[46]. ~~We explore the magnetic photovoltaic effect, where the alignment of magnetic order impacts *P*-symmetry while maintaining *PT*-symmetry[21, 46]. The polarization photocurrent is constrained by the disrupted asymmetry of Berry curvature. The preserved *PT*-symmetry has been observed in van der Waals magnets such as bilayer CrI_3[21], potentially influencing the current *p*-FGaT.~~"

Comment-3: *Clarity on "bipolar nature": The authors write in line 184: "Furthermore, the system exhibits bipolar semiconducting characteristics, with majority spins dominating the VBM and minority spins dominating the CBM." This is not clear from the band structure and DOS plot.*

Response: Thank you for pointing out this lack of clarity. We agree that the spin-resolved bipolar semiconducting character was not sufficiently evident from the original band structure and density-of-states (DOS) plots.

To clarify this point, we have recalculated the electronic structure using the hybrid HSE functional and present the updated spin-resolved band structure and DOS in Supplementary Fig. 44 The HSE results clearly demonstrate that

the valence band maximum (VBM) is dominated by majority-spin states, while the conduction band minimum (CBM) is dominated by minority-spin states, thereby confirming the bipolar semiconducting behavior described in the text. Based on these improved results, we have revised the corresponding figures and text to ensure that the spin-dependent nature of the VBM and CBM is explicitly and unambiguously illustrated.

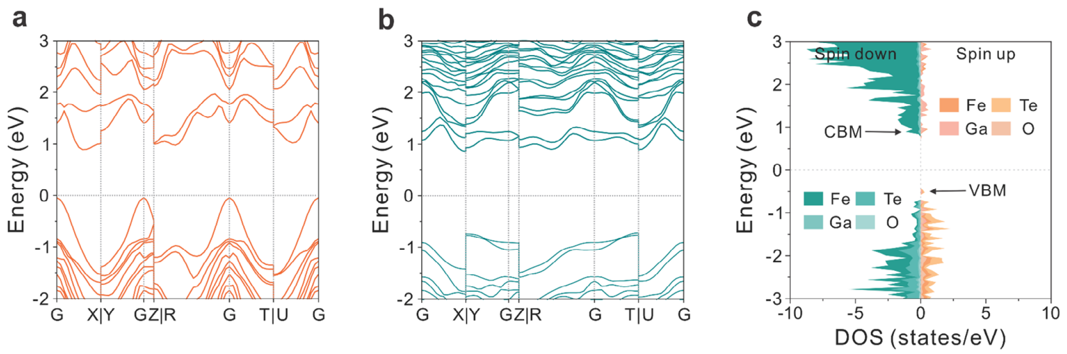

Fig. R1 | First-principles calculations and polar analysis of intralayer-oxygen-implanted *p*-FGaT. Spin-majority (Up) (**a**) and spin-minority (Down) (**b**) energy band structures, both demonstrating the nature of indirect semiconductor. **c** Spin-polarized density of states (DOS) diagram. The weights of different elements are identified with gradient colors, adopted from Fig. 2.

Comment-4: *Photocurrent vs. wavelength: It would be helpful if the authors presented the photocurrent as a function of light wavelength, similar to Figure 3(b). Does the photocurrent show sub-band responses?*

Response: We thank the reviewer for this constructive suggestion. We agree that presenting the raw photocurrent spectrum alongside the responsivity is crucial to validate the photoresponse, particularly in the sub-bandgap region.

In response to the reviewer's request, we have added Supplementary Fig. 43 to the revised Supplementary Information, which plots the raw photocurrent, the incident optical power density, and the derived responsivity as a function of wavelength. As clearly shown in Supplementary Fig. 43a, the device generates a substantial and measurable photocurrent not only in the visible range but also extending well into the near-infrared region (λ > 520 nm), which corresponds to photon energies below the intrinsic bandgap (E_g ≈ 2.39 eV). This observation confirms that the sub-bandgap response reported in the main text is a real physical signal arising from defect-mediated transitions or internal polar fields, rather than a measurement artifact or noise. Notably, first-principles calculations using the HSE functional reveal a fundamental electronic gap of ~1.02 eV (spin-up channel), which aligns well with this experimental cutoff. This confirms that the broadband BPV response extending to the near-infrared region originates from the intrinsic electronic structure captured by the hybrid

functional theory (Fig. 2).

To ensure the accuracy and reliability of the responsivity data presented in Fig. 3b and Supplementary Fig. 43c, we employed a rigorous calculation method based on precise calibration of the effective illuminated area. The spectral responsivity (R) was derived strictly from the measured photocurrent (I_{ph}) and optical power density ($P_{density}$) using the following equation,

$$R(\lambda) = \frac{I_{ph}(\lambda)}{P_{in}(\lambda)} = \frac{I_{ph}(\lambda)}{P_{density}(\lambda) \times A_{eff}}$$

where $I_{ph}(\lambda)$ is the measured photocurrent at each specific wavelength; $P_{density}(\lambda)$ is the wavelength-dependent optical power density of the excitation source (measured by a calibrated power meter, as shown in Supplementary Fig. 43b); A_{eff} is the effective photo-active area of the device.

In our experimental setup, the laser beam was focused to a spot diameter of 5 μm. Consequently, the effective area was calculated as $A_{eff} = \pi \times (2.5\ \mu m)^2 \approx 19.63\ \mu m^2$. We carefully ensured that the laser spot was fully confined within the device channel to maximize collection efficiency. This strict normalization process confirms that the observed spectral characteristics are intrinsic to the *p*-FGaT material.

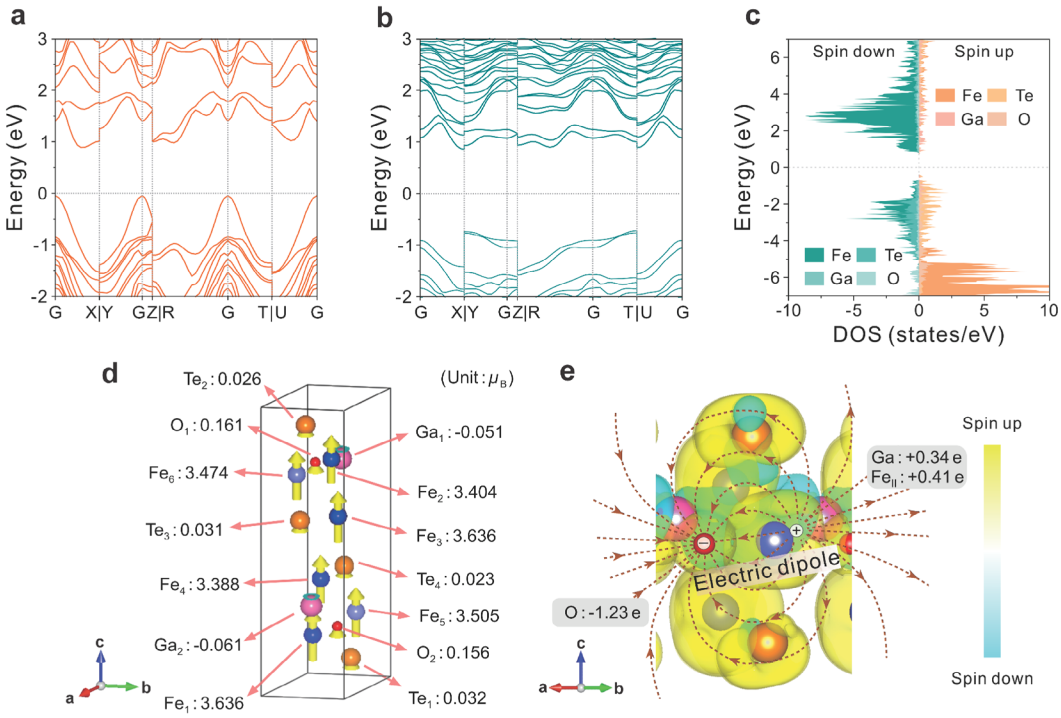

Fig. 2 | First-principles calculations and polar analysis of intralayer-oxygen-implanted *p*-FGaT. Spin-majority (Up) (**a**) and spin-minority (Down) (**b**) energy band structures, both demonstrating the nature of indirect semiconductor. **c** Spin-polarized density of states (DOS) diagram. The weights of different elements are identified with gradient colors. **d** Calculated Bohr magnetic moments and spin vector arrows of different atoms in the supercell. **e**

Illustrative electric dipole superimposed in the spin density distribution. The Bader charges of Fe_{II}, Ga, and O atoms are shown nearby.

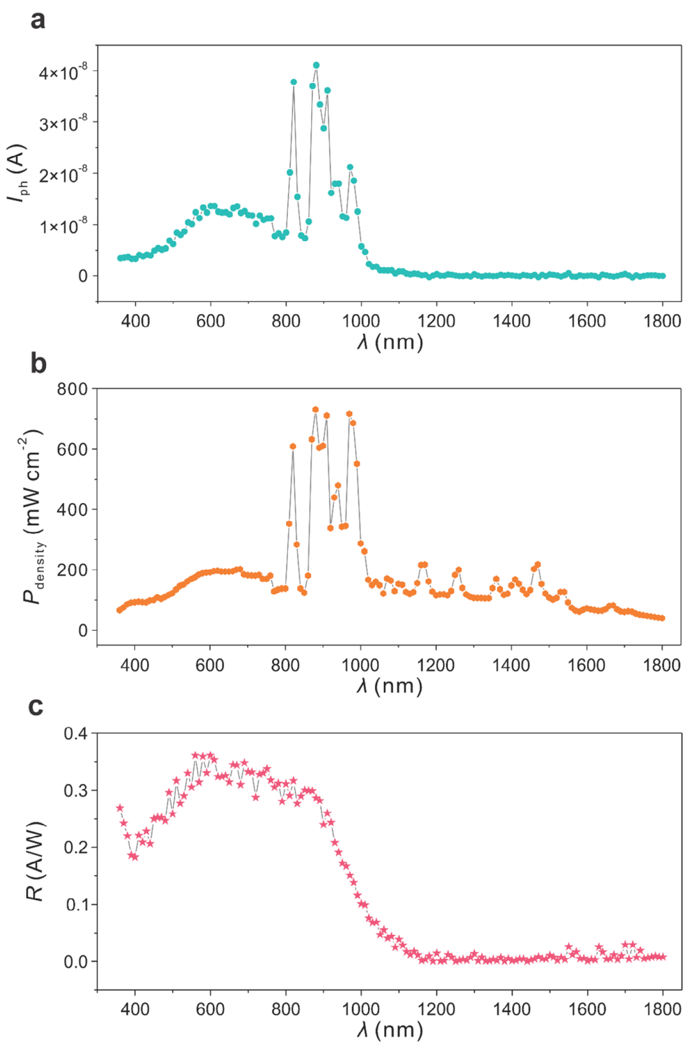

Supplementary Fig. 43 | Wavelength-dependent optoelectronic characteristics of the *p*-FGaT device. a Measured photocurrent (I_{ph}) spectrum ranging from 360 nm to 1800 nm. A distinct and measurable photoresponse is observed in the sub-bandgap region (λ > 520 nm), confirming the broadband detection capability. **b** Optical power density spectrum of the light source used for the measurement. The profile reflects the output characteristics of the excitation system. **c** Spectral responsivity (R) curve calculated from the data in **a** and **b**. The effective illumination area A_{eff} was determined to be ~19.63 μm^2 using a calibrated laser spot diameter of 5 μm.

**λ (nm)**	**I_{ph} (A)**	**$P_{density}$ (mW·cm^{-2})**	**R (A/W)**		**λ (nm)**	**I_{ph} (A)**	**$P_{density}$ (mW·cm^{-2})**	**R (A/W)**
360	3.46E-09	65.692	0.2687		1110	8.24E-10	150.285	0.0284
370	3.57E-09	75.193	0.2424		1120	4.21E-10	126.530	0.0169

380	3.69E-09	85.449	0.22		1130	2.81E-10	120.315	0.0119
390	3.34E-09	91.471	0.1862		1140	4.39E-10	126.042	0.0175
400	3.34E-09	93.044	0.1831		1150	3.51E-10	155.852	0.0112
410	4.10E-09	94.563	0.221		1160	5.93E-11	215.922	0.0014
420	3.81E-09	93.215	0.2087		1170	1.34E-10	217.372	0.0031
430	4.14E-09	92.470	0.2283		1180	-3.01E-10	161.038	0.0094
440	3.99E-09	98.763	0.2063		1190	2.91E-12	127.937	1.00E-04
450	4.90E-09	99.863	0.25		1200	3.26E-10	116.078	0.0142
460	5.40E-09	109.08	0.2524		1210	-1.09E-11	118.835	5.00E-04
470	5.14E-09	103.98	0.252		1220	-2.93E-11	119.134	0.0013
480	5.36E-09	110.631	0.2469		1230	2.68E-10	116.019	0.0116
490	6.82E-09	117.579	0.296		1240	1.88E-10	130.589	0.0072
500	6.21E-09	122.51	0.2584		1250	4.05E-12	182.602	1.00E-04
510	8.40E-09	135.36	0.3164		1260	-8.02E-11	200.108	0.002
520	7.95E-09	146.42	0.2768		1270	-1.01E-10	139.911	0.0036
530	8.64E-09	151.76	0.2902		1280	4.75E-11	118.950	0.002
540	1.04E-08	161.07	0.3298		1290	-1.47E-10	111.640	0.0067
550	1.01E-08	168.77	0.3055		1300	2.80E-10	107.076	0.0133
560	1.24E-08	175.24	0.361		1310	-1.85E-11	106.935	9.00E-04
570	1.13E-08	183.64	0.3138		1320	-1.53E-10	106.845	0.0073
580	1.33E-08	188.50	0.3592		1330	-1.71E-11	105.694	8.00E-04
590	1.24E-08	190.82	0.3301		1340	-1.93E-11	106.432	9.00E-04
600	1.36E-08	191.88	0.3613		1350	-9.68E-11	139.562	0.0035
610	1.36E-08	196.01	0.3535		1360	-4.98E-11	169.584	0.0015
620	1.25E-08	197.10	0.3237		1370	1.79E-10	135.531	0.0067
630	1.23E-08	194.55	0.3237		1380	1.42E-12	117.428	1.00E-04
640	1.24E-08	194.40	0.3254		1390	1.14E-10	121.245	0.0048
650	1.20E-08	194.32	0.3143		1400	3.71E-11	147.535	0.0013
660	1.32E-08	195.39	0.3448		1410	-5.58E-11	167.352	0.0017
670	1.35E-08	200.71	0.3439		1420	-1.25E-10	153.403	0.0042
680	1.23E-08	202.08	0.3099		1430	1.12E-10	134.041	0.0043
690	1.26E-08	184.51	0.348		1440	-3.05E-12	119.71	1.00E-04
700	1.18E-08	181.63	0.3322		1450	-6.64E-11	133.441	0.0025
710	1.18E-08	180.77	0.3315		1460	1.64E-10	202.522	0.0042
720	1.02E-08	180.25	0.2875		1470	-3.17E-10	217.861	0.0077
730	1.17E-08	182.81	0.3275		1480	1.37E-10	152.851	0.0045
740	1.10E-08	169.32	0.33		1490	-1.02E-10	121.815	0.0043
750	1.12E-08	169.51	0.3373		1500	2.29E-10	108.074	0.0107
760	1.12E-08	180.48	0.3174		1510	1.45E-10	101.352	0.0072
770	7.73E-09	129.02	0.3056		1520	3.49E-11	107.319	0.0016
780	8.24E-09	134.66	0.3121		1530	-1.69E-10	126.654	0.0068
790	7.56E-09	137.57	0.2802		1540	7.59E-11	126.732	0.0031
800	8.41E-09	137.87	0.3112		1550	4.81E-10	92.489	0.026
810	2.01E-08	353.01	0.2908		1560	-1.73E-10	74.1977	0.0118
820	3.78E-08	609.14	0.3165		1570	-2.09E-10	64.492	0.0165
830	1.54E-08	283.55	0.2766		1580	5.79E-11	61.3262	0.0048
840	7.84E-09	138.00	0.2897		1590	-6.47E-11	67.1528	0.0049
850	7.31E-09	124.02	0.3005		1600	-1.15E-11	72.8161	8.00E-04
860	1.06E-08	180.51	0.2995		1610	4.82E-11	69.007	0.0036

870	3.70E-08	631.53	0.2988		1620	-3.65E-11	66.2421	0.0028
880	4.11E-08	731.26	0.2864		1630	-3.19E-10	64.2191	0.0253
890	3.34E-08	604.60	0.2819		1640	2.06E-10	64.6973	0.0162
900	2.88E-08	610.87	0.2401		1650	5.70E-11	69.8095	0.0041
910	3.61E-08	711.161	0.2593		1660	-7.30E-11	80.3021	0.0046
920	1.62E-08	338.48	0.2437		1670	-1.81E-10	82.0679	0.0113
930	1.79E-08	439.92	0.2081		1680	-4.41E-11	69.2785	0.0032
940	1.80E-08	480.25	0.1908		1690	1.20E-10	62.2988	0.0098
950	1.16E-08	343.1	0.1726		1700	3.45E-10	60.2188	0.0291
960	1.13E-08	345.78	0.1671		1710	5.07E-11	62.5449	0.0041
970	2.12E-08	717.24	0.1506		1720	-3.49E-10	60.9452	0.0293
980	1.86E-08	685.89	0.1381		1730	7.20E-11	54.7055	0.0067
990	1.25E-08	550.73	0.1161		1740	-1.92E-10	51.656	0.0189
1000	5.73E-09	286.70	0.1009		1750	-4.53E-11	48.9523	0.0047
1010	4.65E-09	261.17	0.0991		1760	5.52E-11	46.80	0.006
1020	2.29E-09	166.35	0.0759		1770	6.40E-11	44.5885	0.0073
1030	1.77E-09	149.29	0.0678		1780	7.67E-11	42.824	0.0091
1040	1.80E-09	160.16	0.0684		1790	-5.92E-11	41.1298	0.0074
1050	1.09E-09	148.69	0.0474		1800	-5.87E-11	39.4455	0.0076
1060	1.07E-09	121.96	0.0552					
1070	1.03E-09	170.61	0.0409					
1080	1.09E-09	163.56	0.0439					
1090	4.52E-10	129.72	0.0247					
1100	8.55E-10	153.36	0.0386					

References

1. Cohen AJ, Mori-Sánchez P, Yang W. Insights into Current Limitations of Density Functional Theory. *Science* **321**, 792-794 (2008).

2. Bao JL, Gagliardi L, Truhlar DG. Self-Interaction Error in Density Functional Theory: An Appraisal. *The Journal of Physical Chemistry Letters* **9**, 2353-2358 (2018).

3. Heyd J, Scuseria GE, Ernzerhof M. Hybrid functionals based on a screened Coulomb potential. *The Journal of Chemical Physics* **118**, 8207-8215 (2003).

4. Henderson TM, Paier J, Scuseria GE. Accurate treatment of solids with the HSE screened hybrid. *physica status solidi (b)* **248**, 767-774 (2011).

5. Wróbel J, Kurzydłowski KJ, Hummer K, Kresse G, Piechota J. Calculations of ZnO properties using the Heyd-Scuseria-Ernzerhof screened hybrid density functional. *Physical Review B* **80**, 155124 (2009).

6. Flores MA, Orellana W, Menéndez-Proupin E. Accuracy of the Heyd-Scuseria-Ernzerhof hybrid functional to describe many-electron interactions and charge localization in semiconductors. *Physical Review B* **98**, 155131 (2018).

7. Zhang YJ*, et al.* Enhanced intrinsic photovoltaic effect in tungsten disulfide nanotubes. *Nature* **570**, 349-353 (2019).

8. Jiang J*, et al.* Flexo-photovoltaic effect in MoS_2. *Nature Nanotechnology* **16**, 894-901 (2021).

9. Nechache R*, et al.* Photovoltaic properties of Bi_2FeCrO_6 epitaxial thin films. *Applied Physics Letters* **98**, (2011).

10. Osterhoudt GB*, et al.* Colossal mid-infrared bulk photovoltaic effect in a type-I Weyl semimetal. *Nature Materials* **18**, 471-475 (2019).

11. Zhang G*, et al.* New high Tc multiferroics $KBiFe_2O_5$ with narrow band gap and promising photovoltaic effect. *Scientific Reports* **3**, 1265 (2013).

12. Choi T, Lee S, Choi YJ, Kiryukhin V, Cheong SW. Switchable ferroelectric diode and photovoltaic effect in $BiFeO_3$. *Science* **324**, 63-66 (2009).

13. Matsuo H, Noguchi Y, Miyayama M. Gap-state engineering of visible-light-active ferroelectrics for photovoltaic applications. *Nature Communications* **8**, 207 (2017).

14. Astafiev SB, Fridkin VM, Lazarev VG. The Influence of the magnetic-field on the linear bulk photovoltaic current in piezoelectric semiconductor GaP. *Ferroelectrics* **80**, 899-902 (1988).

15. Ichiki M, Maeda R, Morikawa Y, Mabune Y, Nakada T, Nonaka K. Photovoltaic effect of lead lanthanum zirconate titanate in a layered film structure design. *Applied Physics Letters* **84**, 395-397 (2004).

16. Zenkevich A, Matveyev Y, Maksimova K, Gaynutdinov R, Tolstikhina A, Fridkin V. Giant bulk photovoltaic effect in thin ferroelectric $BaTiO_3$ films. *Physical Review B* **90**, 161409 (2014).

17. Fridkin VM, Grekov AA, Rodin AI. The bulk photovoltaic effect in the crystals without a center of symmetry. *Ferroelectrics* **43**, 99-108 (1982).

18. Laurenti M*, et al.* Nanobranched ZnO structure: p-type doping induces piezoelectric voltage generation and ferroelectric–photovoltaic effect. *Advanced Materials* **27**, 4218-4223 (2015).

19. Sturman BI, Firdkin VM. Photovoltaic and photo-refractive effects in noncentrosymmetric materials. *Ferroelectrics*, (1992).

20. Mistewicz K, Nowak M, Stróż D. A Ferroelectric-photovoltaic effect in SbSI nanowires. *Nanomaterials* **9**, 580 (2019).

21. Nadupalli S, Kreisel J, Granzow T. Increasing bulk photovoltaic current by strain tuning. *Science Advances* **5**, eaau9199 (2019).

22. Burger AM*, et al.* Direct observation of shift and ballistic photovoltaic currents. *Science Advances* **5**, eaau5588 (2019).

Response to Reviewers' Comments

Reviewer #1

General Comment: *In their manuscript entitled "Room-Temperature Magnetically Tunable Iron-Based Photovoltaics" by M. Cheng and co-workers, the authors have appropriately revised the manuscript according to my questions and comments. I think that the authors have made major revisions to meet the higher standards and criteria for publication in Nature Communications. Thus, I recommend publication in Nature Communications.*

Reply: We thank the reviewer for the positive assessment and for recognizing that our major revisions have met the high standards of Nature Communications. We are grateful for your recommendation for publication.

Reviewer #2

General Comment: *The authors have addressed my questions with full details and the manuscript can be accepted as it is now.*

Reply: We are pleased to have provided a detailed response that answered all your questions. Thank you for recommending this manuscript for acceptance.

Reviewer #3

General Comment: *The authors have addressed several of my earlier questions regarding modeling of oxygen incorporation and the relation of photocurrent and SHG in i-FGT. The new data on CPGE and sub-gap response are valuable addition to the manuscript. These revisions improve the completeness of experimental characterization.*
Despite these improvements, my two primary concerns regarding the claim of magnetic injection current remain unresolved.

The authors seem to attribute a portion of the ~20% reduction in photocurrent (compared to ~8% reduction in the dark condition) in the presence of the magnetic field to a linear magnetic injection current, stating in the main text that the PMR alone cannot explain it. This is qualitatively contradicting the definition of magnetic injection current. The magnetic injection current will change sign upon reversing the magnetic order. However, experiments show the same sign of current change for + and - fields. There is no way to rationalize this is a magnetic injection effect.

The authors present new CPGE data and argue that its observation supports time-reversal-symmetry–broken injection current. This is not true. CPGE occurs

as an injection current in nonmagnetic systems. But they studied a magnetic material here. There is no direct connection between CPGE and claimed magnetic injection current in this work.

In conclusion, I stress two things. (a) Since the suppression of photocurrent is argued to be related to the MR, the authors should show a plot of the MR with the magnetic field to support this interpretation. (b) The authors should explicitly state that if magnetic injection current were present, there should be opposite signs between photocurrent measured at positive and negative magnetic fields.

With these clarifications, I would be in a better position to comment on the suitability of the manuscript for publication.

Reply: We are genuinely grateful for the reviewer's sharp physical insight regarding the symmetry of the current response. We fully agree with your assessment: the fact that we observe symmetric current suppression under both positive and negative magnetic fields is indeed incompatible with the strict definition of a magnetic injection current, which would require a sign reversal upon reversing the magnetic order. We also acknowledge that CPGE signals can arise in non-magnetic systems and do not uniquely prove the existence of magnetic injection current. Consequently, we have revised the manuscript to decouple the CPGE data from our claims regarding the transport mechanism. Prompted by this re-evaluation, we have shifted our focus to a more robust physical interpretation based on the Positive Magnetoresistance (PMR) effect. As shown in the newly added Supplementary Fig. 42, the dark current (I_d) exhibits a characteristic symmetric decrease with increasing magnetic field, confirming an intrinsic PMR effect. Crucially, under illumination, the net photocurrent ($\Delta I = I_{ph} - I_d$) follows this exact same symmetric trend. This consistency demonstrates that the photocurrent suppression is not driven by a symmetry-broken injection current, but is a direct consequence of the PMR effect modulating the transport of both equilibrium and photogenerated carriers.

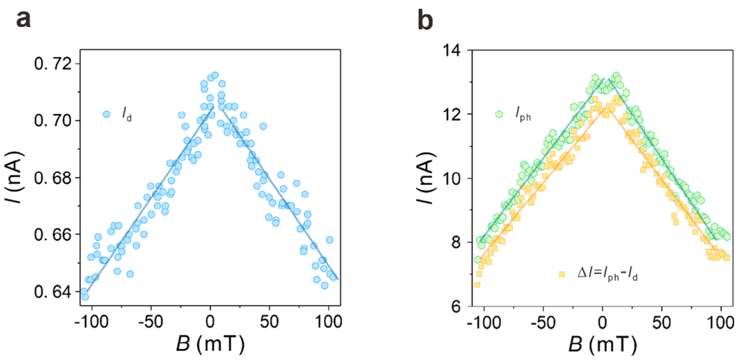

Supplementary Fig. 42 | Comparative analysis of magnetic field-dependent transport in dark and illuminated states. a Magnetic field

dependence of the longitudinal current measured in the dark (I_d), exhibiting a characteristic positive magnetoresistance (PMR) effect where the current is symmetrically suppressed by the external magnetic field. **b** The magnetic field dependence of the net photocurrent is obtained by subtracting the dark current background from the total current under illumination ($\Delta I = I_{ph} - I_d$). Measurements were performed using a 520 nm laser with a power density of 5 mW mm^{-2}. The net photocurrent exhibits symmetric modulation, perfectly matching the symmetry of the dark magnetoresistance effect shown in **a**.

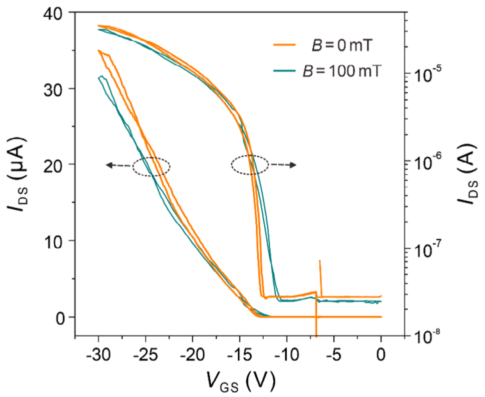

Supplementary Fig. 43 | Transfer characteristics (I_{DS}~V_{GS}) of the *p*-FGaT field-effect transistor (FET) under zero magnetic field (B = 0 mT) and an applied perpendicular magnetic field (B = 100 mT). The linear-scale I_{DS}~V_{GS} curves (left axis) reveal a distinct decrease in transconductance ($g_m = dI_{DS}/dI_{GS}$) upon magnetic field application, while the semi-logarithmic I_{DS}~V_{GS} curves (right axis) indicate a positive shift in threshold voltage (V_{th}) from -12.5 V to -10.6 V and a degradation in subthreshold slope [SS= $dV_{GS}/d(\log_{10}I_{DS})$]. The 2D hole density ($n$) at a specific gate bias (V_{GS} = -30 V) is calculated based on the MOS capacitor model ($n = C_i|V_{GS}-V_{th}|/e$). All measurements were performed at a constant drain-source voltage V_{DS} of 10 mV.

Based on the above equations, the fundamental parameters can be calculated as follows.

Conditions	V_{th} (V)	μ_{FE} (cm^2 V^{-1}s^{-1})	n (cm^{-2})	SS (mV dec^{-1})
B = 0 mT	-12.5	290.7	1.26×10^{12}	308.7
B = 100 mT	-10.6	246.0	1.40×10^{12}	415.4

To further elucidate the microscopic origin of this PMR, we performed field-effect transistor (FET) characterizations under a 100 mT magnetic field (Supplementary Fig. 43). A notable reduction in μ_{FE} from 290.7 to 246.0 cm^2 V^{-1}s^{-1} (~15.4% degradation) is observed at B = 100 mT. This degradation is primarily attributed to the Lorentz force-induced carrier deflection, which increases the effective scattering rate and lengthens the mean free path trajectory (geometric magnetoresistance effect). Interestingly, the application of the magnetic field leads to a positive shift in V_{th} (from -12.5 V to -10.6 V). This

shift results in a higher calculated carrier density (1.40×10^{12} cm^{-2}) at V_{GS} = -30 V compared to the zero-field condition. The V_{th} shift may originate from magnetic-field-dependent trapping/detrapping mechanisms at the semiconductor/dielectric interface or a redistribution of localized states. The SS increases from 308.7 to 415.4 mV dec^{-1}, indicating an increased hindrance in the transition from the "off" to the "on" state. This suggests that the magnetic field not only affects the steady-state transport (mobility) but also influences the percolation or hopping dynamics during the initial channel formation. Despite the higher carrier concentration at B = 100 mT, the device exhibits a lower I_{DS}, confirming that the mobility suppression dominates the overall electrical performance in the presence of a magnetic field.

Finally, regarding the magnitude difference (~20% suppression in light vs. ~8% in dark), this is physically consistent within the PMR framework: high-energy, non-equilibrium photogenerated carriers typically exhibit distinct scattering cross-sections and are more susceptible to magnetic-field-dependent scattering compared to thermally relaxed equilibrium carriers.

In light of these findings, we have withdrawn the claim of "magnetic injection current" and explicitly attributed the observed phenomena to the PMR effect supported by the new magnetoresistance and FET data.

The modifications are as follows:

Semiconducting and BPV properties of *p*-FGaT device

~~In magnetic system, the injection current is also highly sensitive to the linearly-polarized excitation[20, 21, 46]. Therefore, the generated photocurrent is comprised by the shift current and injection current, revealing a synergistic contribution of *P*- and *T*-symmetry breaking to the BPV behavior.~~ While injection-type contributions are symmetry-allowed in magnetic systems[20, 21, 46], their identification requires field-odd photocurrent signatures that are not observed here (as discussed later). Accordingly, the photocurrent is interpreted as dominated by shift-current generation together with magnetic-field-dependent transport modulation.

Magnetic photovoltaic sensing applications

The magnetic field-modulated photocurrent experiment setup (Fig. 4a) involves a 520 nm laser illuminating vertically onto the BPV sensor. An electromagnet positioned below the sensor generates an out-of-plane magnetic field perpendicular to the sensor, with adjustable field strength controlled by the coil current. Current and magnetic field signals are recorded simultaneously by an integrated software system. ~~Fig. 4b illustrates an 8% reduction in the dark current of the BPV sensor under ±100 mT magnetic fields, indicating a positive magnetoresistance (PMR) effect that remains consistent for both polarities. Upon further illumination, the short-circuit photocurrent decreased by 20%~~

under the same magnetic field, suggesting that the suppression of magnetic field on photocurrent is not solely due to the PMR effect. Notably, the injection current, arising from breaking the momentum-inversion symmetry in the band structure, is sensitive to changes in magnetic order and may affect photocurrent modulation[20, 46]. Fig. 4b illustrates a symmetric reduction (~8%) in the dark current of the BPV sensor under ±100 mT magnetic fields, indicating an intrinsic positive magnetoresistance (PMR) effect that remains consistent for both polarities.Upon illumination, the short-circuit photocurrent exhibits a similar symmetric suppression, but with a significantly enhanced modulation depth of approximately 20% under the same magnetic field (Supplementary Fig. 42). This amplification suggests that non-equilibrium photo-generated carriers are more sensitive to the magnetic-field-induced scattering than thermally relaxed equilibrium carriers. As further elucidated by our field-effect transistor (FET) characterizations (Supplementary Fig. 43), the magnetic field leads to a notable degradation in field-effect mobility (μ_{FE}) while slightly increasing carrier density, confirming that mobility suppression dominates the overall current reduction. Fig. 4c depicts variations in photocurrent under periodic magnetic field steps. The rapid response of photocurrent to magnetic fields is attributed to the strong built-in electric field and high hole mobility in *p*-FGaT. Both positive and negative magnetic fields similarly inhibit photocurrent. However, beyond the saturation magnetic field (B_s = 120 mT), the decrease in photocurrent tends to plateau (Supplementary Fig. 36), diverging from the expected photoelectromagnetic (PEM) effect[74]. The PEM effect, driven by the Lorentz force on photocarriers in a magnetic field, typically induces a bias effect on spontaneous photocurrents but does not exhibit field-related saturation or suppression symmetry. As magnetic domain orientations align, the decrease in domain walls reduces spin scattering interfaces, making it challenging to explain the suppression of photocurrents. Fig. 4c depicts variations in photocurrent under periodic magnetic field steps, demonstrating a rapid and reversible response. Notably, both positive and negative magnetic fields inhibit the photocurrent with identical symmetry, which is fundamentally inconsistent with the time-reversal-symmetry-broken injection current (which would mandate a sign reversal) but perfectly aligns with the PMR-based transport modulation. Beyond the saturation magnetic field (B_s = 120 mT), the decrease in photocurrent tends to plateau (Supplementary Fig. 36 and 40), a behavior that diverges from the photoelectromagnetic (PEM) [74] effect but closely correlates with the magnetic ordering of *p*-FGaT.

Unlike *PT*-symmetric antiferromagnets where the shift current is symmetry-forbidden due to a vanishing Berry phase[21], the ferromagnetic *p*-FGaT inherently breaks *P*, *T*, and combined *PT* symmetries. This complete symmetry breaking lifts constraints on the Berry curvature, enabling a robust shift current response that coexists with magnetic injection currents, consistent with the observed giant photocurrent density[46]. Unlike *PT*-symmetric antiferromagnets where the shift current is symmetry-forbidden, the ferromagnetic *p*-FGaT

inherently breaks *P*, *T*, and combined *PT* symmetries. This complete symmetry breaking lifts constraints on the Berry curvature[21], enabling a robust shift current response that serves as the foundation for the observed giant photocurrent density[46].

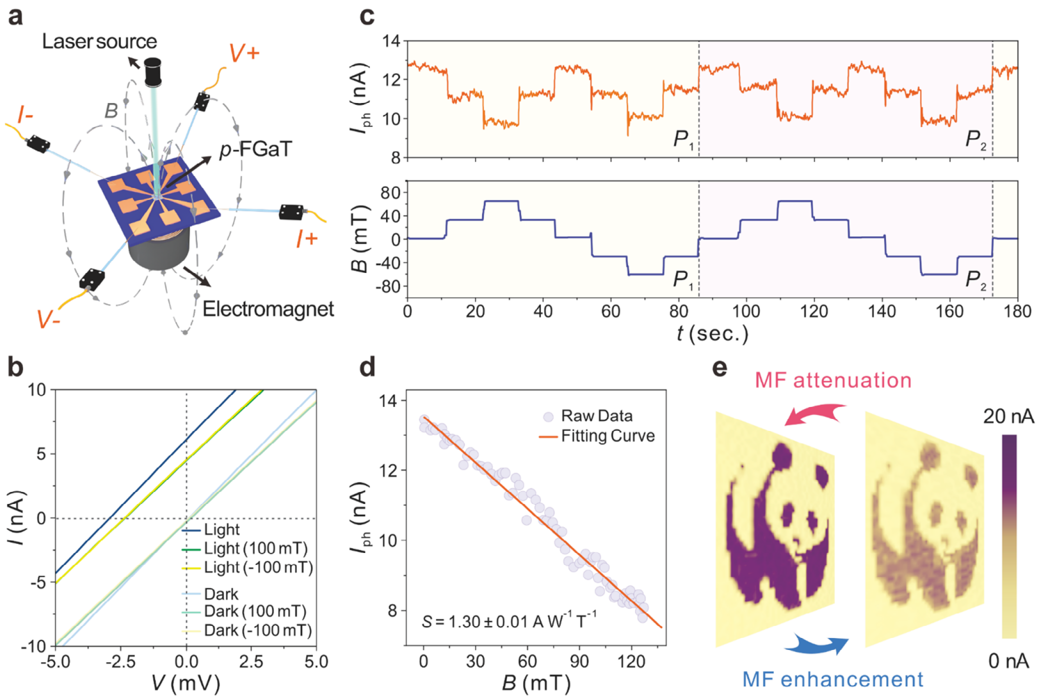

Fig. 4 | Mageto-optoelectronic response tests of *p*-FGaT device. a Schematic illustration of magnetic field modulated photocurrent experiments. **b** Current-voltage (*I*-*V*) curves measured under ±100 mT magnetic field with dark and light conditions (λ = 520 nm, *P* = 2.5 mW mm^{-2}). **c** Stepwise programmable magnetic field (*B*) modulation and simultaneous photocurrent (I_{ph}) response. **d** Linear sensing characteristic of I_{ph} versus *B* with a sensitivity of 1.30 ± 0.01 A W^{-1}T^{-1}. **e** Comparison of photoelectric imaging before and after application of a fixed magnetic field (*B* = 120 mT). Illumination conditions in **c**-**e** are λ = 520 nm and *P* = 5 mW mm^{-2}.

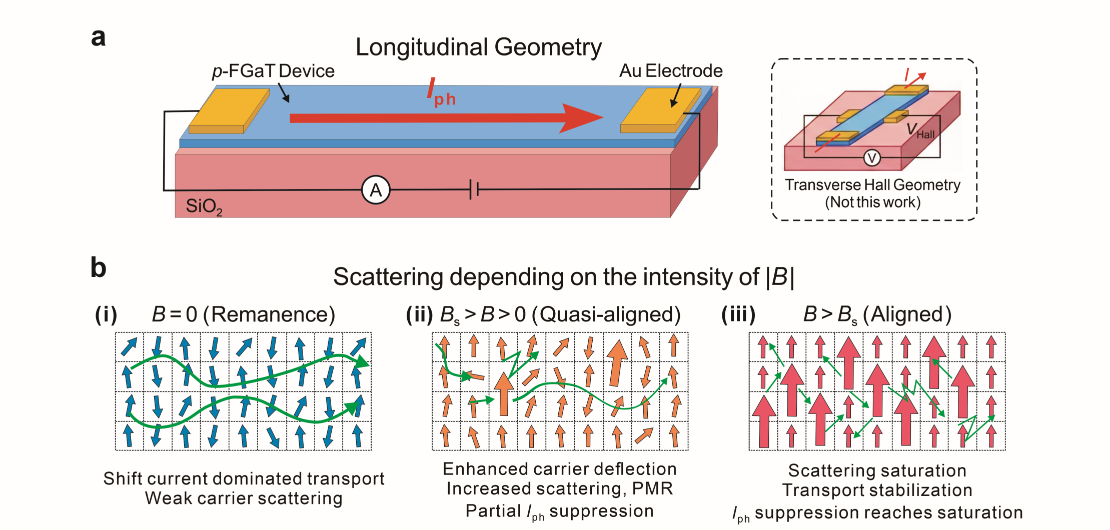

Supplementary Fig. 39 | Schematic illustration of magnetic-field modulation of photocurrent in *p*-FGaT devices. a Device geometry used for longitudinal photocurrent measurements under illumination and applied magnetic field. **b** Conceptual depiction of carrier transport evolution with increasing magnetic field.

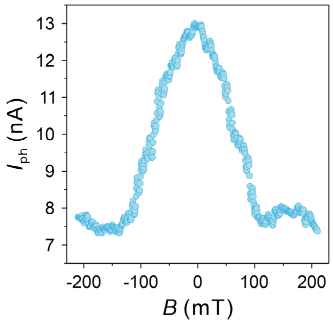

Supplementary Fig. 40 | Photocurrent response under magnetic field. Illumination conditions are λ = 520 nm and P = 5 mW mm^{-2}.

To further probe the symmetry characteristics of the photoresponse, circular photogalvanic effect (CPGE) measurements were performed. As shown in Supplementary Fig. 41, a helicity-dependent photocurrent oscillating with the phase angle of the quarter-wave plate is observed. This behavior reflects asymmetric carrier excitation processes enabled by broken spatial and magnetic symmetries.

We emphasize that CPGE can arise in both magnetic and nonmagnetic systems and therefore does not constitute direct evidence for magnetic injection current. Instead, the measurement serves as complementary characterization confirming helicity-sensitive optical transitions and the presence of symmetry conditions relevant to nonlinear photoresponse. The macroscopic linear

photocurrent discussed in the main text remains dominated by shift-current and transport-modulation contributions.

Response to Reviewers' Comments

Reviewer #3

General Comment: *The authors revised their interpretation on the magnetic BPVE and changed it to ordinary magnetoresistance. This might be solid in theory. But, in this sense, this phenomenon reported in this work looks very trivial.*

Reply: We thank the reviewer for this important comment. We agree that the magnetic-field-dependent photocurrent modulation should not be interpreted as magnetic BPVE or as an injection-current-dominated BPV contribution. We have therefore revised the manuscript to clearly distinguish the BPV generation mechanism from the magnetic-field-dependent output modulation. The zero-bias BPV photocurrent is attributed primarily to oxygen-induced inversion-symmetry breaking and shift-current-dominated generation, while the magnetic field modulates this photocurrent through magnetoresistive transport.

In the previous version, we discussed the possible contribution of injection current to the magnetic-field-dependent photocurrent response. After further reassessment, we found that the experimental signatures do not support an injection-current-dominated interpretation. In particular, the photocurrent suppression is symmetric under positive and negative magnetic fields, whereas an injection-current-dominated magnetic BPV contribution would be expected to exhibit a field-odd signature. In addition, field-effect transistor measurements under magnetic field show a pronounced decrease in field-effect mobility accompanied by only a slight increase in carrier density, supporting mobility suppression of photo-generated carriers as the dominant factor responsible for the photocurrent reduction. Accordingly, the revised manuscript now describes the magnetic-field-dependent response as magnetoresistive modulation of the shift-current-generated photocurrent output.

With this clarification, we have shifted the focus of the manuscript to the main advance of the work: oxygen-plasma implantation converts a room-temperature iron-based van der Waals magnetic metal with negligible zero-bias photoresponse into a ferromagnetic semiconductor exhibiting a giant BPV effect. Pristine Fe_3GaTe_2 is a layered metallic ferromagnet with robust magnetic ordering above room temperature, but it shows negligible photoresponse under the same measurement conditions. By introducing oxygen into the Fe_3Ga slabs, itinerant Fe *d* electrons are localized, p-type semiconducting transport is induced, non-centrosymmetric polar distortions are generated, and ferromagnetic ordering is retained above room temperature. These combined effects enable a giant, broadband, zero-bias BPV response dominated by shift-current generation.

We respectfully note that the significance of the work does not lie in the magnetic-field-dependent modulation itself. Instead, the central discovery is the artificial creation of a giant BPV effect from an originally photo-inactive room-temperature iron-based van der Waals magnetic metal through oxygen-plasma implantation. The resulting BPV coefficient reaches 0.25 V^{-1}, exceeding conventional bulk ferroelectrics and comparable to state-of-the-art low-dimensional BPV systems. This result provides a materials strategy for generating strong BPV responses in magnetic semiconductors, where room-temperature ferromagnetism and non-centrosymmetric photovoltaic generation coexist in a single-phase system.

Following the reviewer's concern and the additional reviewer's suggestion, we have revised the title, abstract, introduction, Fig. 3 discussion, Fig. 4 discussion, in-sensor vision section, and summary to reflect this revised focus. The revised manuscript now emphasizes giant bulk photovoltaics in an iron-based van der Waals ferromagnetic semiconductor, while the magnetic-field-dependent photocurrent modulation is treated as a magnetoresistive output/readout effect. The term "photo-magnetoelectric" is used to describe the device-level functionality that integrates BPV-based optical generation with

magnetoresistive magnetic-field readout, rather than direct magnetic control of BPV generation.

We hope that these revisions address the reviewer's concern and clarify that the nontrivial aspect of this work lies in the oxygen-engineered giant self-powered BPV response in a room-temperature van der Waals ferromagnetic semiconductor, together with its use for photo-magnetoelectric sensing and magnetic-field-programmable in-sensor computing.

Reviewer #2

General Comment: *The authors report the room-Temperature bulk photovoltaic effects in p-FGaT iron-based devices. The magnetic tunability may be not precise as Reviewer 3 mentioned and the authors have changed that to ordinary magnetic resistance in revision. I think this work may not report magnetic tunability of bulk photovoltaics, but it is still very interesting in reporting an excellent bulk photovoltaic effect which has one of the best bulk photovoltaic coefficient as high as 0.25 V^{-1}, several orders of magnitude higher than conventional bulk ferroelectrics (typically 10^{-4}-$10^{-5}V^{-1}$) and comparable to state-of-the-art low-dimensional systems such as WS_2 nanotubes. This giant bulk photovoltaic performance in van der Waals ferromagnetic semiconductor is very important for potential photo-magnetoelectric applications. I think although the absence of magnetic tenability in bulk p-FGaT is a pity but this work is still very important with significant new insight in bulk photovoltaics in ferromagnetic semiconductors. I think after changing to new focus in bulk photovoltaics in van der Waals ferromagnetic semiconductors, the work deserves to publish in Nature Communications.*

Reply: We sincerely thank the reviewer for the positive and constructive assessment of our work. We are grateful that the reviewer recognizes the significance of the giant bulk photovoltaic (BPV) response in *p*-FGaT and its

potential importance for photo-magnetoelectric applications. The reviewer's suggestion to refocus the manuscript on bulk photovoltaics in van der Waals ferromagnetic semiconductors has been very helpful in reshaping the central message of the work.

Following this suggestion, we have substantially revised the manuscript to place the main emphasis on the artificial creation of giant room-temperature BPV in an iron-based van der Waals ferromagnetic semiconductor. The revised title, abstract, introduction, results discussion, and summary now emphasize that oxygen-plasma implantation converts pristine Fe_3GaTe_2, a room-temperature layered magnetic metal with negligible zero-bias photoresponse, into a p-type ferromagnetic semiconductor with oxygen-induced non-centrosymmetric polar distortions. These structural and electronic changes enable shift-current-dominated BPV generation while preserving ferromagnetic ordering above room temperature.

In this revised framework, the main advance of the work is the oxygen-engineered emergence of a giant, broadband, zero-bias BPV response in a magnetic semiconductor platform. The BPV coefficient reaches 0.25 V^{-1}, exceeding conventional bulk ferroelectrics and comparable to state-of-the-art low-dimensional BPV systems, as the reviewer noted. We believe this result provides significant insight into how strong BPV responses can be artificially introduced into van der Waals magnetic materials through symmetry and electronic-structure engineering.

We have also clarified the role of the magnetic field to avoid overclaiming. The magnetic-field-dependent photocurrent change is now described as magnetoresistive modulation of the photocurrent output, rather than magnetic tuning of the BPV generation mechanism. This clarification allows the photo-magnetoelectric application perspective to be retained at the device-function level: the BPV effect provides self-powered optical generation, while magnetoresistive transport provides magnetic-field-dependent readout and programmability.

We appreciate the reviewer’s constructive recommendation, which has guided the revised framing of the manuscript. We believe the revised version now more accurately presents the key contribution of the work: the realization of giant bulk photovoltaics in a room-temperature iron-based van der Waals ferromagnetic semiconductor, together with its potential utility for photo-magnetoelectric sensing and programmable in-sensor optoelectronics.
